# DVT: a high-throughput analysis pipeline for locomotion and social behavior in adult *Drosophila melanogaster*

**DOI:** 10.1186/s13578-023-01125-0

**Published:** 2023-10-05

**Authors:** Kai Mi, Yiqing Li, Yuhang Yang, Julie Secombe, Xingyin Liu

**Affiliations:** 1 Department of Pathogen Biology-Microbiology Division, State Key Laboratory of Reproductive Medicine and Offspring Health, Key Laboratory of Pathogen of Jiangsu Province, Key Laboratory of Human Functional Genomics of Jiangsu Province, Center of Global Health, Nanjing Medical University, Nanjing, 211166 China; 2 The Affiliated Suzhou Hospital of Nanjing Medical University, Suzhou Municipal Hospital, Gusu School, Nanjing Medical University, Suzhou, China; 3 Dominick P. Purpura Department of Neuroscience, Albert Einstein College of Medicine, Bronx, NY USA

**Keywords:** *Drosophila* video tracking (DVT), Fly behaviors, Chamber design

## Abstract

**Background:**

*Drosophila melanogaster* is excellent animal model for understanding the molecular basis of human neurological and motor disorders. The experimental conditions and chamber design varied between studies. Moreover, most previously established paradigms focus on fly trace detection algorithm development. A comprehensive understanding on how fly behaves in the chamber is still lacking.

**Results:**

In this report, we established 74 unique behavior metrics quantifying spatiotemporal characteristics of adult fly locomotion and social behaviors, of which 49 were newly proposed. By the aiding of the developed analysis pipeline, *Drosophila* video tracking (DVT), we identified siginificantly different patterns of fly behavior confronted with different chamber height, fly density, illumination and experimental time. Meanwhile, three fly strains which are widely used as control lines, Canton-S(CS), *w*^*1118*^ and Oregon-R (OR), were found to exhibit distinct motion explosiveness and exercise endurance.

**Conclusions:**

We believe the proposed behavior metrics set and pipeline should help identify subtle spatial and temporal differences of drosophila behavior confronted with different environmental factors or gene variants.

**Supplementary Information:**

The online version contains supplementary material available at 10.1186/s13578-023-01125-0.

## Background

Over the past several decades, behavior research using *Drosophila melanogaster* has become an established way to understand the etiology of motor function degenerative disorders [1], neurological disorders diseases such as Alzheimer’s disease [2], Parkinson’s disease [3] and Autism spectrum disorders [4], in addition to metabolic disorders diseases [5].

With advancement of machine learning algorithms and computer vision technologies, many fly-specific or general-purpose video-based high-throughput behavioral analysis paradigms have become available in recent years. Early methods were devoted to behavior state or event recording, for example, Jwatcher [6] was used to count the amount of time spent at the arena edge [7]. In 2009, Branson, et al. proposed the first automated, quantitative and high-throughput system, Ctrax [8], for position tracking of unmarked flies. This was followed by EasyFlyTracker [9], Buridan’s Paradigm with CeTrAn [10], IowaFLI Tracker [11], Flytracker [12] which were able to track the position of unmarked individual or multiple flies. Gal et al. developed anTraX for marked or color-tagged flies tracking [13]. In addition to fly-specific position tracking, general-purpose programs such as ToxTrac [14], UMATracker [15], FIMTrack [16, 17], ilastik [18] have been used for tracking flies, bees, mice, zebrafish and other animals. Additionally, Deeplabcut [19], Deeplabcut2 [20], SLEAP [21], idtracker.ai [22] gained popularity, all of which focused on body pose estimation and position tracking of animals via deep neural networks.

Most of existing paradigms focused on fly trace detection algorithm development. Panadeiro et al. made a survey on 28 video tracking software and only four of them can directly export in-depth tracking analysis metrics [23]. In general, tracking applications provide only locomotion related metrics, such as speed or total move length. Some studies built up custom scripts for specific behavior quantifying, e.g., social interaction networks [24], social attraction [25], zigzag walking pattern [26] and centrophobism behavior [27, 28]. Most custom scripts were publicly unavailable or only available-upon-request [24–28]. An interesting work, JAABA, supports users in encoding their intuition about behavior by annotating a small set of video frames and training the classifier in an interactive machine learning manner [29]. The behavior from JAABA was usually defined for a particular purpose and classifier’s performance was restricted by the annotation.

Though fly behavior might be tested for diverse experimental purposes, we have found significant inconsistencies in common fly behavior metrics. For example, Selkrig et al. reported that the average speed of Canton-S flies to be 0.7–1.5 mm/s [11, 28], Schneider et al. found it to be 2–3 mm/s [24] and Martin et al. reported 8–10 mm/s [26]. Apart from the above-mentioned inadequacy in fly behaviors analysis algorithms, the inconsistencies might be brought by different chamber hardware and experimental conditions, given that the chamber design varies across paradigms, in which some used square chambers (from W10 mm*D10 mm*H1.6 mm [7] to W40 mm*D40 mm*H3.5 mm [26]), while others used circular chambers (from Ø12.7 mm [30] to Ø245 mm [8]). The fly density in the chamber, recorded video length and experimental time also varied. Recent researches have shown the chamber size and fly density had a strong effect on fly social network topology [31] and social distances [32].

To elucidate how chamber affects fly behaviors as well as how fly behaves in the chamber, we established a new analysis pipeline with total of 74 distinct metrics, including 49 newly proposed metrics and 25 published metrics focusing on adult fly locomotion and social behaviors in the present study. A software, *Drosophila* Video Tracking (DVT), was developed to implement the pipeline. We further investigated fly behaviors under different the chamber height, fly density, illumination and experimental time by DVT. We also analyzed behaviors of three common-used fly strains Canton-S(CS), *w*^*1118*^ and Oregon-R (OR) and established fly spatiotemporal behavior patterns in the chamber.

## Results

### Drosophila video tracking with 74 locomotion and social behavior metrics.

**Fig. 1:**
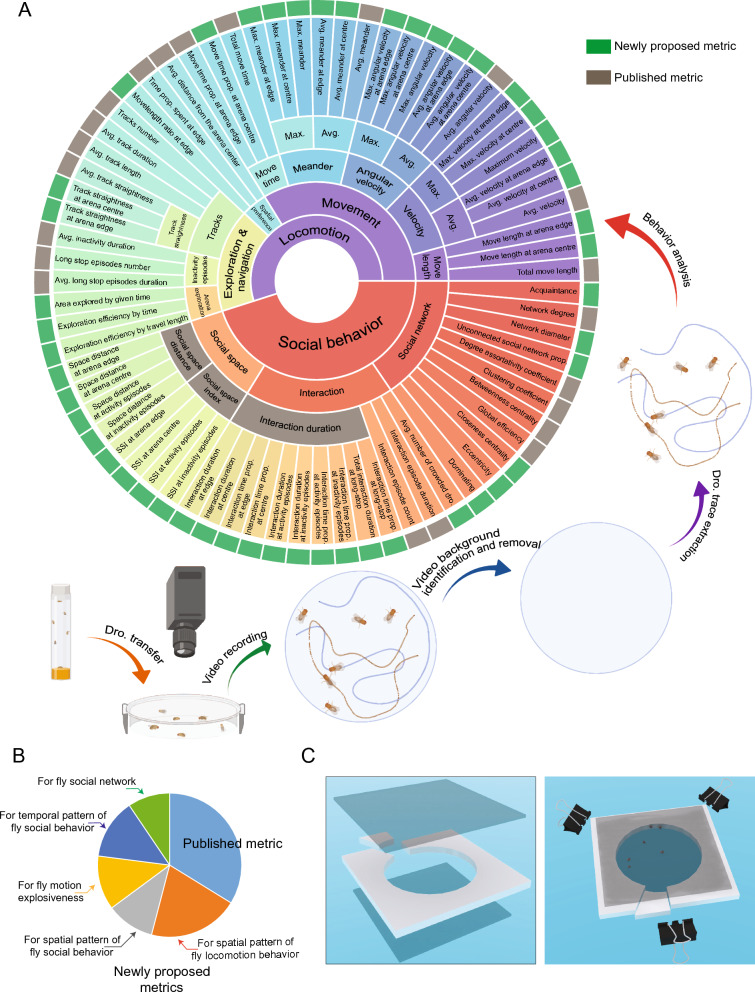
Overview of DVT paradigm. **A** Schematic Diagram on the proposed fly behavior metrics set. **B** Newly proposed metrics distribution. **C** Chamber design in DVT. Dro. abbreviated for drosophila, similarly hereinafter

To compensate for the inadequacy in defining and quantifying fly behaviors, we established a software, *Drosophila* Video Tracking (DVT), with 74 metrics to help build a panoramic understanding of fly locomotion and social behavior. Locomotion related metrics were composed with movement length, velocity, angular velocity, meander, track straightness, move time, spatial preference, and chamber exploration efficiency. The social behavior includes social space, social interaction, and network topology related features (Fig. 1A). The definition of each fly behavior metric was listed in Additional file 1: S1.

49 of the 74 metrics in DVT pipeline were newly proposed which could be divided into five groups (Fig. 1B; Additional file 2: S2). The first group refers to 9 metrics for characterizing fly motion explosiveness, e.g., maximum velocity and maximum angular velocity, etc. In this way we can identify subtle differences between fly motion explosiveness and exercise endurance. Motion explosiveness and exercise endurance are two commonly used phrases in athletics. Explosiveness refers to the neuromuscular system’s ability to generate high action velocities. Peak velocity, i.e. maximum velocity was one of measures for motion explosiveness [33]. Endurance can be defined as the capacity to maintain one’s velocity or power output for the longest possible time [34, 35], thus the average velocity was used to describe endurance. The second group refers to 15 metrics for characterizing spatial pattern of fly locomotion behavior, including average velocity at arena edge and average velocity at centre, etc. The third group refers to 8 metrics for characterizing spatial pattern of fly social behavior, including space distance at arena edge and space distance at arena centre, etc. Metrics in the second group and third group were designed to allows spatial pattern identification for flies when they were located at the arena edge or the arena centre, given the centrophobism of flies. The fourth group refers to 10 metrics for characterizing temporal pattern of fly social behavior, such as interaction duration at activity episodes and interaction duration at inactivity episodes etc. This group of metrics were calculated in case that there might be social behavior characteristic differences between moving status and immobile status of a fly. The last group was a supplement for characterizing fly social network topology, e.g., network diameter and unconnected social network proportion, etc.

Most current tracking software, e.g., Ctrax [8], ilastrk [18], UMATracker [15] support the workflow from video acquisition, background subtraction to fly detection (Fig. 1A). The DVT pipeline takes fly trajectories generated from the above software as its input for behavior analysis. To mitigate the known corner preference of flies [36], DVT supports analysis for flies in circular chambers instead of triangle or rectangle chambers by default (Fig. 1C).

### New features allowing users to better understand fly social behaviors

Most newly proposed metrics are easily comprehensible, such as average velocity at arena edge. In this section we would like to expound on two newly metrics, *acquaintance* and *average number of crowded Drosophila*, which are expected to greatly contribute to our understanding of fly social behaviors.

**Fig. 2:**
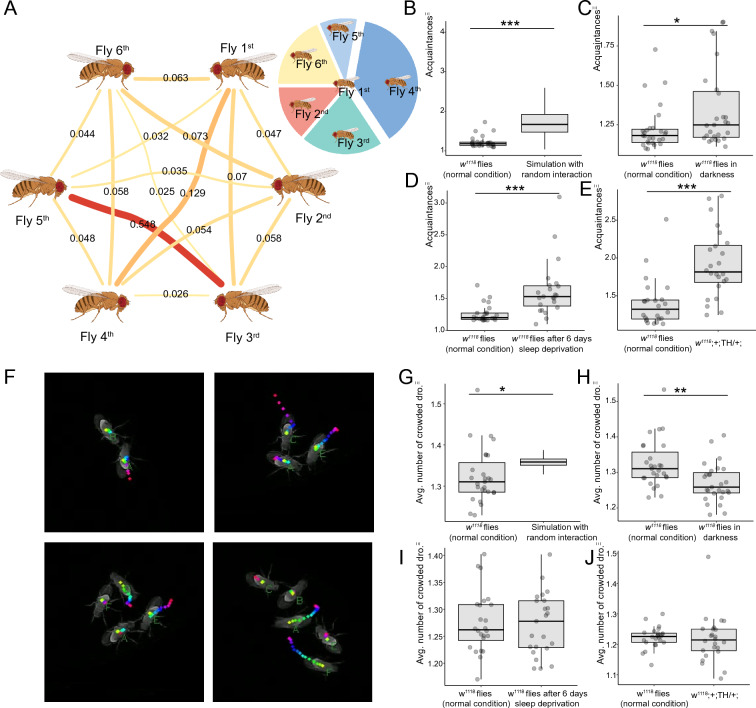
Biased and crowded drosophila social interaction. **A** representative drosophila social network. The right-up panel showed interactions with 4th fly occupied a larger proportion in 1st fly’s interaction pie. **B** Comparison of acquaintance of simulated random social network with that of male *w*^*1118*^ flies under normal condition. **C** Acquaintance was higher for flies in red dim darkness compared with that in normal lighting. **D** Acquaintance was higher for flies after 6 days sleep deprivation. **E** *w*^*1118*^; +;TH/+; flies had higher acquaintance than *w*^*1118*^ flies. **F** Crowded drosophila in an interaction/encounter event. **G** Comparison of avg. number of crowded dro. of simulated random social network with that of male *w*^*1118*^ flies under normal condition. **H** Red dim darkness lowered avg. number of crowded dro. **I** Avg. number of crowded dro. did not change after 6 days sleep deprivation. **J** no significant changes were found for *w*^*1118*^; + ;TH/ + ; male flies compared with *w*^*1118*^ male flies. Statistical analysis: Welch Two Sample t-test was used for comparisons between two groups. *P < 0.05, **P < 0.01, ***P < 0.001. Non-significant test was not annotated in the graph for brevity. For subfigure **B**, **G**, 27 videos for male *w*^*1118*^ flies under normal condition were recorded and analyzed. For subfigure **C** and **H**, 27 videos for male *w*^*1118*^ flies in darkness and 27 videos for male *w*^*1118*^ flies under normal lighting were recorded and analyzed. For subfigure **D** and **I**, 23 videos for male *w*^*1118*^ flies after 6 days sleep deprivation and 24 videos for age matched male *w*^*1118*^ flies without sleep deprivation were recorded and analyzed. For subfigure **E** and **J**, 24 videos for male *w*^*1118*^; + ;TH/ + ; flies and 24 videos for age matched male *w*^*1118*^ flies were recorded and analyzed. All videos were from three independent experiment replicates. The color-coded track in 2C indicates the fly traces in last 15 video frames (0.5 s). The average-by-video value of features were plotted in subfigure **B**–**E**, **G–J**, similarly hereinafter. We annotated newly proposed metrics with the superscript Ξ/ksi/ for subfigures B–J, similarly hereinafter

We found that individual flies showed preferences in their social interactions. Figure 2A presented a representative *Drosophila* social network in which the edge width denoted interaction time. It is observed that most of the social encounters of the 1st fly took place with the 4th fly. This phenomenon inspired us to quantify social preference as the weighted ratio of the maximum interaction duration with other flies to the total interaction duration, as shown in formula (1). We denote this metric as acquaintance.

1 $$\mathrm{Acquaintance}= \frac{\mathrm{The\,max\,interaction\,duration\,with\,other\,flies}}{\mathrm{Total\,interaction\,duration\,with\,other\,flies}}/\frac{1}{\mathrm{number\,of\,flies\,in\,arena}-1}.$$

As an example, the acquaintance of the 1st fly in Fig. 2A could be calculated as below.

2 $$\mathrm{Acquaintance}=\frac{\mathrm{max}\left(0.063, \mathrm{0.032,0.129,0.07,0.047}\right)}{\mathrm{sum}\left(0.063, \mathrm{0.032,0.129,0.07,0.047}\right)}/\frac{1}{6-1}=1.891.$$

Individuals with high acquaintance values are more likely to interact with familiar individuals, not strange ones. A large acquaintance value implies an uneven social activity, with an individual fly with high acquaintance preferring to interact with familiar ones. But rather, fly social interactions are more evenly distributed when acquaintance values are lower. To demonstrate the performance of this metric, we conducted Monte-Carlo simulations for building the acquaintance benchmark of random social interaction network (Fig. 2B). It turned out male *w*^*1118*^ flies maintained a more open and even social network with lower acquaintance index compared to randomly simulated networks. To investigate how fly acquaintance changes confronted with different environmental or genetic variations, we further compared acquaintance of male *w*^*1118*^ flies under normal condition, flies in red dim darkness, flies after 6 days sleep deprivation and *w*^*1118*^;+;TH/+; flies. Both illumination deprivation (Fig. 2C) and sleep deprivation (Fig. 2D) would increase the fly acquaintance. The mutant allele in* w*^*1118*^; +; TH/+; codes tyrosine hydroxylase and affects L-DOPA and dopamine biosynthesis [37, 38]. Compared with male *w*^*1118*^ flies, male *w*^*1118*^; +; TH/+; flies showed a higher acquaintance (Fig. 2E), suggesting the mutant flies spent more time with familiar ones.

Another phenotype we observed was the preferential association of three or more flies in a single encounter/interaction event (Fig. 2F). We quantified this phenotype using average number of crowded Drosophila, which measures the average of the number of flies that interact with the same fly simultaneously. A simplified scenario was used to expound the calculation of this metric as following. The fly A interacts with fly B and fly C at the same time. In this case, the number of crowded *Drosophila* is 2 for fly A. Then all interaction events of fly A were traversed to get the average value of the metric in the recorded video. If no crowding occurred, the metric would be equal to 1. A lower average number of crowded Drosophila might be caused by social avoidance, competing attractor existence, or the lack of awareness of another fly, etc. Male *w*^*1118*^ flies had a lower average number of crowded drosophila than that from simulated random network (Fig. 2G). Red dim darkness would decrease the average number of crowded Drosophila, suggesting the metric has an illumination dependence (Fig. 2H). Though we observed sleep deprivation changed fly acquaintance, but the average number of crowded Drosophila was not changed after sleep deprivation (Fig. 2I). Comparably, no significant change was observed for the average number of crowded dro. of male *w*^*1118*^; + ;TH/+; flies (Fig. 2J).

### Using average-by-video features to mitigate reID error effect

Each fly detected in one frame is associated with an identity from the former frame in trajectories generation. If flies are assigned to the wrong identity due to occlusion or crossing of fly bodies, re-identification (reID) errors occur [23, 39]. reID errors correction is another limitation in large-scale applications of high throughput fly behavior analysis. Though Ctrax [8], UMATracker [15] and some other trace detection suits provided manual correction helper software, we found that it would take at least twice time as long as the length of the video to correct reID errors. This job was quite labor-intensive.

There are three ways to reduce reID error rate. Firstly, clip the wings of the flies (Ctrax [8], Flytracker [40], Buridan’s Paradigm [10]) and coat the celling glass with Fluon or Sigmacote (Jiang et al. [41], Flytracker [40], Dankert et al. [42]) to prevent flies from walking on the ceiling and obscuring the imaging of other flies to reduce reID error. These operations lead to inaccuracy and bias in the behavior quantification. The second way is using chambers with sloped walls to reduce fly body occlusion at the wall (Simon et al [43], Flytracker [40]). The main disadvantage of sloped walls was that it would change fly spatial preference and centrophobism behaviors [43]. Idtracker [39], ToxTrac [14] and Idtracker.ai [22] tried the third way, attempting to preserve fly identification across frames by Bayesian analysis, Hungarian algorithm or deep neural network instead of hardware modifications. However, none of these paradigms can perfectly identify each fly through the video due to potential error propagation [22, 40].

Since reID error is inevitable during trajectories generation, we have to take reID error into account and eliminate bias in behavior analysis brought by reID error. We proved that the average-by-video behavior before correction is equal to or expectedly equal to that after correction given the assumption that chamber is in a homogeneous setting, i.e., the flies in one chamber are composed of individuals from the same strain or genotype, or reared from the same environment. Detailed proof was presented in the Additional file 3: S3.

Hence, we established the new analysis pipeline for fly behavior analysis in DVT. In traditional analysis pipeline, researchers have to correct the ReID error manually after trajectories generation and the behavior metrics were calculated for each individual fly in the chamber. The analysis result, composed of behavior metrics for individual flies in serval videos, was piped into statistical analysis (Additional file 8: Fig. S1A). In the newly proposed pipeline, behavior metrics were directly calculated for each individual fly without ReID error correction. Then, take the average of behavior metrics of flies in one video. Since we have proved these average-by-video features was unbiased even if ReID error was not corrected, the averaged features of each video instead of individual flies were used in following statistical analysis (Additional file 8: Fig. S1B). For example, if we got 6 flies in the chamber, the average velocity of the 6 flies was used instead of 6 biased individual velocity.

To validate the new analysis pipeline, we manually corrected reID errors of 12 videos each lasting 30 min (Additional file 4: S4). The reID error rate varied for different genotypes and decreased with a reduced move time. This is consistent with the expectation that locomotion increases probability of flies encountering each other and crossing paths, increasing the occurrence of reID errors. No reID error was found in behavior videos of Canton-S (CS) flies because its interaction/encounter event counts were lowest among the flies tested. Oregon-R (OR) flies were less likely to cross each other leading to a lower reID error rate than that of *w*^*1118*^ flies. The average-by-video value of fly behavior features before correction matched the value after correction, with a Pearson correlation median > 0.95 (Additional file 8: Fig. S1C). It is important to note that this validation was conducted under the assumption of homogeneity within the chamber. If users are interested in individual fly behavior in heterogeneous chamber, for example, if male flies and female flies were put into one chamber for obversion of courtship, manual reID error correction is still needed (Additional file 8: Fig. S1D).

### Chamber design and experimental condition affect fly behavior

In this section, we further investigated the spatial and temporal pattern fly behavior confronted with different chamber hardware and experimental conditions.

#### Chamber height changed fly behavior spatial-specifically

Previous paradigms used chambers with heights that ranged from 1.6 mm to 3.5 mm (Additional file 5: S5). To determine how chamber height affects fly behavior, we built two kinds of chambers, one with 2 mm height, the other one with 3 mm height. Chambers with higher heights were not used because parallax bias might be introduced to the calculation due to camera hardware limitations.

**Fig. 3:**
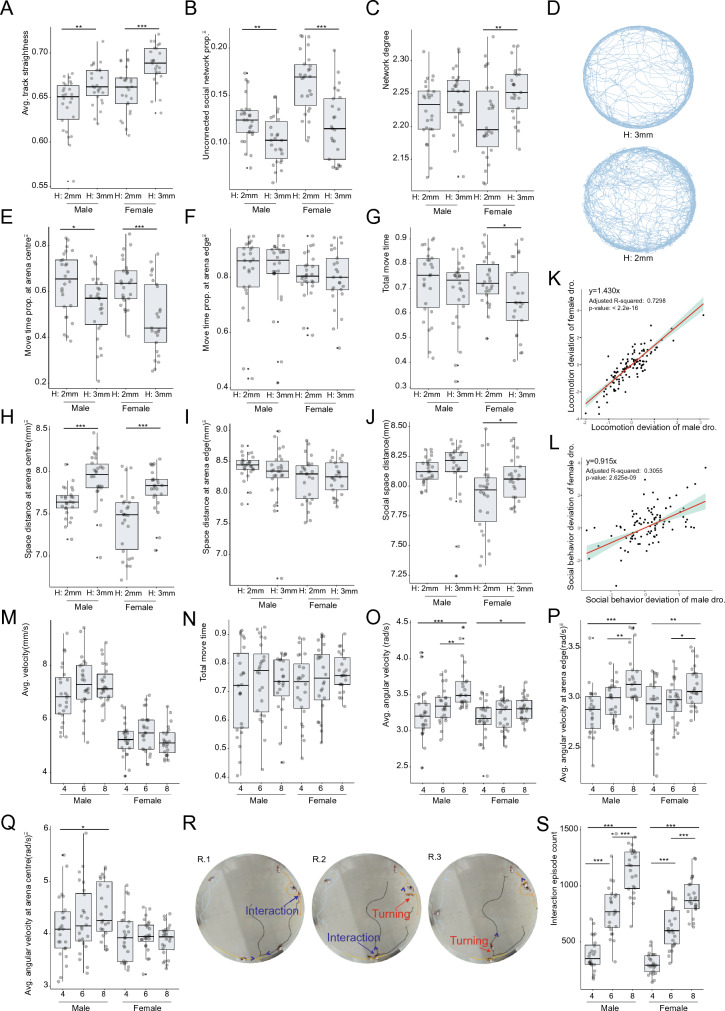
Chamber height and fly density affect behavior. **A** Track straightness was higher at 3 mm-height chamber. **B** Unconnected social network prop. was lower at 3 mm-height chamber. **C** Network degree was higher at 3 mm-height chamber. **D** Representative motion trace for female flies in 2 mm-height and 3 mm-height chamber. **E** Move time prop. at arena centre was lower at 3 mm-height chamber. **F** Move time prop. at arena edge showed no difference between 3 mm-height or 2 mm-height chambers. **G** No significant change was observed for males in 2 mm-height chamber given the metric total move time. **H**, Space distance at arena centre was larger in 2 mm-height chamber. **I**, Space distance at arena edge was not affected by chamber height. **J** Female flies showed changed social space distance in the 2 mm-height chamber. **K** locomotion behavior deviation of female flies was about 1.4 times that of male flies in 2 mm-height chamber. **L**, social behavior deviation of female flies was roughly same with that of male flies in 2 mm-height chamber. **M** No significant change on fly speed was identified with different density (4, 6, or 8 flies) in the chamber. **N** No significant change on fly move time was identified with different density in the chamber. **O** Angular velocity changed with different density. **P** The changes of angular velocity happened at arena edge. **Q** Only a little change of angular velocity was found at arena centre with different density. **R** fly turning was associated with interaction events. S, Interaction episode count significantly increased with higher fly density. Statistical analysis: Welch Two Sample t-test was used for comparisons between two groups. *P < 0.05, **P < 0.01, ***P < 0.001. Non-significant test was not annotated in the graph for brevity. A modified Cohen’s d was used to measure standardized effect size. The effect size for female flies and male flies was linear regressed to assess the spatial restriction on flies at **K** and **L**. For experiment on behavior changes on chamber height, n = 26 videos for male *w*^*1118*^ flies in 2 mm-height chambers, n = 26 videos for female *w*^*1118*^ flies in 2 mm-height chambers, n = 26 videos for male *w*^*1118*^ flies in 3 mm-height chambers, n = 25 videos for female *w*^*1118*^ flies in 3 mm-height chambers were recorded and analyzed. For experiment on behavior changes on fly density, n = 24 videos for male *w*^*1118*^ flies in 4-flies chambers, n = 24 videos for male *w*^*1118*^ flies in 6-flies chambers, n = 23 videos for male *w*^*1118*^ flies in 8-flies chambers, n = 24 videos for female *w*^*1118*^ flies in 4-flies chambers, n = 24 videos for female *w*^*1118*^ flies in 6-flies chambers, n = 24 videos for female *w*^*1118*^ flies in 8-flies chambers were recorded and analyzed. All videos recorded were from three independent experiment replicates

We calculated the effect size to illustrate the behavior feature deviation of flies in 2 mm-height chamber from that in 3 mm-height chamber (see “Methods” for calculation details). Radar plots were drawn to show the overall view of locomotion (Additional file 8: Fig. S2A) and social behavior (Additional file 8: Fig. S2B) changes by effect size. An obvious change on locomotion for flies in 2 mm-height chamber was the decrease in trace straightness (Fig. 3A). This finding could be validated by the representative motion traces of female flies in 2 mm-height and 3 mm-height chamber (Fig. 3D). In addition to the locomotion behavior changes in 2 mm-height chamber, social behavior differences were also observed. With respect to the social network topology, the unconnected social network proportion increased (Fig. 3B). In the connected part, decreased network degree was observed (Fig. 3C). Combined, these data suggest that compared with flies in 3 mm-height chambers, the social network of flies in 2 mm-height chambers tended to be smaller and unconnected, in which there were fewer number of hub nodes flies and most flies only interacted with a limited number of other flies.

Using newly proposed metrics, we further found that fly behavior changed spatial-specifically in 2 mm-height chambers. For example, both male and female flies moved much longer at the arena centre of 2 mm-height chambers (Fig. 3E). On the contrary, no statistically significant difference on move time existed at the arena edge (Fig. 3F). This subtle behavior difference would be masked if researchers only got the overall move time metric for male flies (Fig. 3G). Besides the locomotion behavior, this spatial-specifically changing pattern confronted with different chamber heights could also be observed in fly social behaviors. The social distance was shorter only in the arena centre of 2 mm-height chambers, indicating flies stayed more closely from each other (Fig. 3H, I). However, the overall social distance of male flies showed no significant change between different chambers (Fig. 3J). In conclusion, the newly proposed metrics facilitate the investigation on spatial characteristics of fly behaviors.

From above behavior comparisons, the chamber height was likely to affect female flies more than males (Fig. 3A–J). Considering that the body size of females is larger than that of males, we inferred behavior changes in 2 mm-height chambers were likely to be due to spatial restriction. Furthermore, we compared the effect size between female flies and male flies and found that the deviation in the locomotion of female flies in the 2 mm-height chamber was 1.4 times larger than that of males (Fig. 3K). In contrast, the difference of social behavior between male and female flies was not so big as that of locomotion behavior (Fig. 3L).

#### Fly swerved more frequently in high density chambers

It has been reported that chamber size and fly density could significantly alter fly social network topology [31]. Hence, we aimed to identify how fly locomotion changes with density. The experiment was designed to transfer 4, 6 or 8 flies in the chamber and record their behaviors. Since it has been found 2 mm-height chamber would bring spatial restrictions to fly locomotion, all chambers were set to 3 mm-height.

The overall view of locomotion and social behavior changes by effect size for flies with different density profiles was illustrated in Additional file 8: Fig. S3. The change in fly density had no effect on speed or move time, though flies moved a little but no statistically significant faster with a density of 6-flies per chamber compared to 4-flies (Fig. 3M, N; Additional file 8: Fig. S3A, C). In contrast, the angular velocity increased with fly density (Fig. 3O), especially in arena edges (Fig. 3P, Q). We investigated the basis of this effect by re-examining the fly traces and discovered that there was a high probability of swerving upon encountering another fly (Fig. 3R). Since the number of interactions increased with more flies were placed in the chamber (Fig. 3S), it is likely that the increased angular velocity was caused by more turnings. Meanwhile, notable deviations were observed for nearly all social behavior metrics (Additional file 8: Fig. S3B, D). This suggested that the number of flies in the chamber should be consistent throughout all of experiments.

#### Hesitation and adaptation in darkness

Illumination was another one of varied experimental conditions in current behavior recording paradigms. For example, Ctrax [8], Simon’s work [43] used infrared light as backlighting sources. Flytracker [40] records fly activities in complete darkness to avoid interference from visible light. To test the possible effects of decreased visual cues in darkness on fly behaviors, we studied fly behaviors in darkness (dim red) and normal illuminations.

**Fig. 4:**
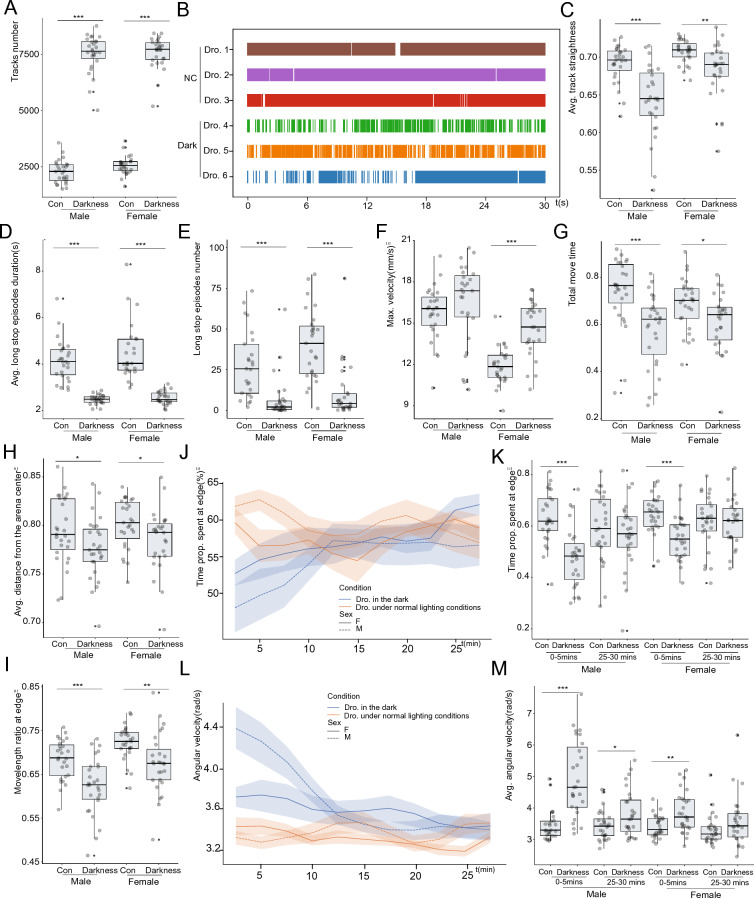
Fly locomotion changes in darkness. **A** Tracks number increased about 3 time higher in darkness. **B** representative track event plot for drosophila in darkness or under normal lighting. **C** Track straightness decreased in darkness. **D** Long stop episodes duration decreased in darkness. **E** Long stop episodes numbers decreased in darkness. **F** Max. velocity increased in darkness. **G** Move time decreased in darkness. **H** Avg. distance from the arena center decreased in darkness. **I** Movelength ratio at edge decreased in darkness. **J**, **L** The time proportion spent at the edge (**J**) and angular velocity (**L**) were changing over the videotaped 30 min for drosophila in darkness or under normal lighting. **K** Difference of time prop. spent at edge of flies under normal illumination and darkness was eliminated at end of the video. **M** Difference of avg. angular velocity of flies under normal illumination and darkness was eliminated at end of the video. Statistical analysis: Welch Two Sample t-test was used for comparisons between two groups. *P < 0.05, **P < 0.01, ***P < 0.001. Non-significant test was not annotated in the graph for brevity. n = 26 videos for male *w*^*1118*^ flies under normal illumination, n = 27 videos for female *w*^*1118*^ flies under normal illumination, n = 27 videos for male *w*^*1118*^ flies in darkness, n = 27 videos for female *w*^*1118*^ flies in darkness were recorded and analyzed. All videos recorded were from three independent experiment replicates

Red dim darkness greatly affected the entire fly behaviors (Additional file 8: Fig. S4). A remarkable change was that the tracks number increased significantly in darkness (Fig. 4A, Additional file 8: Fig. S4A). A track is defined as the path treaded by a fly when it moves continuously. This concept is inherited from Aggarwal’s work [44]. The movement event plot validated our inference that there were more stops and runs in darkness compared with normal illumination (Fig. 4B). Meanwhile, the track straightness was decreased in darkness (Fig. 4C). Flies were inclined to reduce frequency and duration of the longer-than-2 s immobile inactivity (Fig. 4D, E). We also observed an increase in maximum speed and decrease in move time, implying that flies travel more quickly through the arena in darkness (Fig. 4F, G). However, no significant changes were detected for the average velocity (Additional file 8: Fig. S4A). All these clues suggested that flies in darkness would be more watchful and hesitate to take a long running or long stops. Flies would prefer to quickly pass through short and bended tracks, stop for a tiny little while and run again.

Besides, our data provided evidences that centrophobism behavior had a dependency on illumination. Both the average distance from arena center (WAFO) and move length ratio at the arena edge decreased in the darkness (Fig. 4H, I). Flies might need vision cues to identify arena edges. A fascinating discovery from temporal pattern analysis is that some metrics suggest that flies were adapting to the darkness. For example, it took about 10 min for the metric *time spent at arena edge* to begin approaching levels in illuminated conditions (Fig. 4J). The statistical test showed that flies had a much lower *time proportion spent at arena edge* at the first 5 mins of recorded video. While at the 25–30 mins of the video, there was no difference between flies in darkness and normal illuminations (Fig. 4K). Flies might have memorized the arena edges or got used to the darkness. Other behavior metrics, such as angular velocity, also supported our conclusion about the adaption in the darkness (Fig. 4L, M).

Consistent with the findings from Burg et al. [45], social behaviors also changed in darkness (Additional file 8: Fig. S4B). The social interactions and the social space index were reduced in darkness. The network degree, cluster coefficient and assortativity were decreased in darkness. Taken together, these data showed that fly behavior was significantly affected by illumination.

#### Fly locomotion and social behavior follow circadian rhythms

Fly behavior is governed by circadian clocks [46]. Contributed by advancement of drosophila activity monitoring device, studies have revealed flies exhibit peaks of activity during dawn and dusk [47]. To examine the rhythmic changing pattern of each behavior, we conducted a circadian behavior experiment of male flies from ZT00 to ZT11 quaque hora.

**Fig. 5:**
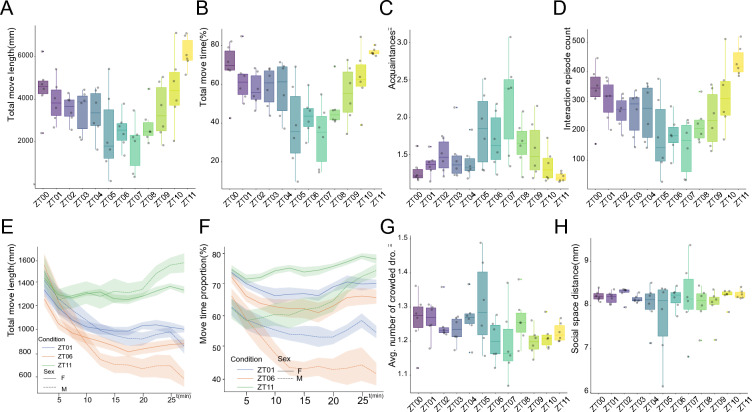
Circadian rhythm in fly behavior. **A**–**D**, move length (**A**), move time (**B**), acquaintances (**C**), interaction episode count (**D**) showed a circadian rhythm from ZT0 to ZT11. **E** Difference of total move length emerged at about 10 min after recording start. **F** Difference of move time emerged at about 10 min after recording start. **G**, **H** Avg. number of crowded dro. **G** and social space distance (**H**) did not follow circadian rhythm. Statistical analysis: n = 6 videos for male *w*^*1118*^ flies for each hour from ZT0 to ZT11 (**A**–**D**, **G**, **H**). For detailed comparison of behavior at ZT01, ZT06 and ZT11(E, F), n = 6 videos for male *w*^*1118*^ flies at ZT01, n = 6 videos for female *w*^*1118*^ flies at ZT01, n = 6 videos for male *w*^*1118*^ flies at ZT06, n = 6 videos for female *w*^*1118*^ flies at ZT06, n = 6 videos for male *w*^*1118*^ flies at ZT11, n = 6 videos for female *w*^*1118*^ flies at ZT11 were recorded and analyzed

From the perspective of locomotion, the move length and move time were lowest at ZT05 to ZT07 which is consistent to existing studies on fly sleeping rhythms (Fig. 5A, B). With respect to social behavior, fly also showed a rhythmic changing pattern. Flies at ZT05 to ZT07 had a higher acquaintance and lower interaction than flies at ZT00 or ZT11 (Fig. 5C, D). Furthermore, we investigated fly behaviors recorded at ZT01, ZT06 and ZT 11 (Additional file 8: Fig. S5). The temporal behavior pattern revealed that the move length (Fig. 5E) and move time (Fig. 5F) were similar in the first 5–10 min at the three time points. Dramatic differences emerged at the last 20 min of recorded videos, suggesting that the first 5 or 10 min might be an acclimation phases for flies in the chamber.

Some metrics were robust confronted with time points changes. As shown in Fig. 5G, H, the social distance and the newly proposed metric *avg. number of crowded flies* did not show rhythmic variations.

### Different strains of *Drosophila* showed different behavior patterns

Though *Drosophila* has received a lot of attention in behavioral studies, a comprehensive investigation on the behaviors of different strains has not been carried out. In this section, the behaviors of frequently used control lines, Canton-S(CS), *w*^*1118*^ and Oregon-R (OR) were surveyed to explore how fly behaves in the chamber.

#### Correlation network reveals innate relationships between behavior metrics.

**Fig. 6:**
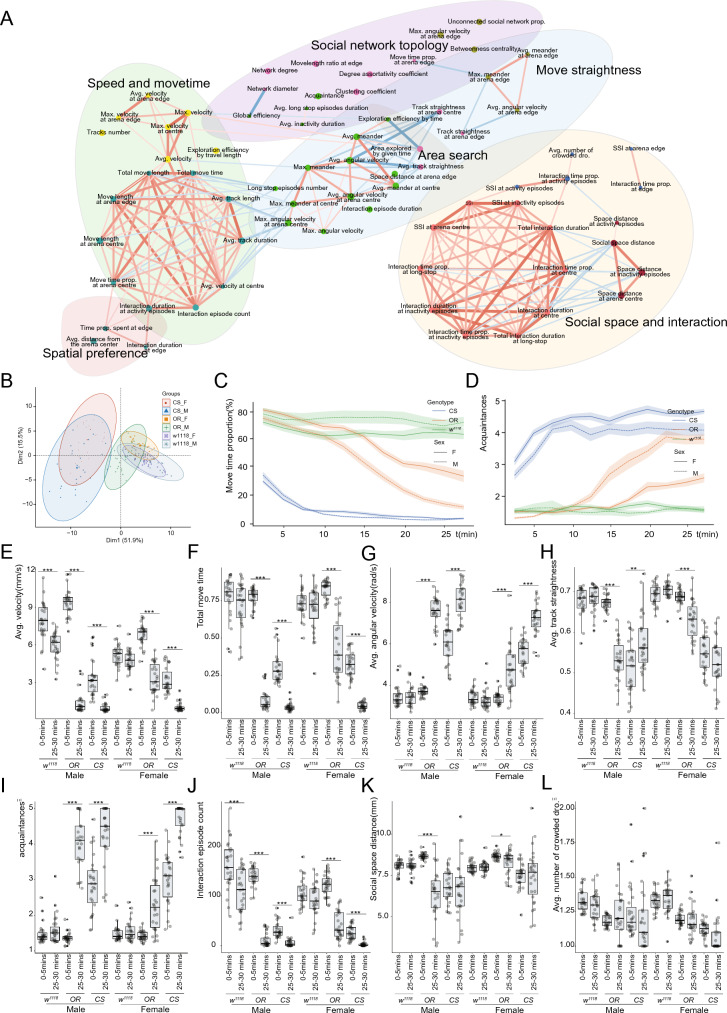
Drosophila behavior patterns. **A** Drosophila behavior feature correlation network. B, PCA plot for drosophila behavior of different strains. **C**–**D** Move time proportion (**C**), acquaintance (**D**) of drosophila showed a different changing pattern over the videotaped timeline. **E**–**L** Speed (**E**), move time (**F**), angular velocity (**G**), track straightness (**H**), acquaintance (**I**), interaction episode count (**J**), social space distance (**L**) changing patterns of flies in the chamber. Statistical analysis: Welch Two Sample t-test was used for comparisons between two groups. *P < 0.05, **P < 0.01, ***P < 0.001. Non-significant test was not annotated in the graph for brevity. n = 27 videos for male *w*^*1118*^ flies, n = 27 videos for female *w*^*1118*^ flies, n = 26 videos for male OR flies, n = 27 videos for female OR flies, n = 27 videos for male CS flies, n = 27 videos for female CS flies were recorded and analyzed. All videos recorded were from three independent experiment replicates. Multi-level correlation of paired behavior metrics was calculated to build the network. The network plot drawn metric relationships with magnitude of correlation larger than 0.7 and FDR q-value < 0.05. The edge width is proportional to correlation magnitude. And a red edge is sign of positive correlation and green edge is for negative correlations. The node color represents cluster identification of metric made by kmeans cluster algorithm

Based on the behavior data obtained using CS, OR and *w*^*1118*^, we generated a multi-level correlation [48] network to visualize the potential relationships between metrics (Fig. 6A; Additional file 8: Fig. S6). The metrics were clustered into six groups in the network plot. Across all three strains, several social behavior metrics had strong correlations with locomotion metrics. For example, the interaction episodes count correlated with fly move time. Social network topology related metric, like clustering coefficient, was classified into the same cluster with fly track straightness by K-Means algorithm. The social space distance and social space index showed correlation with fly interaction related metrics. The strong correlations suggested the locomotion defects should be carefully considered when exploring fly social behaviors.

Specifically, we checked the two newly proposed metrics. Acquaintance has a positive correlation with fly angular velocity and interaction duration and a negative correlation with fly moving velocity, clustering coefficient and social network degree. The correlation analysis implied that high acquaintance was often connected with rectilinear-movement reduction and social network efficiency degradation from the perspective of group behavior. Unlike acquaintance, the average number of crowded Drosophila showed no correlation with locomotion metrics. For this metric, the most relevant feature was interaction time prop. at activity episodes. In fact, most correlated metrics of the average number of crowded Drosophila were interaction-related.

#### Temporal behavior pattern differs between strains with different genetic backgrounds.

Principal component analysis (PCA) revealed significant pattern difference between strains. OR flies were more similar to *w*^*1118*^, probably because the *w*^*1118*^ mutation originated from an Oregon-R background [49] (Fig. 6B). To illustrate the behavioral changes across the video timeline, we presented the temporal pattern plots for move time proportion (Fig. 6C) and acquaintance (Fig. 6D).

In these three stains, *w*^*1118*^ had better motion explosiveness and exercise endurance. OR flies had similar motion explosiveness with *w*^*1118*^ but the locomotion capability of OR flies degraded rapidly at about 10 min. The locomotion capability of CS flies was the worst. In fact, CS flies began to settle after an initial period of vigorous walking lasting approximately 5–10 min. Studies from Brug et al. and Xiao et al. supported this finding [45, 50]. To further verify this phenomenon, we compared behavior during the first 5 min of the recorded videos to the last 5 min. More significant feature changes were detected in OR and CS flies than in *w*^*1118*^ flies in the comparison (Additional file 8: Fig. S7). A downward tendency in the fly locomotion capability over time was identified, which was consistent for all three strains (Fig. 6E; Additional file 8: Fig. S7A, S7C, S7E). *w*^*1118*^ flies could maintain the move time, angular velocity and track straightness during the 30 min recording while OR and CS flies downgraded severely (Fig. 6F–H).

Social behavior temporal pattern also changed, with increased acquaintance and decreased interaction, suggesting that flies tended to communicate with familiar flies at the last part of video recording (Fig. 6I, J, Additional file 8: Fig. S7B, S7D, S7F). An interesting finding is that the social distance and the newly proposed metric *avg. number of crowded flies* were robust to time spent at the chamber, except for OR flies (Fig. 6K, L). These two metrics were also insensitive to circadian rhythms. A possible reason is the metric *avg. number of crowded flies* had no correlation with locomotion metrics while the social distance had weak correlation with angular velocity related metrics instead of speed related metrics (Additional file 8: Fig. S6).

#### Spatial behavior pattern differs between arena edge and centre.

**Fig. 7:**
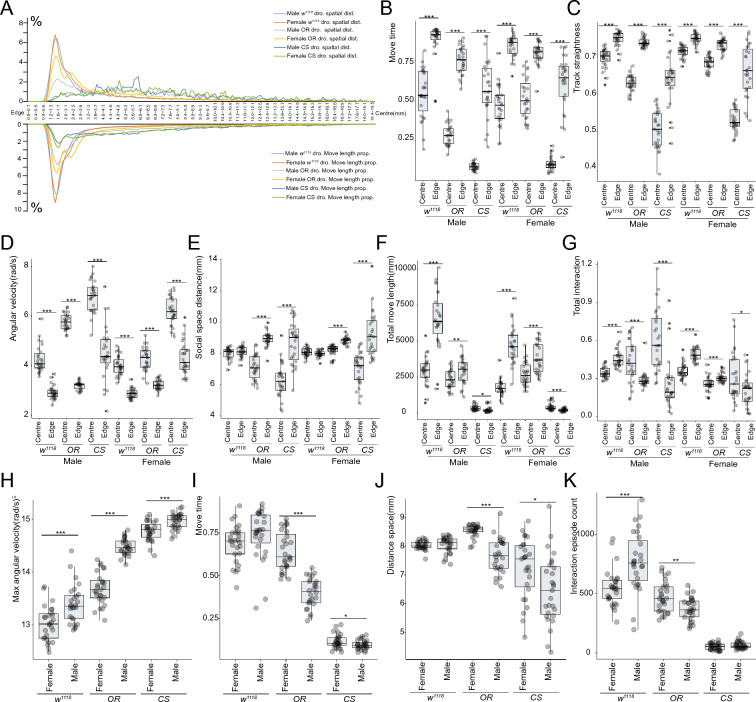
Behavior spatial pattern of flies. **A** Drosophila spatial distribution and move length distribution along the arena edge-centre axis. **B–G** Spatial patterns of move time (**B**), track straightness (**C**), angular velocity (**D**), social space distance (**E**), total move length (**F**), total interaction (**G**) for flies. **H**–**K** Male and female fly behavior difference for max angular velocity (**H**), move time (**I**), distance space (**J**), and interaction episode count (**K**). Statistical analysis: Welch Two Sample t-test was used for comparisons between two groups. *P < 0.05, **P < 0.01, ***P < 0.001. Non-significant test was not annotated in the graph for brevity. n = 27 videos for male *w*^*1118*^ flies, n = 27 videos for female *w*^*1118*^ flies, n = 26 videos for male OR flies, n = 27 videos for female OR flies, n = 27 videos for male CS flies, n = 27 videos for female CS flies were recorded and analyzed. All videos recorded were from three independent experiment replicates

We noticed fly behavior was different at arena edge and arena centre, likely due to centrophobism. However, how the boundary edges of the arena were defined diversely in literatures. Bath et al. set the inner 36 mm in a 54 mm circular chamber as the edge boundary [7], while Besson et al. used 20 mm in a 40 mm*40 mm square chamber [51]. Valente suggested defining the arena boundary by marginal radial probability distribution [52]. Thus, to allow clear definitions of “centre” and “edge”, we investigated the spatial preference of the three stains and plotted the spatial position distribution and spatial movement distribution (Fig. 7A). Because the center of fly body was used to represent the fly position, no traces were found at the initial 0.5 mm from the arena edge. Then a peak emerged at 0.6–3 mm from the arena edge. We therefore set 3 mm from the arena edge as the boundary between edge and centre for the 37 mm chamber used in this study.

Though flies have different motion explosiveness and exercise endurance, the speed, move time and track straightness showed similar changing pattern from the arena edge to centre. Most movement of flies occurred at the arena edge (Fig. 7B). The track straightness was higher at the arena edge (Fig. 7C) and fly moved faster at the arena centre (Fig. 7D). The chamber-wall-restricted locomotion at the edge and anxiety in the centre of the arena might account for these differences, as centrophobism is often associated with anxiety-like behavior.

Spatial pattern of other metrics was different from strains. The social space distance increased in the arena edge for OR and CS flies, while no significant difference was found for *w*^*1118*^ flies (Fig. 7E). OR flies and *w*^*1118*^ flies had a higher move length at arena edge (Fig. 7F). The interaction decreased at arena edge for OR and CS flies (Fig. 7G).

#### Behavioral differs between male and female Drosophila

Sex differences have been found for a wide range of fly activities. Compared with female flies, male flies of the three frequently used control lines had faster angular velocity, higher maximum velocity, and lower track straightness identified by DVT (Fig. 7H; Additional file 8: S8A, S8C, S8E). Differences of social behavior also existed between male flies and females (Additional file 8: S8B, S8D, S8F). We also identified several metrics which exhibited strain dependent male–female difference patterns. For example, move time and distance space showed no difference for *w*^*1118*^ flies (Fig. 7I, J), while no male–female difference was found for interaction episode count of CS flies (Fig. 7K).

## Discussion

In this work we presented a new set of metrics on fly locomotion and social behavior and implemented DVT software for trajectory analysis. The behavior set was composed of 74 metrics of which 49 were newly proposed for temporal and spatial pattern characterization of fly behavior in the chamber. In DVT, a new and simplified pipeline using average-by-video features for fly behavior analysis was validated, in which the labor-intensive manual work reID error correction was saved.

As an exquisite and sensitive animal, fly behaviors are affected by genetic variation or environmental factors. In the earlier studies, Valente et al. built up the joint/marginal probability distributions of fly velocity and position [52]. Colomb et al. calculated centrophobism indexes for moving and for sitting independently [10]. Their work suggested that fly showed different locomotion pattern in the rim zone and the central zone [52]. Their works were done for a single fly walking in a circular open field arena, the social behavior pattern in different chamber zone remained unrevealed. Meanwhile, Martin et al. visualized time-course of the movement distance [26]. Ctrax provided ‘Per-Frame Property’ function for behavior exploration by video frames [8]. However, understandings on the temporal dynamics of fly behaviors in the chamber are still limited.

In the present study, impressively, we found fly behavior showed quite different patterns given different chamber and experiment settings. For example, the proportion of unconnected social network increased by 50% for female flies in 2 mm-height chambers than that in 3 mm-height ones. The number of tracks of flies in darkness was about 3 times of that in normal illuminations. Meanwhile, our studies also showed fly locomotion and social behavior followed circadian rhythms. Move time at ZT00 was twice as much as flies at ZT06. These findings suggested we should strictly control the experiment settings for reproducibility and consistency of fly behaviors across studies. There still remains many behavioral phenomena without published interpretation, for example, the hesitant behavior of flies in the dark, the different behavioral characteristics of flies in the arena centre and the edge. Future work is needed in deciphering these behavior features.

Behavior pattern identification of frequently used control lines by DVT also revealed serval interesting conclusions. One highlight is that the three widely used flies exhibited distinct motion explosiveness and exercise endurance, suggesting that the selection of fly stains as experimental controls should be done cautiously. Indeed, it is notable that Canton-S flies became inactive soon after getting into the chamber. Canton-S flies therefore might not be a good choice for locomotion-purpose experiments, such as sleep deprivation, as it would be difficult to distinguish whether the fly is immobile by itself or by experimental conditions.

Investigations on fly behaviors under different chamber hardware and experimental conditions further demonstrated necessity of our newly proposed metrics. With aiding from these metrics, it has been identified that flies exhibited distinct locomotion and social behavioral features temporospatially in the chamber, for example, the track straightness was higher at the arena edge and fly moved faster at the arena centre. The newly proposed metrics also revealed that fly responses to specific treatments were temporospatial dependent. For instance, flies moved much longer only at the arena centre in 2 mm-height chambers. Besides, we also incorporated metrics for characterization fly motion explosiveness, e.g., maximum velocity. An increase in maximum velocity was observed for flies in darkness while there was no significant change in average velocity. All above examples showed performance of newly proposed metrics on revealing subtle behavior changes.

The experiments on *w*^*1118*^; + ;TH/+; flies, sleep deprived flies and other varied hardware conditions have provided evidences for DVT’s capacity to identify and quantify behavioral differences under different conditions or genotypes. With opensource hardware and software, DVT has the flexibility to be modified for specific experimental aims. For example, researchers can replace the floor glass of the chamber by fly food for long period behavior analysis. DVT can also be used to analyze fly responses to light/air-delivered stimulations as well. We provided fly behavior metrics at each video frame as process outputs by default and researchers can perform their analysis on the raw or processed data accordingly. On the other hand, we have observed a deep connection between locomotion and social behavior. Social behavior changes should be rigorously interpreted if flies have a locomotion defect. In this case, additional experiments, e.g., fly social avoidance [53] might be needed for further confirmation.

Recently, pose estimation and body orientation related algorithms and software emerged [19, 21]. It is foreseeable that these algorithms support better descriptions on fly postures. Complex behaviors, including courtship, aggression etc. could be identified by these algorithms. Furthermore, researchers could also be able to gain an insight into behavior subtypes, e.g., head-to-tail interaction or head-to-head interaction. However, currently the false detection rate is less than satisfying with reported erroneous orientation rate at 1/s to 4/s [43]. Hence, we did not integrate pose estimation and body orientation into our analysis pipeline. This leads to a lack of complex behavior analysis capability in DVT. We also noticed the hardware and software for three-dimensional position tracking by multiple synchronized and calibrated cameras is being mature [54]. In the future, we will update DVT aiming three-dimensional positions data and complex behavior analysis. In summary, we believe that our work would be a valuable resource for the quantification of fly behaviors for the research community.

## Methods

### Fly husbandry and internal state manipulations

Flies were reared on the standard medium (per liter of medium: 21.08 g sucrose (Sinopharm Chemical Reagent, Ltd, cat. #10021418), 42.16 g glucose (Sinopharm Chemical Reagent, Ltd, cat. #63005518), 20.79 g yeast powder (Angel), 7.07 g agar (BioFroxx, cat. #1182GR500), 51.8 g cornmeal flour, 0.484 g CaCl2, 1.33 g of potassium sorbate (Aladdin, cat. #P103845)) and maintained at 25 °C, 50–60% relative humidity, 12-h light: dark cycle. Flies were transferred to new vials with fresh medium every two days.

### Fly stocks and genetics

The Oregon-R (BS 5) were obtained from the Bloomington stock center. *w*^*1118*^, Canton-S flies and *pale* flies (w, yw; +; TH/TM3,Sb;) were from Yi Rao Lab. We crossed the *pale* flies (male) with virgin *w*^*1118*^ (female) and obtained male flies *w*^*1118*^;+;TH/+;.

### Fly behavior recording hardware implementation

Chamber with circular Ø37 mm arena was used in fly behavior recording. The ceiling and floor of chambers were made of glasses (50 mm by 50 mm, with a thickness of 1 mm). The main body of chamber were cut from 3 mm or 2 mm thick transparent acrylic sheet.

Two 50 × 70 cm soft-box with 150 W LED photography kits were used as the lighting source in the 3 m*4 m drosophila behavior lab room for uniform illumination.

Standard no distortion commercial USB video cameras with 2.8 mm Focal length, 1/2.7-in. CMOS, 120° field of view, 1024*768 @ 30 Hz and manual focus supported were used to record fly behaviors.

### Fly behavior recording software deployment

PotPlayer × 64 210318 (potplayer.daum.net) was used for camera recording. For better performance of fly detection and trajectory generation, GIMP 2.10.30 (GIMP—GNU Image Manipulation Program) and its plugin resynthesizer (https://github.com/bootchk/resynthesizer) was used for ghost fly removal from background image. UMATracker × 64 Release 14(UMATracker (ymnk13.github.io)) was used for fly trajectories generation. A self-developed software suit, *Drosophila* Video Tracking (DVT), was used for video clipping, background extraction and removal, and behavior analysis.

### Fly behavior criteria setup

Behavior criteria varied across literatures. Following we listed criteria used in this work and related considerations.

- Interaction criteria
  Sechneider et al. proposed an automated identification method of social interaction criteria in Drosophila by subtracting the simulated trails from the real ones. Three criteria, including encounter distance (about 1.25–3 body length), encounter angle (90–160°), and encounter time (0.4–1.1 s) was recommended for different species of flies [55]. Ctrax defined the interaction or encounter event as those trajectory intervals in which the distance between a pair of flies was less than 10 mm in which was ~ 3 body length [8]. Flytrack defined touch as a head-to tail touch event in a single frame. A touch interaction is obtained when a touch lasts for at least 15 consecutive frames (~ 0.5 s) [40].
  However, we noticed that Hoyer et al. reported that, a pair of five-day old CS males performed a complete lunges process in 118 ms in the aggression [56]. Hence, if we take interaction time larger than 0.4 s as our criteria, lots of interaction could be treated as false negatives. As reported by Simon et al., erroneous orientation was about 1 s^−1^ to 4 s^−1^ for individual flies [43], which means about 1800 ~ 7200 errors (1–4 s^−1^ * 30 min * 60 s) in a 30 min video. Thus, undirected social network was constructed to avoid orientation error in DVT paradigm. Therefore, we set the criteria as that the flies had an interaction from each other if the distance between them was less than 1.5 * body length in DVT.
- Move criteria
  Colomb et al. reported flies with velocity less than 1 mm/s was classified as at rest [10]. White et al. reported the threshold as 0.25 mm/s [57]. In DVT, the threshold was set to 0.5 mm/s. The fly was classified as at rest or inactive if its speed was lower than the threshold.
- Edge boundary
  We investigated the spatial preference of fly in DVT chamber. A peak emerged at 0.6 mm–3 mm from the arena edge in the fly spatial distribution. Hence, in DVT paradigm we set 3 mm from the arena edge as the boundary between edge and centre.
- Angular and meander time windows
  The time window of angular velocity and meander related features was set to 0.2 s which is consistent with Martin’s work [26]. 0.2 s is equivalent to the average step period at average speed 7.2 mm/s identified by Mendes’s work [58].
- Track straightness
  The time window of track straightness related features was set to 1 s which is consistent with Aggarwal’s work [44].
- Network size
  The Network size is set to 50% in DVT. That is the moving-social-network-window, representing 50% of the total number of interactions possible for flies. For example, the moving-social-network-window is 8 for a 6-fly social community in the chamber. DVT calculates the network features of the social network composed by the first 8 interactions and then the second network by the 9–16th interactions. For 8-fly community, the moving-social-network-window is 14. This calculation procedures were inherited from Schneider’s work [24].

### Fly preparation and behavior recording settings

Unless specifically stated, 6 mated male or female *w*^*1118*^ flies (5–7 days old) were collected under carbon dioxide anesthesia for each vial and kept on standard food medium one days prior to the experiment. The behavior lab room was maintained at 25 °C, 50–60% humidity and fly behavior videos were recorded between ZT01 and ZT04. All DVT experiment for each genotype or condition got 8–12 internal replicates and 3 independent repeats which generated 8–12*3 30 min videos except that the circadian behavior experiment of male flies from ZT00 to ZT11 quaque hora got 1 independent repeat. Detailed operations could be referred to the DVT manual available at Additional file 6: S6.

### Sleep deprivation

*W*^*1118*^ flies at 3 days old was collected and were fully sleep-deprived for 6 days using mechanical stimulation before behavior recording. The mechanical stimulation was powered by lab-made Arduino control oscillators and featured shaking of fly vials every 3 min at a randomly chosen 10-s time-window. The randomness can effectively prevent the fly from adopting vibration. This sleep deprivation strategy was inherited from REF [59].

### Social network simulation

Monte-Carlo simulation was designed to study the two newly proposed metrics, acquaintance and average number of crowded Drosophila. For acquaintance, totally 1e3 random weighted social networks were generated. For average number of crowded Drosophila, totally 1e3 trails were generated and each trails contains a random number between 600 to 1200 of social interactions to mimic a fly behavior video.

### Statistics and reproducibility

A modified Cohen’s d was used to measure standardized effect size in which the mean difference was divided by maximum value of standard deviations of the control group and treatment group. In general, an effect size of 0.2 or smaller is considered to be a small deviation, an effect size of around 0.5 is considered to be a medium deviation, and an effect size of 0.8 or larger is considered to be a large deviation.

ANOVA or mixed ANOVA were used to identify statistical significantly changed behavior metrics given experiment replicate and fly gender. False discovery rate (Benjamini/Hochberg FDR) by python *statsmodels* package 0.14.0 was performed for all radar plotted metrics.

For Fig. 6a and Additional file 8: Fig. S6, multi-level correlation was extracted to describe the relationship between metrics by correlation package in R 3.6.3 [48] with FDR adjustment.

### Supplementary Information

**Additional file 1**: S1 DVT behavior metrics definitions.

**Additional file 2**: S2 Fly behavior metrics categories in DVT.

**Additional file 3**: S3 Proof on equality of average-by-video value of behavior features before and after reID error correction.

**Additional file 4**: S4 Ground truth on reID error rate of fly videos.

**Additional file 5**: S5 Reported high-throughput paradigm practices.

**Additional file 6**: S6 Drosophila video track: DVT manual (v1.0).

**Additional file 7**: S7 Involved DVT experiments results collection.

**Additional file 8:** Supplemental Figures ** Figure S1** DVT analysis pipeline. A, Traditional analysis pipeline with reID error corrected. B, DVT analysis pipeline by average-by-video value of behavior metrics. C, Average-by-video fly behavior metrics has a strong correlation before and after reID error correction. D, Analysis pipeline for heterogeneous Dro. chamber. Statistical analysis: Pearson correlation were calculated between the average-by-video fly behavior metrics before and after reID error correction of n = 8 videos (4 videos from *w*^*1118*^ flies, and 4 videos from OR flies). **Figure S2** Behavior deviation for flies in 2 mm-height chamber. A, Effect size for locomotion behavior deviation of drosophila in 2 mm-height chamber from that in 3 mm-height chamber. A.1 ~ A.6, Scatter plots for track straightness at arena edge of male or female flies in 2 mm-height or 3 mm-height chamber from different experimental replicates. B, Effect size for social behavior deviation of drosophila in 2 mm-height chamber from that in 3 mm-height chamber. Interpretation of the radar plot: The effect size was calculated to illustrate the behavior feature deviation of flies in treatment group chambers from that in control chambers. In the calculation, the mean difference was divided by maximum value of standard deviations of the control group and treatment group to denote the effect size. In this case, the control group was fly behavior features in 3 mm-height chamber and the treatment group was that in 2 mm-height profile. In general, both the mean difference between two groups and the intragroup variance affected the magnitude of effect size. if the magnitude of effect size is smaller than 0.2, it is considered to be a small deviation, an effect size of around 0.5 is considered to be a medium deviation, and an effect size of 0.8 or larger is considered to be a large deviation. In the visualization, fly behavior in 3 mm-height chamber was set as the bench and represented by the bold black circle in the radar plot. Each dot in the plot represented the deviation of features from one experiment replicate. For example, we got six dots at the *Track straightness at arena edge*, from 3 male-fly experiment replicates and 3 female-fly experiment replicates (A.1 ~ A.6). Dots inner the bench circle indicated a decrease of features from control group. While dots outside the bench circle indicated an increase of features from control group. We also color-annotated the label of statistically significant changed features, red for increasing and green for decreasing with q-value asterisks. Statistical analysis: A modified Cohen’s d was used to calculate the effect size of behavioral deviation for flies in 2 mm-height chamber to that in 3 mm-height chamber from 3 experimental replicates. ANOVA were used to identify statistical significantly changed behavior metrics given experimental replicate and fly gender. False discovery rate (Benjamini/Hochberg FDR) was performed for p-value adjustment. Welch Two Sample t-test was used for comparisons between two groups in A.1 ~ A.6. *P < 0.05, **P < 0.01, ***P < 0.001. **Figure S3** Behavior deviation for flies with different density profiles. A, Effect size for locomotion behavior deviation of drosophila in 4-flies chamber from that in 6-flies chamber. B, Effect size for social behavior deviation of drosophila in 4-flies chamber from that in 6-flies chamber. C, Effect size for locomotion behavior deviation of drosophila in 8-flies chamber from that in 6-flies chamber. D, Effect size for social behavior deviation of drosophila in 8-flies chamber from that in 6-flies chamber. Statistical analysis: A modified Cohen’s d was used to calculate the effect size of behavioral deviation for flies with different density profiles from 3 experimental replicates. ANOVA were used to identify statistical significantly changed behavior metrics given experimental replicate and fly gender. False discovery rate (Benjamini/Hochberg FDR) was performed for p-value adjustment. Labels of statistically significant changed features were color-annotated, red for increasing and green for decreasing with q-value asterisks. *P < 0.05, **P < 0.01, ***P < 0.001. **Figure S4** Behavior deviation for flies in red dim darkness. A, Effect size for locomotion behavior deviation of drosophila in red dim darkness from that under normal Illumination. B, Effect size for social behavior deviation of drosophila in red dim darkness from that under normal Illumination. Statistical analysis: A modified Cohen’s d was used to calculate the effect size of behavioral deviation for flies in red dim darkness with that in normal Illumination from 3 experimental replicates. ANOVA were used to identify statistical significantly changed behavior metrics given experimental replicate and fly gender. False discovery rate (Benjamini/Hochberg FDR) was performed for p-value adjustment. Labels of statistically significant changed features were color-annotated, red for increasing and green for decreasing with q-value asterisks. *P < 0.05, **P < 0.01, ***P < 0.001. **Figure S5** Behavior deviation for flies with different experimental times. A, Effect size for locomotion behavior deviation of flies recorded at ZT06 from that at ZT01. B, Effect size for social behavior deviation of flies recorded at ZT06 from that at ZT01. C, Effect size for locomotion behavior deviation of flies recorded at ZT11 from that at ZT06. D, Effect size for social behavior deviation of flies recorded at ZT11 from that at ZT06. Statistical analysis: A modified Cohen’s d was used to calculate the effect size of behavioral deviation for flies with different experimental times from 3 experimental replicates. ANOVA were used to identify statistical significantly changed behavior metrics given experimental replicate and fly gender. False discovery rate (Benjamini/Hochberg FDR) was performed for p-value adjustment. Labels of statistically significant changed features were color-annotated, red for increasing and green for decreasing with q-value asterisks. *P < 0.05, **P < 0.01, ***P < 0.001. **Figure S6** Drosophila behavior feature correlation diagram. Statistical analysis: Multi-level correlation of paired behavior metrics was calculated to build the network. Red eclipses indicate positive correlation and green ones are for negative correlations. Only correlation with FDR q-value < 0.05 was colored. **Figure S7** Behavior deviation for flies at the last 5 min in the recorded video from that at the first 5 min. A, Effect size for locomotion behavior deviation for *w*^*1118*^ flies at the last 5 min in the video from that at the first 5 min. B, Effect size for social behavior deviation for *w*^*1118*^ flies at the last 5 min in the video from that at the first 5 min. C, Effect size for locomotion behavior deviation for OR flies at the last 5 min in the video from that at the first 5 min. D, Effect size for social behavior deviation for OR flies at the last 5 min in the video from that at the first 5 min. E, Effect size for locomotion behavior deviation for CS flies at the last 5 min in the video from that at the first 5 min. F, Effect size for social behavior deviation for CS flies at the last 5 min in the video from that at the first 5 min. Statistical analysis: A modified Cohen’s d was used to calculate the effect size of behavioral deviation for flies at the last 5 min in the recorded video compared with that at the first 5 min from 3 experimental replicates. Mixed ANOVA were used to identify statistical significantly changed behavior metrics given experimental replicate, fly gender, and video identity. False discovery rate (Benjamini/Hochberg FDR) was performed for p-value adjustment. Labels of statistically significant changed features were color-annotated, red for increasing and green for decreasing with q-value asterisks. *P < 0.05, **P < 0.01, ***P < 0.001. **Figure S8** Behavior deviation for male flies from female flies. A, Effect size for locomotion behavior deviation for *w*^*1118*^ male flies compared with *w*^*1118*^ female flies. B, Effect size for social behavior deviation for *w*^*1118*^ male flies compared with *w*^*1118*^ female flies. C, Effect size for locomotion behavior deviation for OR male flies compared with OR female flies. D, Effect size for social behavior deviation for OR male flies compared with OR female flies. E, Effect size for locomotion behavior deviation for CS male flies compared with CS female flies. F, Effect size for social behavior deviation for CS male flies compared with CS female flies. Statistical analysis: A modified Cohen’s d was used to calculate the effect size of behavioral deviation for male flies compared female flies from 3 experimental replicates. ANOVA were used to identify statistical significantly changed behavior metrics given experimental replicate, fly gender. False discovery rate (Benjamini/Hochberg FDR) was performed for p-value adjustment. Labels of statistically significant changed features were color-annotated, red for increasing and green for decreasing with q-value asterisks. *P < 0.05, **P < 0.01, ***P < 0.001.

## Data Availability

We provided all boxplot, as well as the temporal chart of behavior metrics for all experiments involved in this work at Additional file 7: Data S7. Our software is freely available at https://github.com/Xingyinliu-Lab/DVT. A detailed tutorial video guide is available on https://www.youtube.com/watch?v=LsdizdKKhrQ.
