# Supplementary material for: DVT: a high-throughput analysis pipeline for locomotion and social behavior in adult Drosophila melanogaster
Source: Cell Biosci. 2023 Oct 5;13:187. doi: 10.1186/s13578-023-01125-0 (PMC10557313; doi:10.1186/s13578-023-01125-0)
Supplement: Supplementary file 1 — Additional file 1: S1 DVT behavior metrics definitions. [file 13578_2023_1125_MOESM1_ESM.docx]

**S1 Fly behavior metrics definition in DVT**

DVT outputs 74 fly behavior features. Following gives an expounded definition on each behavior feature.

1. Exploration efficiency by time^Ξ^.

$$Exploration efficiency by time=\frac{The time used to complete 80\% of the arena exploration}{Video length}$$

2. Area explored by given time^Ξ^.

$$Area explored by given time=\frac{The arena exploration in a given time (10 minutes)}{The arena area}$$

3. Exploration efficiency by travel length^Ξ^.

$$Exploration efficiency by travel length=\frac{0.8}{Fly move length during the exploration of 80\% of the arena}$$

4. Avg. velocity^1^ (mm/s).

$$Avg. velocity=\frac{Fly move length}{Fly move time}$$

^1^ Note 1: the speed was below the lower threshold (0.5 mm/s) the fly was classified as at rest or inactive. Fly movement, including, velocity, move length, turning angular etc. was not taken into account.

1. Avg. velocity at arena edge^1,2 Ξ^ (mm/s).

$$Avg. velocity at arena edge=\frac{Fly move length at arena edge}{Fly move time at arena edge}$$

^2^ Note 2: 3mm to center from the circumference was considered as the arena edge. The remained arena was arena centre.

1. Avg. velocity at centre^1,2 Ξ^ (mm/s).

$$Avg. velocity at arena centre=\frac{Fly move length at arena centre}{Fly move time at arena centre}$$

2. Max. velocity^1,3 Ξ^. Maximum of fly moving velocity (mm/s).

^3^ Note 3: Given reID error might bring out possible sudden burst or changes of behavior features, DVT support both the mathematical maximum calculation or the robust maximum calculation. In the robust maximum calculation, the 95^th^ percentile was used to represent the maximum value by default. The threshold can be modified in DVT pipeline. The 100^th^ percentile of features is the mathematical maximum of features.

1. Max. velocity at arena edge^1,3 Ξ^. Maximum of fly moving velocity at arena edge (mm/s).
2. Max. velocity at centre^1 Ξ^. Maximum of fly moving velocity at arena centre (mm/s).
3. Total move length^1^. Fly total move length(mm).
4. Move length at arena edge ^Ξ^. Fly move length when moving at arena edge (mm).
5. Move length at arena centre ^Ξ^. Fly move length when moving at arena centre (mm).
6. Total move time^1^

$$Total move time=\frac{Fly move time}{Video length}$$

7. Move time prop. at arena edge ^Ξ^.

$$Move time prop. at arena edge=\frac{Fly move time at arena edge}{Total time spent at arena edge}$$

8. Move time prop. at arena centre ^Ξ^.

$$Move time prop. at arena centre=\frac{Fly move time at arena centre}{Total time spent at arena centre}$$

9. Avg. distance from the arena centre.

$$Avg. distance from the arena centre=\frac{Avg. distance from fly position to the centre}{Arena radius}$$

10. Time prop. spent at edge.

$$Time prop. spent at edge=\frac{Total time spent at arena edge}{Video length}$$

11. Movelength ratio at edge.

$$Movelength ratio at edge=\frac{Fly move length at arena edge}{Total move length}$$

12. Tracks number^4^. Counts of tracks.

^4^ Note 4: A track is defined as the path treaded by a fly when it moves continuously. This concept is inherited from Aggarwal’s work [1].

1. Avg. track duration. Average time duration of each track (s).
2. Avg. track length. Average path length of each track (mm).
3. Long stop episodes number^5^. Counts of long stop episodes.

^5^ Note 5: Inactivity with duration longer than 2s is defined as long stop episodes. If this parameter is modified to 5min, the long stop equals sleeping status[2].

1. Avg. inactivity duration. Average time duration of each inactivity episode (s).
2. Avg. long stop episodes duration. Average time duration of each long-stop episode (s).
3. Avg. track straightness^6^. Average straightness of track path.

^6^Note 6: The track straightness is the coefficient of determination, r^2^ value, of the linear regression model of the fly position in the time windows. The time window was set to 1s. This concept is inherited from Aggarwal’s work [1].

1. Track straightness at arena centre^6 Ξ^. Average straightness of track path when fly moves at the arena centre.
2. Track straightness at arena edge^6 Ξ^. Average straightness of track path when fly moves at the arena edge.
3. Avg. angular velocity^7^. Average angular velocity when fly is being moving (rad/s).

$$Avg. angular velocity=Average(\frac{Fly turning angle in the time window}{Time window size})$$

^7^, Note 7: the calculation on angular velocity, and meander, the time window was set to 0.2s to be consistent with Martin’s work [3].

1. Avg. angular velocity at arena centre ^Ξ^. Average angular velocity when fly is being moving at arena centre (rad/s).
2. Avg. angular velocity at arena edge ^Ξ^. Average angular velocity when fly is being moving at arena edge (rad/s).
3. Max. angular velocity^3 Ξ^. Maximum of fly angular velocity(rad/s).
4. Max. angular velocity at arena centre^3 Ξ^. Maximum of fly angular velocity at arena centre(rad/s).
5. Max. angular velocity at arena edge^3 Ξ^. Maximum of fly angular velocity at arena edge(rad/s).
6. Avg. meander (rad/mm)^7^.

$$Avg. meander=Average(\frac{Fly turning angle in the time window}{Fly move length in the time window})$$

7. Avg. meander at centre ^Ξ^ (rad/mm). Average meander when fly is being moving at arena centre.
8. Avg. meander at edge ^Ξ^ (rad/mm). Average meander when fly is being moving at arena edge.
9. Max. meander ^Ξ^ (rad/mm)^3^. Maximum of fly meander.
10. Max. meander at centre ^Ξ^ (rad/mm)^3^. Maximum of fly meander at arena centre.
11. Max. meander at arena edge ^Ξ^ (rad/mm)^3^. Maximum of fly meander at arena edge.
12. Acquaintance ^Ξ^.

$$\mathrm{Acquaintance}=\frac{\frac{The max interaction duration with other flies}{Total interaction duration with other flies}}{\frac{1}{number of flies in arena-1}}$$

13. Social space distance. Averaged the distance to the closest neighbor by frame (mm). This concepts comes from McNeil’s work [4]. An improvement in DVT is DVT calculate the space distance every frame in the video and take average of all frames. While McNeil’s work took one photo 30 minutes after flies were transferred to the chamber and calculate the distance according to that photo.
14. Space distance at arena edge ^Ξ^. Averaged the distance to the closest neighbor by frame when fly locates at the arena edge (mm).
15. Space distance at arena centre ^Ξ^. Averaged the distance to the closest neighbor by frame when fly locates at the arena centre (mm).
16. Space distance at activity episodes ^Ξ^. Averaged the distance to the closest neighbor by frame when fly is being moving (mm).
17. Space distance at inactivity episodes ^Ξ^. Averaged the distance to the closest neighbor by frame when fly is being inactive(mm).
18. SSI^8^. In fly SSI (social space index) calculation, the distances between the fly and other fly in every frame was binned by 5mm. The SSI is subtracting the percentage of flies in the 2^nd^ bin from the percentage of flies in the 1^st^ bin.

^8^, Note 8: This concept comes from Simon’s work [5].

1. SSI at arena edge ^Ξ^. Fly social space index when fly locates at the arena edge.
2. SSI at arena centre ^Ξ^. Fly social space index when fly locates at the arena centre.
3. SSI at activity episode s ^Ξ^. Fly social space index when fly is being moving.
4. SSI at inactivity episodes ^Ξ^. Fly social space index when fly is being inactive.
5. Total interaction duration^9^.

$$Total interaction duration=\frac{\sum Fly interaction time with other flies}{Video length}$$

^9^, Note 9: Interaction with different flies at the same time was treated as different interaction. The total interaction duration might be larger than 100% because there’s a chance fly interacts with two or more flies at the same time.

1. Interaction duration at edge ^Ξ^.

$$Interaction duration at edge=\frac{\sum Fly interaction time with other flies when fly locates at the arena edge}{Video length}$$

2. Interaction time prop. at edge ^Ξ^.

$$Interaction time prop. at edge=\frac{\sum Fly interaction time with other flies when fly locates at the arena edge}{Total time spent at arena edge}$$

3. Interaction duration at centre ^Ξ^.

$$Interaction duration at centre=\frac{\sum Fly interaction time with other flies when fly locates at the arena centre}{Video length}$$

4. Interaction time prop. at centre ^Ξ^.

$$Interaction time prop. at centre=\frac{\sum Fly interaction time with other flies when fly locates at the arena centre}{Total time spent at arena centre}$$

5. Interaction duration at activity episodes ^Ξ^.

$$Interaction duration at activity episodes=\frac{\sum Fly interaction time with other flies when fly is being moving}{Video length}$$

6. Interaction time prop. at activity episodes ^Ξ^.

$$Interaction time prop. at activity episodes=\frac{\sum Fly interaction time with other flies when fly is being moving}{Total move time}$$

7. Interaction duration at inactivity episodes ^Ξ^.

$$Interaction duration at inactivity episodes=\frac{\sum Fly interaction time with other flies when fly is being inactive}{Video length}$$

8. Interaction time prop. at inactivity episodes ^Ξ^.

$$Interaction time prop. at inactivity episodes=\frac{\sum Fly interaction time with other flies when fly is being inactive}{Video length-Total move time}$$

9. Total interaction duration at long-stop ^Ξ^.

$$Interaction duration at long-stop=\frac{\sum Fly interaction time with other flies when fly is at long stop episodes}{Video length}$$

10. Interaction time prop. at long-stop ^Ξ^.

$$Interaction time prop. at long-stop=\frac{\sum Fly interaction time with other flies when fly is at long stop episodes}{Duration time of all long stop episodes}$$

11. Interaction episode count. Number of Interaction episodes.
12. Interaction episode duration (s). Average duration of fly interaction episode.
13. Avg. number of crowded dro ^Ξ^. Average number of flies interact with the same fly simultaneously.
14. Degree assortativity coefficient^10^.

Assortativity measures the similarity of connections in the graph with respect to the node degree. The degree assortativity in a network is defined as the Pearson correlation coefficient of the degree of connected nodes, measured over the set of all edges.

^10^, Note 10: In DVT, the social network topology features including Degree assortativity coefficient, Clustering coefficient, Betweenness centrality, Network diameter, Network degree, Connected social network prop., Global efficiency, Closeness centrality, Eccentricity and Dominating is calculated and averaged for every iterative networks using a moving-social-network-window. The moving-social-network-window represents 50% of the total number of interactions possible for flies. For example, the moving-social-network-window is 8 for a 6-fly social community in the chamber. DVT calculates the network features for the social network composed by the first 8 interactions and then the second network by the 9^th^ to 16^th^ interactions. For 8-fly community, the moving-social-network-window is 14. This calculation procedures are inherited from Schneider’s work [6]. Definition about these features was quoted from [Degree assortativity (konect.cc)](http://www.konect.cc/statistics/assortativity/) and [Software for Complex Networks — NetworkX 2.8.4 documentation](https://networkx.org/documentation/stable/). Most calculation of the features are supported by NetworkX in python.

1. Clustering coefficient.

Clustering coefficient of a node in social network is the fraction of possible triangles through that node that exist.

1. Betweenness centrality.

Betweenness centrality of a node is the sum of the fraction of all-pairs shortest paths that pass through the node.

1. Network diameter ^Ξ^.

The diameter is the maximum eccentricity.

1. Network degree.

Degrees of nodes in the fly social network.

1. Unconnected social network prop ^Ξ^.

The unconnected social network proportion in all iterative networks separated by the moving-social-network-window.

1. Global efficiency.

The efficiency of a pair of nodes in a graph is the multiplicative inverse of the shortest path distance between the nodes. The global efficiency of a graph is the average efficiency of all pairs of nodes.

1. Closeness centrality^11 Ξ^.

Closeness centrality of a node is the reciprocal of the average shortest path distance to the node over all reachable nodes.

^11^, Note 11: Closeness centrality, Eccentricity and Dominating is available for fly individuals.

1. Eccentricity^11 Ξ^.

The eccentricity of a node is the maximum distance from the node to all other nodes in the social network.

1. Dominating^11 Ξ^.

A dominating set for social network with node set V is a subset D of V such that every node not in D is adjacent to at least one member of D.

^Ξ^, Note Ξ: newly proposed metrics.

Reference

1. Aggarwal, A., H. Reichert, and K. VijayRaghavan, *A locomotor assay reveals deficits in heterozygous Parkinson's disease model and proprioceptive mutants in adult Drosophila.* Proc Natl Acad Sci U S A, 2019. **116**(49): p. 24830-24839.

2. Shaw, P.J., et al., *Correlates of Sleep and Waking in Drosophila melanogaster.* Science, 2000. **287**(5459): p. 1834-1837.

3. Martin, J.R., *A portrait of locomotor behaviour in Drosophila determined by a video-tracking paradigm.* Behav Processes, 2004. **67**(2): p. 207-19.

4. McNeil, A.R., et al., *Conditions Affecting Social Space in Drosophila melanogaster.* J Vis Exp, 2015(105): p. e53242.

5. Simon, A.F., et al., *A simple assay to study social behavior in Drosophila: measurement of social space within a group.* Genes Brain Behav, 2012. **11**(2): p. 243-52.

6. Schneider, J., M.H. Dickinson, and J.D. Levine, *Social structures depend on innate determinants and chemosensory processing in Drosophila.* Proc Natl Acad Sci U S A, 2012. **109 Suppl 2**: p. 17174-9.
