# Supplementary material for: DVT: a high-throughput analysis pipeline for locomotion and social behavior in adult Drosophila melanogaster
Source: Cell Biosci. 2023 Oct 5;13:187. doi: 10.1186/s13578-023-01125-0 (PMC10557313; doi:10.1186/s13578-023-01125-0)
Supplement: Supplementary file 2 — Additional file 2: S2 Fly behavior metrics categories in DVT. [file 13578_2023_1125_MOESM2_ESM.docx]

**S2 Fly behavior metrics categories in DVT**

| # | Metric | Categories | Reference |
| --- | --- | --- | --- |
| 1 | Avg. velocity | Published metric | Ctrax ^1^, Martin et al.^2^, Schneider et al.^3^, IowaFLI Tracker^4^, ToxTrac^5^, Rooke et al.^6^, Alwash et al.^7^ |
| 2 | Total move length |  | Martin et al.^2^, Aggarwal et al.^8^, IowaFLI Tracker^4^, ToxTrac^5^, EasyFlyTracker^9^, Flytracker^10^, Besson et al.^11^ |
| 3 | Total move time |  | Martin et al.^2^, IowaFLI Tracker^4^, Buridan’s Paradigm^12^, Bath et al.^13^, Besson et al.^11^ |
| 4 | Avg. angular velocity |  | Ctrax ^1^, Martin et al.^2^, |
| 5 | Avg. meander |  | Martin et al.^2^, Buridan’s Paradigm^12^ |
| 6 | Avg. distance from the arena centre |  | Ctrax ^1^, ToxTrac^5^, Mohammad et al.^14^ |
| 7 | Time prop. spent at edge |  | Martin et al.^2^,Bath et al.^13^, Besson et al.^11^ |
| 8 | Move time prop. at arena edge |  | Bath et al.^13^ |
| 9 | Tracks number |  | Martin et al.^2^, Aggarwal et al.^8^, Buridan’s Paradigm^12^ |
| 10 | Avg. track duration |  | Martin et al.^2^, Aggarwal et al.^8^, Buridan’s Paradigm^12^ |
| 11 | Avg. track length |  | Aggarwal et al.^8^ |
| 12 | Avg. track straightness |  | Aggarwal et al.^8^ |
| 13 | Avg. inactivity duration. |  | Martin et al.^2^, Buridan’s Paradigm^12^ |
| 14 | Long stop episodes number |  | EasyFlyTracker^9^ |
| 15 | Avg. long stop episodes duration. |  | EasyFlyTracker^9^ |
| 16 | Social space distance |  | McNeil et al.^15^ |
| 17 | Social space index (SSI) |  | Simon et al.^16^ |
| 18 | Total interaction duration |  | Schneider et al.^3^, IowaFLI Tracker^4^ |
| 19 | Interaction episode count |  | Schneider et al.^3^, Rooke et al.^6^, Flytracker^10^, Alwash et al.^7^ |
| 20 | Interaction episode duration |  | Alwash et al.^7^ |
| 21 | Degree assortativity coefficient |  | Schneider et al.^3^, Flytracker^10^, Alwash et al.^7^ |
| 22 | Clustering coefficient |  | Schneider et al.^3^, Rooke et al.^6^, Flytracker^10^, Alwash et al.^7^ |
| 23 | Betweenness centrality |  | Schneider et al.^3^, Rooke et al.^6^, Flytracker^10^, Alwash et al.^7^ |
| 24 | Network degree |  | Schneider et al.^3^, Flytracker^10^ |
| 25 | Global efficiency |  | Schneider et al.^3^, Flytracker^10^, Alwash et al.^7^ |
| 26 | Avg. velocity at arena edge | Newly proposed metrics for characterization spatial pattern of fly locomotion behavior | |
| 27 | Avg. velocity at centre |  |  |
| 28 | Move length at arena edge |  |  |
| 29 | Move length at arena centre |  |  |
| 30 | Track straightness at arena centre |  |  |
| 31 | Track straightness at arena edge |  |  |
| 32 | Avg. angular velocity at arena centre |  |  |
| 33 | Avg. angular velocity at arena edge |  |  |
| 34 | Avg. meander at centre |  |  |
| 35 | Avg. meander at edge |  |  |
| 36 | Exploration efficiency by time |  |  |
| 37 | Area explored by given time |  |  |
| 38 | Exploration efficiency by travel length |  |  |
| 39 | Move time prop. at arena centre |  |  |
| 40 | Movelength ratio at edge |  |  |
| 41 | Space distance at arena edge | Newly proposed metrics for characterization spatial pattern of fly social behavior | |
| 42 | Space distance at arena centre |  |  |
| 43 | SSI at arena edge |  |  |
| 44 | SSI at arena centre |  |  |
| 45 | Interaction duration at edge |  |  |
| 46 | Interaction time prop. at edge |  |  |
| 47 | Interaction duration at centre |  |  |
| 48 | Interaction time prop. at centre |  |  |
| 49 | Max. velocity | Newly proposed metrics for characterization fly motion explosiveness | |
| 50 | Max. velocity at arena edge |  |  |
| 51 | Max. velocity at centre |  |  |
| 52 | Max. angular velocity |  |  |
| 53 | Max. angular velocity at arena centre |  |  |
| 54 | Max. angular velocity at arena edge |  |  |
| 55 | Max. meander |  |  |
| 56 | Max. meander at centre |  |  |
| 57 | Max. meander at arena edge |  |  |
| 58 | Space distance at activity episodes | Newly proposed metrics for characterization temporal pattern of fly social behavior | |
| 59 | Space distance at inactivity episodes |  |  |
| 60 | SSI at activity episodes |  |  |
| 61 | SSI at inactivity episodes |  |  |
| 62 | Interaction duration at activity episodes |  |  |
| 63 | Interaction time prop. at activity episodes |  |  |
| 64 | Interaction duration at inactivity episodes |  |  |
| 65 | Interaction time prop. at inactivity episodes |  |  |
| 66 | Total interaction duration at long-stop |  |  |
| 67 | Interaction time prop. at long-stop |  |  |
| 68 | Acquaintance | Newly proposed metrics for characterization fly social network. | |
| 69 | Avg. number of crowded dro. |  |  |
| 70 | Network diameter |  |  |
| 71 | Unconnected social network prop |  |  |
| 72 | Closeness centrality |  |  |
| 73 | Eccentricity |  |  |
| 74 | Dominating |  |  |

1. Branson, K., Robie, A.A., Bender, J., Perona, P. & Dickinson, M.H. High-throughput ethomics in large groups of Drosophila. *Nat Methods* **6**, 451-457 (2009).

2. Martin, J.R. A portrait of locomotor behaviour in Drosophila determined by a video-tracking paradigm. *Behav Processes* **67**, 207-219 (2004).

3. Schneider, J., Dickinson, M.H. & Levine, J.D. Social structures depend on innate determinants and chemosensory processing in Drosophila. *Proc Natl Acad Sci U S A* **109 Suppl 2**, 17174-17179 (2012).

4. Iyengar, A., Imoehl, J., Ueda, A., Nirschl, J. & Wu, C.F. Automated quantification of locomotion, social interaction, and mate preference in Drosophila mutants. *J Neurogenet* **26**, 306-316 (2012).

5. Rodriguez, A. et al. ToxTrac: A fast and robust software for tracking organisms. *Methods in Ecology and Evolution* **9**, 460-464 (2017).

6. Rooke, R., Rasool, A., Schneider, J. & Levine, J.D. Drosophila melanogaster behaviour changes in different social environments based on group size and density. *Commun Biol* **3**, 304 (2020).

7. Alwash, N., Allen, A.M., M, B.S. & Levine, J.D. The Drosophila melanogaster foraging gene affects social networks. *J Neurogenet* **35**, 249-261 (2021).

8. Aggarwal, A., Reichert, H. & VijayRaghavan, K. A locomotor assay reveals deficits in heterozygous Parkinson's disease model and proprioceptive mutants in adult Drosophila. *Proc Natl Acad Sci U S A* **116**, 24830-24839 (2019).

9. Qu, S. et al. EasyFlyTracker: A Simple Video Tracking Python Package for Analyzing Adult Drosophila Locomotor and Sleep Activity to Facilitate Revealing the Effect of Psychiatric Drugs. *Front Behav Neurosci* **15**, 809665 (2021).

10. Liu, G. et al. A simple computer vision pipeline reveals the effects of isolation on social interaction dynamics in Drosophila. *PLoS Comput Biol* **14**, e1006410 (2018).

11. Besson, M. & Martin, J.R. Centrophobism/thigmotaxis, a new role for the mushroom bodies in Drosophila. *J Neurobiol* **62**, 386-396 (2005).

12. Colomb, J., Reiter, L., Blaszkiewicz, J., Wessnitzer, J. & Brembs, B. Open source tracking and analysis of adult Drosophila locomotion in Buridan's paradigm with and without visual targets. *PLoS One* **7**, e42247 (2012).

13. Bath, E., Thomson, J. & Perry, J.C. Anxiety-like behaviour is regulated independently from sex, mating status and the sex peptide receptor in Drosophila melanogaster. *Animal Behaviour* **166**, 1-7 (2020).

14. Mohammad, F. et al. Ancient Anxiety Pathways Influence Drosophila Defense Behaviors. *Curr Biol* **26**, 981-986 (2016).

15. McNeil, A.R. et al. Conditions Affecting Social Space in Drosophila melanogaster. *J Vis Exp*, e53242 (2015).

16. Simon, A.F. et al. A simple assay to study social behavior in Drosophila: measurement of social space within a group. *Genes Brain Behav* **11**, 243-252 (2012).
