# Supplementary material for: DVT: a high-throughput analysis pipeline for locomotion and social behavior in adult Drosophila melanogaster
Source: Cell Biosci. 2023 Oct 5;13:187. doi: 10.1186/s13578-023-01125-0 (PMC10557313; doi:10.1186/s13578-023-01125-0)
Supplement: Supplementary file 3 — Additional file 3: S3 Proof on equality of average-by-video value of behavior features before and after reID error correction. [file 13578_2023_1125_MOESM3_ESM.docx]

**S3 Proof: the average-by-video value of behavior features before reID correction is equal to or expectedly equal to that after correction under assumption of chamber homogeneity**

To assess the reID error effect on the output features, without loss of generality, suppose the following situation we have $n$ flies in the chamber and the identity of the i^th^ fly and the j^th^ was exchanged at time $t$. That means an reID error occurred at time $t$ as following graph shows.


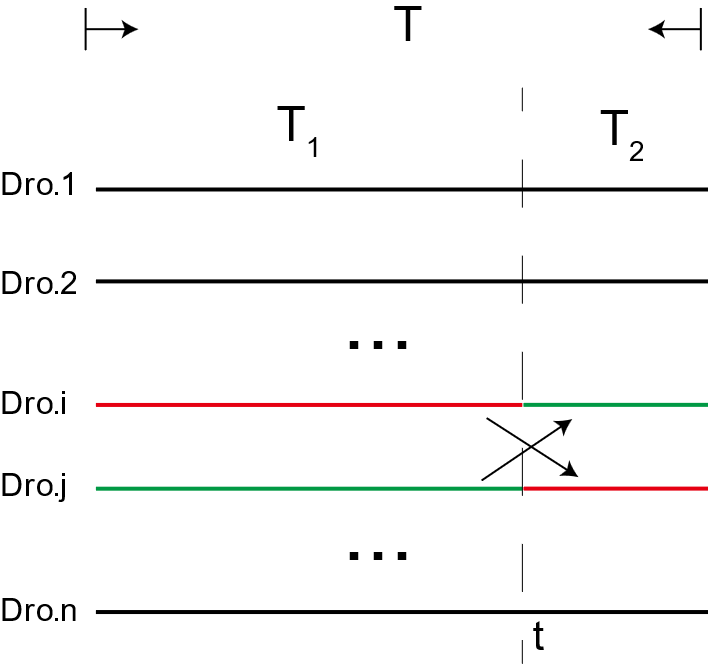


After the reID error, the velocity i^th^ and j^th^ fly can be calculated as:

$V_{i}^{'}=V_{i}^{'}(T)=\frac{V_{i}(T_{1})*T_{1}+V_{j}(T_{2})*T_{2}}{T_{1}+T_{2}}$ (1)

$V_{j}^{'}=V_{j}^{'}(T)=\frac{V_{j}(T_{1})*T_{1}+V_{i}(T_{2})*T_{2}}{T_{1}+T_{2}}$ (2)

Where $V_{i}(T)$ is the velocity of time period T for the i^th^ fly with correct ID. $V_{i}^{'}$is the velocity with reID error for the i^th^ fly. Hence the average velocity of the n flies in the chamber with reID error $\bar{V^{'}}$ can be calculated as:

$\bar{V^{'}}=\frac{V_{1}^{'}+V_{2}^{'}+\ldots+V_{i}^{'}+V_{j}^{'}+\ldots+V_{n}^{'}}{n}$ (3)

Because no reID error occurred for flies except i^th^ and j^th^. Thus

$V_{q}^{'}=V_{q}, q=1,2,\ldots n, q!=i and q!=j$ (4)

Where $V_{q}$ is the velocity with reID error corrected for the q^th^ fly.

Thus,

$$\bar{V^{'}}=\frac{V_{1}^{'}+V_{2}^{'}+\ldots+V_{i}^{'}+V_{j}^{'}+\ldots+V_{n}^{'}}{n}$$

$$=\frac{V_{1}+V_{2}+\ldots+V_{i}^{'}+V_{j}^{'}+\ldots+V_{n}}{n}$$

$$=\frac{V_{1}+V_{2}+\ldots+\frac{V_{i}\left( T_{1} \right)*T_{1}+V_{j}\left( T_{2} \right)*T_{2}}{T_{1}+T_{2}}+\frac{V_{j}\left( T_{1} \right)*T_{1}+V_{i}\left( T_{2} \right)*T_{2}}{T_{1}+T_{2}}+\ldots+V_{n}}{n}$$

$$=\frac{V_{1}+V_{2}+\ldots+\frac{V_{i}\left( T_{1} \right)*T_{1}+V_{i}\left( T_{2} \right)*T_{2}+V_{j}\left( T_{1} \right)*T_{1}+V_{j}\left( T_{2} \right)*T_{2}}{T_{1}+T_{2}}+\ldots+V_{n}}{n}$$

$$=\frac{V_{1}+V_{2}+\ldots+\frac{V_{i}\left( T_{1} \right)*T_{1}+V_{i}\left( T_{2} \right)*T_{2}}{T_{1}+T_{2}}+\frac{V_{j}\left( T_{1} \right)*T_{1}+V_{j}\left( T_{2} \right)*T_{2}}{T_{1}+T_{2}}+\ldots+V_{n}}{n}$$

$$=\frac{V_{1}+V_{2}+\ldots+V_{i}+V_{j}+\ldots+V_{n}}{n}$$

$$=\bar{V}$$

Therefore, the average velocity $\bar{V^{'}}$of the n flies in the chamber with reID error is equal to the average velocity $\bar{V}$ with error corrected. For several other metrics, for example, the *angular velocity*, the *social space distance*, formular 1 can be generalized into

$\omega_{i}^{'}=\frac{\omega_{i}(T_{1})*T_{1}+\omega_{j}(T_{2})*T_{2}}{T_{1}+T_{2}}$ (5)

Where $\omega_{i}(T)$ is the corresponding metric function of time period T, and $\omega_{i}^{'}$ is the metric with reID error for the i^th^ fly and similarly conclusions can be proved.

Some other metrics can be written into a different form. Take the *interaction episode count*, i.e., the total number of encounter event as an example, we have

$C_{i}^{'}=C_{i}^{'}(T)=C_{i}(T_{1})+C_{j}(T_{2})$ (6)

$C_{j}^{'}=C_{j}^{'}(T)=C_{j}(T_{1})+C_{i}(T_{2})$ (7)

Where $C_{i}(T)$ is the *interaction episode count* of time period T for the i^th^ fly with correct ID. $C_{i}^{'}$is the *interaction episode count* with reID error for the i^th^ fly. Hence the average *interaction episode count* of the n flies in the chamber with reID error $\bar{C^{'}}$ can be calculated as:

$$\bar{C^{'}}=\frac{C_{1}^{'}+C_{2}^{'}+\ldots+C_{i}^{'}+C_{j}^{'}+\ldots+C_{n}^{'}}{n}$$

$$=\frac{C_{1}+C_{2}+\ldots+C_{i}^{'}+C_{j}^{'}+\ldots+C_{n}}{n}$$

$$=\frac{C_{1}+C_{2}+\ldots+C_{i}(T_{1})+C_{j}(T_{2})+C_{j}(T_{1})+C_{i}(T_{2})+\ldots+C_{n}}{n}$$

$$=\frac{C_{1}+C_{2}+\ldots+C_{i}(T_{1})+C_{i}(T_{2})+C_{j}(T_{1})+C_{j}(T_{2})+\ldots+C_{n}}{n}$$

$$=\frac{C_{1}+C_{2}+\ldots+C_{i}+C_{j}+\ldots+C_{n}}{n}$$

$$=\bar{C}$$

This means the metrics, like *interaction episode count, tracks number,* *Total move time* etc. with a generalized formular 8, has an equal value of the average feature with or without reID error correction.

$\varphi_{i}^{'}=\varphi_{i}(T_{1})+\varphi_{j}(T_{2})$ (8)

The third type of metrics includes *Max. velocity*, *Max. angular velocity* etc. This type of metrics is function of probability density. Take the *max. velocity* for example, we have

$M_{i}^{'}=M_{i}^{'}(T)=\vartheta\left( f_{i}^{'}(T) \right)=\left\{ M_{i}^{'}:\int_{-\infty}^{M_{i}^{'}} f_{i}^{'}=0.95 \right\}$ (9)

$M_{j}^{'}=M_{j}^{'}(T)=\vartheta\left( f_{j}^{'}(T) \right)=\left\{ M_{j}^{'}:\int_{-\infty}^{M_{j}^{'}} f_{j}^{'}=0.95 \right\}$ (10)

Where the $M_{i}^{'}$ is the *Max. velocity* of time period T for the i^th^ fly with reID error. $f$is the empirical probability density of *velocity.* $\vartheta\left( f \right)$ denotes function of getting the 95^th^ percentile of features from probability density $f$. A notation is that we take the 95^th^ percentile of features as the maximum value in order to avoid potential misleading results brought out by reID or mis-detection error.

Assume that the chamber is in a homogeneous setting composed of individuals from the same strain or genotype and reared under same environment. Given the homogeneity, flies have a similar behavior pattern. Hence, the empirical probability density of *velocity* of the i^th^ fly is equal to that of the j^th^ fly in expectation. That is

$$f_{i}\left( T \right)\tilde{=}f_{j}\left( T \right)$$

Thus,

$$M_{i}^{'}=\vartheta\left( f_{i}^{'}\left( T \right) \right)$$

$$=\vartheta\left( \frac{f_{i}\left( T_{1} \right)*T_{1}+f_{j}\left( T_{2} \right)*T_{2}}{T_{1}+T_{2}} \right)$$

$$\tilde{=}\vartheta\left( \frac{f_{i}\left( T_{1} \right)*T_{1}+f_{i}\left( T_{2} \right)*T_{2}}{T_{1}+T_{2}} \right)$$

$\tilde{=}\vartheta\left( f_{i}\left( T \right) \right)$=$M_{i}$

Where $M_{i}$ is the *Max. velocity* of time period T for the i^th^ fly with reID error correction.

$$\bar{M^{'}}=\frac{M_{1}^{'}+M_{2}^{'}+\ldots+M_{i}^{'}+M_{j}^{'}+\ldots+M_{n}^{'}}{n}$$

$$=\frac{M_{1}+M_{2}+\ldots+M_{i}^{'}+M_{j}^{'}+\ldots+M_{n}}{n}$$

$$\tilde{=}\frac{M_{1}+M_{2}+\ldots+M_{i}+M_{j}+\ldots+M_{n}}{n}$$

=$\bar{M}$

Therefore, for metrics with a generalized form $\vartheta\left( f\left( T \right) \right)$, has an equal value in expectation of the average feature with or without reID error correction. In the above proof, instantaneous error state of metrics in the frames with reID error was ignored. For a 30-minutes-long 30 FPS video, totally 54000 frames, the affection from the instantaneous state of reID error frames was minor. The validation results in the main manuscript evidenced correction of our proof.

DVT established 74 metrics in total. All metrics can be put into the above three categories. Table below shows the category of all metrics confronted with the proof.

| Category | Metric |
| --- | --- |
| I | Exploration efficiency by time, Area explored by given time, Exploration efficiency by travel length, Avg. velocity, Avg. velocity at arena edge, Avg. velocity at centre, Avg. distance from the arena center, Avg. track duration, Avg. track length, Avg. inactivity duration, Avg. long stop episodes duration, Avg. track straightness, Track straightness at arena centre, Track straightness at arena edge, Avg. angular velocity, Avg. angular velocity at arena centre, Avg. angular velocity at arena edge, Avg. meander, Avg. meander at centre, Avg. meander at arena edge, Acquaintance, Social space distance, Space distance at arena edge, Space distance at arena centre, Space distance at activity episodes, Space distance at inactivity episodes, SSI, SSI at arena edge, SSI at arena centre, SSI at activity episodes, SSI at inactivity episodes, Interaction episode duration, Avg. number of crowded dro. , Degree assortativity coefficient, Clustering coefficient, Betweenness centrality , Network diameter, Network degree, Unconnected social network prop., Global efficiency |
| II | Total move length, Move length at arena edge, Move length at arena centre, Total move time, Move time prop. at arena edge, Move time prop. at arena centre, Time prop. spent at edge, Movelength ratio at edge, Tracks number, Long stop episodes number, Total interaction duration, Interaction duration at edge, Interaction time prop. at edge, Interaction duration at centre, Interaction time prop. at centre, Interaction duration at activity episodes, Interaction time prop. at activity episodes, Interaction duration at inactivity episodes, Interaction time prop. at inactivity episodes, Total interaction duration at long-stop, Interaction time prop. at long-stop, Interaction episode count |
| III | Max. velocity, Max. velocity at arena edge, Max. velocity at centre, Max. angular velocity, Max. angular velocity at arena centre, Max. angular velocity at arena edge, Max. meander, Max. meander at centre, Max. meander at arena edge |
