# Supplementary material for: DVT: a high-throughput analysis pipeline for locomotion and social behavior in adult Drosophila melanogaster
Source: Cell Biosci. 2023 Oct 5;13:187. doi: 10.1186/s13578-023-01125-0 (PMC10557313; doi:10.1186/s13578-023-01125-0)
Supplement: Supplementary file 4 — Additional file 4: S4 Ground truth on reID error rate of fly videos. [file 13578_2023_1125_MOESM4_ESM.docx]

**S4 Ground truth on reID error rate of fly videos^1^**

| id | Genotype | Sex | Velocity  (mm/s) | Move time  (%) | Interaction events counts | reID error count | reID error rate (%)^3^ |
| --- | --- | --- | --- | --- | --- | --- | --- |
| 1 | Canton-S | M^2^ | 1.852 | 13.3 | 183 | 0 | 0 |
| 2 | Canton-S | M | 4.094 | 16.5 | 267 | 0 | 0 |
| 3 | Canton-S | F^2^ | 2.02 | 7.9 | 114 | 0 | 0 |
| 4 | Canton-S | F | 2.56 | 9.2 | 90 | 0 | 0 |
| 5 | Oregon-R | M | 7.181 | 30.2 | 659 | 4 | 0.607 |
| 6 | Oregon-R | F | 4.714 | 68.5 | 1136 | 3 | 0.264 |
| 7 | Oregon-R | F | 5.972 | 62.6 | 1201 | 6 | 0.5 |
| 8 | Oregon-R | M | 6.545 | 46.6 | 1118 | 7 | 0.626 |
| 9 | *w^1118^* | F | 4.583 | 70.6 | 1250 | 35 | 2.8 |
| 10 | *w^1118^* | M | 5.311 | 62.3 | 1302 | 18 | 1.382 |
| 11 | *w^1118^* | F | 4.629 | 67.1 | 1181 | 33 | 2.794 |
| 12 | *w^1118^* | M | 7.888 | 86.2 | 2273 | 34 | 1.496 |

^1^: All videos had a length of 30 minutes.

^2^: F for female, M for male flies.

^3^: The reID error rate is calculated as ratio from reID error count to the interaction/encounter events counts.
