# Supplementary material for: DVT: a high-throughput analysis pipeline for locomotion and social behavior in adult Drosophila melanogaster
Source: Cell Biosci. 2023 Oct 5;13:187. doi: 10.1186/s13578-023-01125-0 (PMC10557313; doi:10.1186/s13578-023-01125-0)
Supplement: Supplementary file 5 — Additional file 5: S5 Reported high-throughput paradigm practices. [file 13578_2023_1125_MOESM5_ESM.docx]

**S5 Reported high-throughput paradigm practices**

| Reference | Paradigm | Chamber design | | | Number of flies in the chamber | Prior acclimate | Video length piped into analysis | Experiment time (Zeitgeber time) |
| --- | --- | --- | --- | --- | --- | --- | --- | --- |
|  |  | Size(mm) | Height(mm) | shape |  |  |  |  |
| [1] | Ctrax, general-purpose | 245 | 245, open chamber with wing clipped | circular arena | 1 or 20 | Not reported | 30mins | Not reported |
| [2] | IowaFLI Tracker, general-purpose | 35 | 2 | circular arena | 6+ | 0 | 15mins | Not reported |
| [3] | Buridan’s Paradigm with CeTrAn, general-purpose | 117 | 313, open chamber with wing clipped | circular arena | 1 | Not reported | 15mins | Not reported |
| [4] | ToxTrac, general-purpose | Not reported | Not reported | rectangular arena | Less than 10–20 | Not reported | Not reported | Not reported |
| [5] | EasyFlyTracker, general-purpose | 16 | 3 | circular arena | 1 | Not reported | 3hours | Not reported |
| [6] | Social interaction network topology | 30~120 | Not reported | circular arena | 6~24 | 10mins | 30mins | ZT2-ZT4 |
| [7] | Flytracker | 120 | 3.5 | circular arena | 1, 16 and 50 | 1mins | 60mins | ZT0 |
| [8] | Social interaction network topology | 60 | 2 | circular arena | 12 | Not reported | 30mins | ZT9.5 |
| [9] | Social cluster | 90 | 12 | circular arena | 50 | 0 | 2hours | ZT1-ZT7 |
| [10] | Centrophobism/Thigmotaxis | 10*10 | 1.6 | Squared arena | 1 | Not reported | 10mins | Not reported |
| [11] | Centrophobism/Thigmotaxis | 54 | 5 | circular arena | 1 | 5mins | 10mins | ZT1-ZT7 |
| [12] | Centrophobism/Thigmotaxis | 40*40 | 3.5 | Squared arena | 1 | Not reported | 4hours | Not reported |
| [13] | Persistent counter-clockwise or clockwise walking pattern in a circular arena | 12.7 | 3 | circular arena | 1 | 5mins | 5mins | Not reported |
| [14] | Zigzag walking pattern | 40*40 | 3.5 | Squared arena | 1 | Not reported | 7hours | Not reported |

1. Branson, K., et al., *High-throughput ethomics in large groups of Drosophila.* Nat Methods, 2009. **6**(6): p. 451-7.

2. Iyengar, A., et al., *Automated quantification of locomotion, social interaction, and mate preference in Drosophila mutants.* J Neurogenet, 2012. **26**(3-4): p. 306-16.

3. Colomb, J., et al., *Open source tracking and analysis of adult Drosophila locomotion in Buridan's paradigm with and without visual targets.* PLoS One, 2012. **7**(8): p. e42247.

4. Rodriguez, A., et al., *ToxTrac: A fast and robust software for tracking organisms.* Methods in Ecology and Evolution, 2017. **9**(3): p. 460-464.

5. Qu, S., et al., *EasyFlyTracker: A Simple Video Tracking Python Package for Analyzing Adult Drosophila Locomotor and Sleep Activity to Facilitate Revealing the Effect of Psychiatric Drugs.* Front Behav Neurosci, 2021. **15**: p. 809665.

6. Rooke, R., et al., *Drosophila melanogaster behaviour changes in different social environments based on group size and density.* Commun Biol, 2020. **3**(1): p. 304.

7. Liu, G., et al., *A simple computer vision pipeline reveals the effects of isolation on social interaction dynamics in Drosophila.* PLoS Comput Biol, 2018. **14**(8): p. e1006410.

8. Alwash, N., et al., *The Drosophila melanogaster foraging gene affects social networks.* J Neurogenet, 2021. **35**(3): p. 249-261.

9. Jiang, L., et al., *Emergence of social cluster by collective pairwise encounters in Drosophila.* Elife, 2020. **9**.

10. Mohammad, F., et al., *Ancient Anxiety Pathways Influence Drosophila Defense Behaviors.* Curr Biol, 2016. **26**(7): p. 981-6.

11. Bath, E., J. Thomson, and J.C. Perry, *Anxiety-like behaviour is regulated independently from sex, mating status and the sex peptide receptor in Drosophila melanogaster.* Animal Behaviour, 2020. **166**: p. 1-7.

12. Besson, M. and J.R. Martin, *Centrophobism/thigmotaxis, a new role for the mushroom bodies in Drosophila.* J Neurobiol, 2005. **62**(3): p. 386-96.

13. Xiao, C., S. Qiu, and R.M. Robertson, *Persistent One-Way Walking in a Circular Arena in Drosophila melanogaster Canton-S Strain.* Behav Genet, 2018. **48**(1): p. 80-93.

14. Martin, J.R., *A portrait of locomotor behaviour in Drosophila determined by a video-tracking paradigm.* Behav Processes, 2004. **67**(2): p. 207-19.
