# Supplementary material for: DVT: a high-throughput analysis pipeline for locomotion and social behavior in adult Drosophila melanogaster
Source: Cell Biosci. 2023 Oct 5;13:187. doi: 10.1186/s13578-023-01125-0 (PMC10557313; doi:10.1186/s13578-023-01125-0)
Supplement: Supplementary file 6 — Additional file 6: S6 Drosophila video track: DVT manual (v1.0). [file 13578_2023_1125_MOESM6_ESM.pdf]

Drosophila video track: DVT manual

v1.0

Kai Mi, Yiqing Li @Xingyin Liu Lab
20220816

Contents

1. Overview 4
2. Hardware implementation 5
2.1. Camera specification 5
2.2. Chamber preparation 5
2.2.1. Chamber components 5
2.2.1. Make the spacer and plug 5
2.2.2. Assemble the chamber 6
2.3. Build the camera brackets 7
2.4. Build the drosophila transfer funnel 9
2.5. Lighting set up 9
3. Software deployment 10
3.1. DVT desktop edition software deployment 10
3.1.1. Feature supported 10
3.1.2. Software deployment 10
3.2. DVT server edition software deployment 10
3.2.1. Feature supported 10
3.2.2. DVT server edition deployment 10
3.2.3. DVT metahelper deployment 11
3.3. Other software used in DVT analysis 11
4. Recommended practices 12
4.1. Experiment design and conduction 12
4.1.1. DVT experiment design tips 12
4.1.2. Drosophila preparation 12
4.1.3. Drosophila behavior lab room preparation 12
4.1.4. Camera setup 12
4.1.5. Drosophila transference 13
4.1.6. Video recording 14
4.2. Data analysis by DVT desktop edition 15
4.2.1. Generate background image and clip video 15
4.2.2. Remove fly ghost in background images 16
4.2.3. Arena identification and background removal 19
4.2.4. Detect fly trace 19
4.2.5. Fly behavior analysis 21
4.3. Data analysis by DVT server edition 21
4.3.1. Transfer recorded video to the server 21
4.3.2. Clip the video and extract background images 22
4.3.3. Collect meta info 22

4.3.4. Remove background and scale the video 25
4.3.5. Fly behavior analysis and visualization....................25
4.4. Results interpretation....................26
4.4.1. Fly behavior features definition 26
4.4.2. Analysis data generated by DVT....................31
4.4.3. Analysis output file definition 37
4.4.4. Use fishbone diagram in result interpretation....................41
4.4.5. Track straightness related features and Network topology features interpretation 42
4.5. Behavior criteria practices and DVT analysis parameter configuration....................44
4.5.1. Considerations on behavior criteria setup....................44
4.5.2. DVT analysis parameter configuration....................45
Reference 49

Drosophila video track: DVT manual (v1.0)

1. Overview

Drosophila Video Tracking (DVT) is a comprehensive, and standardized high-throughput paradigm for drosophila locomotion and social behavior analysis. From the video acquisition to behavior analysis, the DVT paradigm covers the whole process of a fly tracking experiment. This manual made a full detailed instruction on the whole pipeline of video-tracking-based fly behavior quantification, including hardware preparation, experiment design and video acquisition, data analysis and result interpretation.

Quick start:

Video tutorial:

@figshare https://figshare.com/articles/media/DVT_protocol_v1_0_20221013/21325701

@Youtube https://youtu.be/LsdizdKKhrQ

Demo videos:

w1118 flies behavior video:

@Youtube https://youtu.be/GN9cKaYkeu8

w1118 flies behavior video with background removed:

@Youtube https://youtu.be/Qva7o2YA_AQ

w1118 flies behavior video with identify annotated:

@Youtube https://youtu.be/z7oV6d0CIHY

Github repositories: https://github.com/Xingyinliu-Lab/DVT

2. Hardware implementation

2.1. Camera specification

Standard no distortion commercial USB video camera with

- 2.8mm Focal length
- 1/2.7 inch CMOS
- 120-degree field of view
- 1024*768 @ 30Hz supported
- Manual focus

2.2. Chamber preparation

2.2.1. Chamber components

Figure 1 showed the chamber components and its size. The ceiling and floor are made of glasses ((50mm by 50mm, with a thickness of 1mm). The spacer and the plug are cut from 3mm thick transparent acrylic sheet (fig 1).

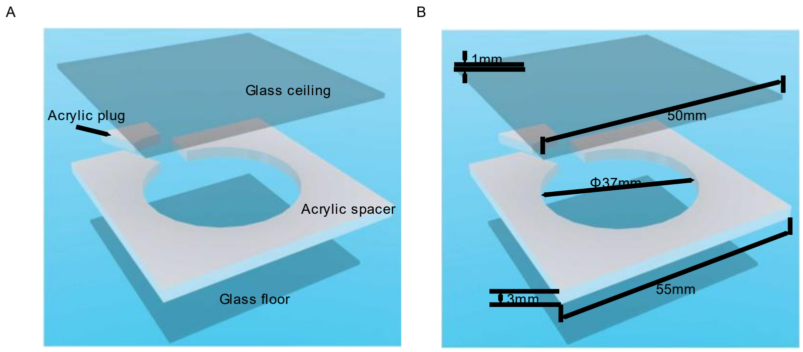

Fig 1, chamber components. A, the chamber consists of two square glass panes, the spacer, and the plug. B, the chamber components size.

2.2.1. Make the spacer and plug

Order or make the pre-acrylic-spacer with 3mm-thick transparent acrylic sheet. The pre-acrylic spacer is 55mm by 55mm squared. Usually, the hole in the middle is cut with a laser engraving machine. The engraved pre spacer is shown in fig 2A and in fig2B, a CAD sketch is provided.

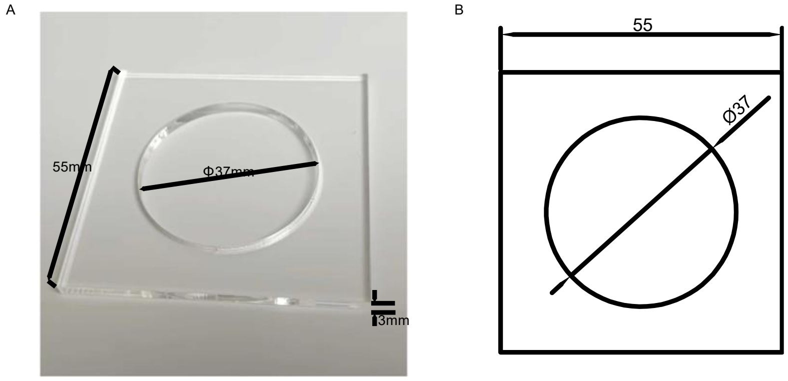

Fig 2, chamber pre-spacer. A, a processed pre-spacer. B, CAD sketch for pre spacer.

Mark the position of plug in the pre-spacer and curve out the cutting line by acrylic cutter. Break off the plug from the pre-spacer along the curved line (Fig 3). Do not using the laser engraving machine. The cutting loss from machine will lead to an imperfectly fit between the plug and the spacer.

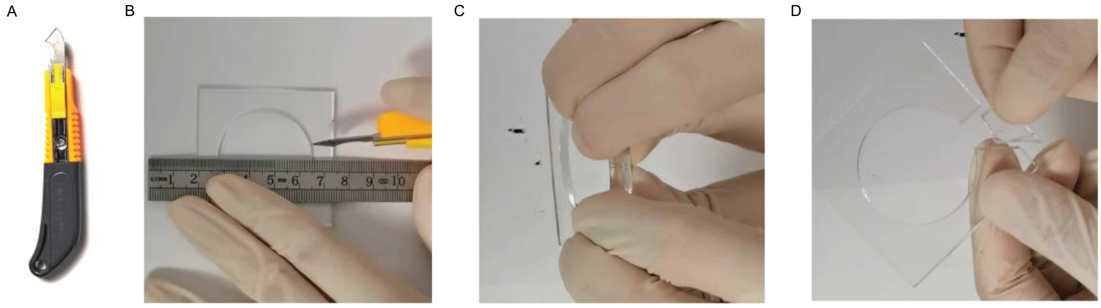

Fig 3, chamber plug and spacer handcrafting. A, an acrylic cutter. B, curve out the cutting line at the pre-spacer. C, break off the plug from the spacer by hand. D, the handcrafted plug and spacer.

Due to handcrafting, the plug varies from each other slightly. But the plug should be conical shaped for the convenience of drosophila transfer. Figure 4 gives demonstration on qualified acrylic plugs (fig 4, A, B) and unqualified ones (fig 4, C, D).

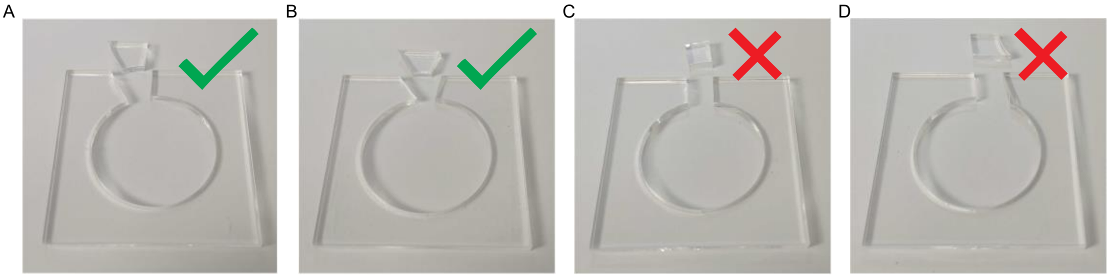

Fig 4, varied plug shapes. A-B, qualified acrylic plugs. C-D, unqualified acrylic plugs.

2.2.2. Assemble the chamber

Prepare all components needed to assemble the chamber (fig 5, A-C). Place the glass panes as ceiling and floor of the chamber (fig 5, D). Use bind chips to secure the glass ceiling and floor together with the spacer (fig 5, E). Put the plug in the spacer (fig 5, F). Use the third bind chip to secure the plug (fig 5, G). Please make sure using bind chips with the same color and clamping at similar positions cross chambers.

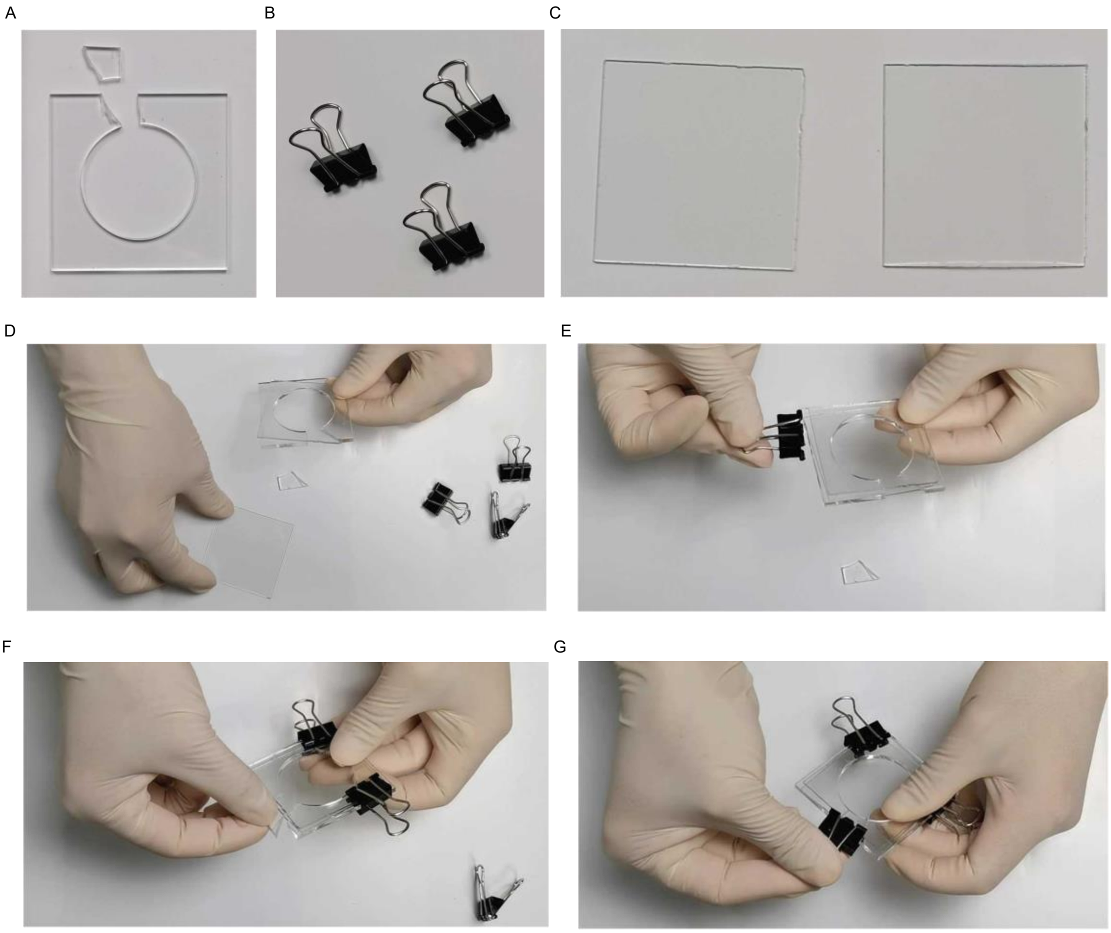

Fig 5, chamber assembling. A-C, chamber components, the spacer, plug, binder clips, and two glass panes. D-G, chamber assembling procedures.

2.3. Build the camera brackets

The camera bracket is made of 3mm-thick transparent acrylic sheet. Fig 6 gives the view and the CAD sketch of the bracket (fig 6). In the bracket, the floor is 120mm by 120mm squared acrylic sheet with four columns glued on it. The cover is a little bit complex with a center hold to pin the camera lens in and four positioning holes at corners (fig 6, D). The height of column, and the size of the center hole in the bracket cover might be adjusted according to the camera specifications to make sure that the camera can be focused on the chamber and shoot the whole chamber view.

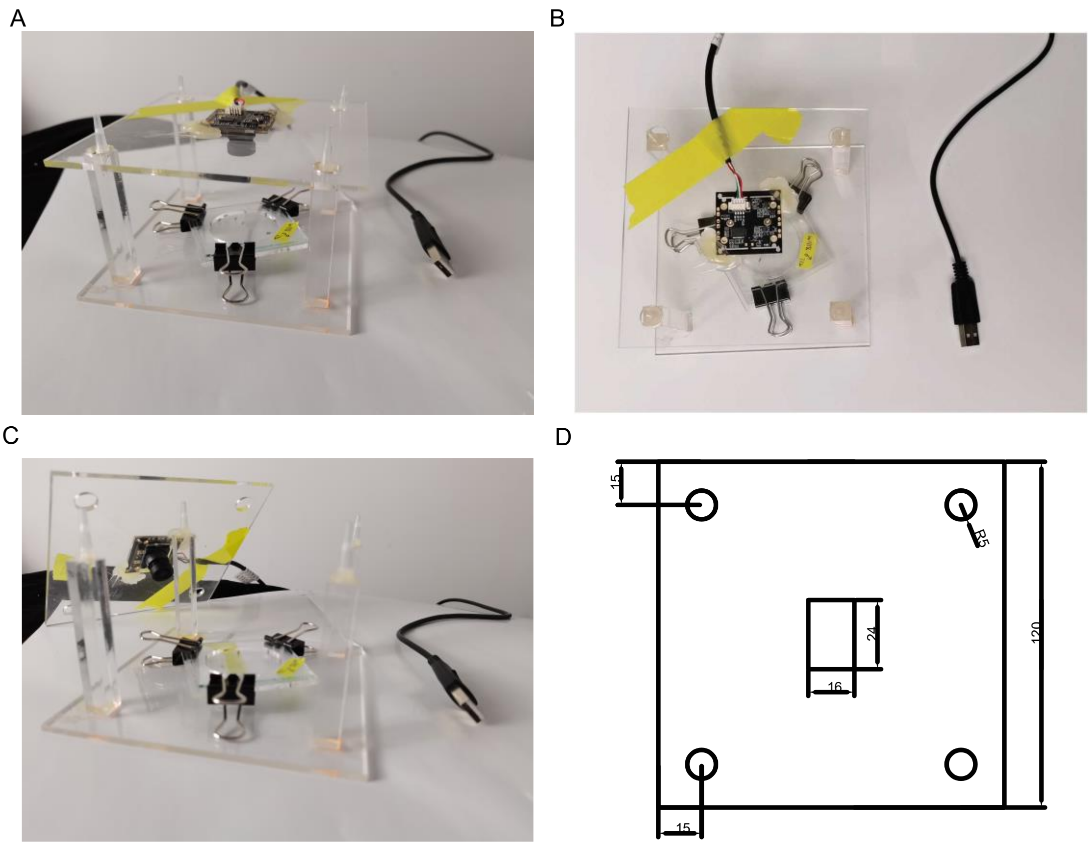

Fig 6, Camera brackets. A, front view of the camera bracket. B, top view of the camera bracket. C, the camera is pinned to the center hole of the bracket cover. D, CAD sketch for the bracket cover.

Usually, we set up multi cameras to recording multi chambers simultaneously. In this case, a bookshelf would be a good solution. As shown in fig 7, we have 12 cameras placed on the white foam board in the shelf in our lab room.

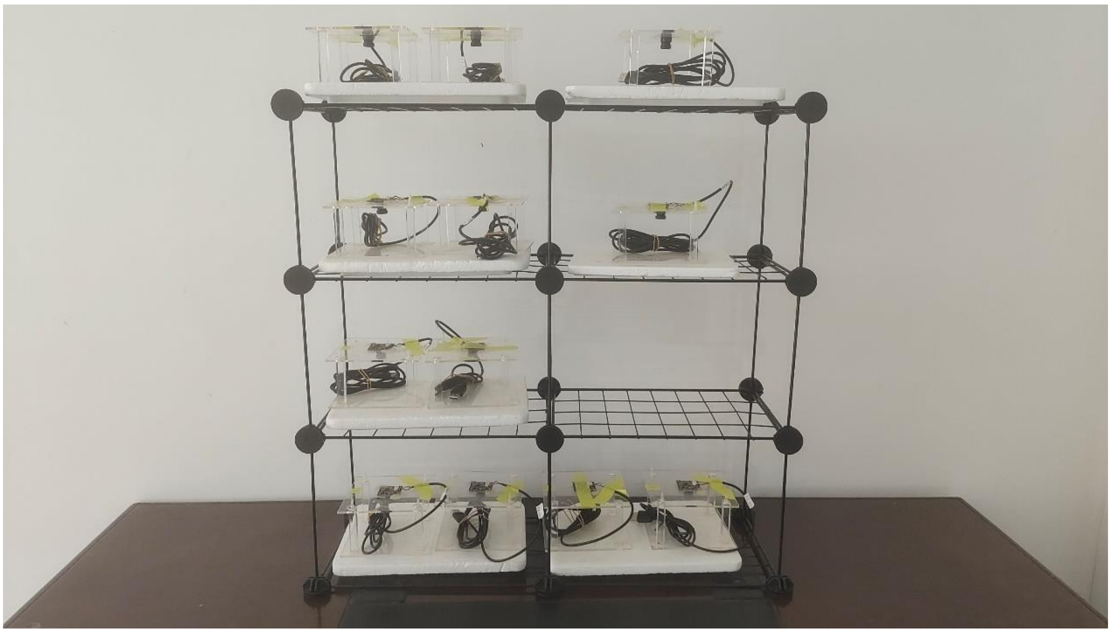

Fig 7, Camera shelf.

2.4. Build the drosophila transfer funnel

The drosophila is transferred into the chamber through the plug hole. To conduct the transference, the drosophila transfer funnel is built by 50mL centrifuge tube and 10ml pipette tip. Cut the tube at one third by a saw (fig 8, A) and remove the tube bottom (fig 8, B). Truncate the pipette tip by scissors (fig 8, D, E). Combine the tube and the pipette tip by adhesive tape and you'll get the transfer funnel (fig 8, F, G).

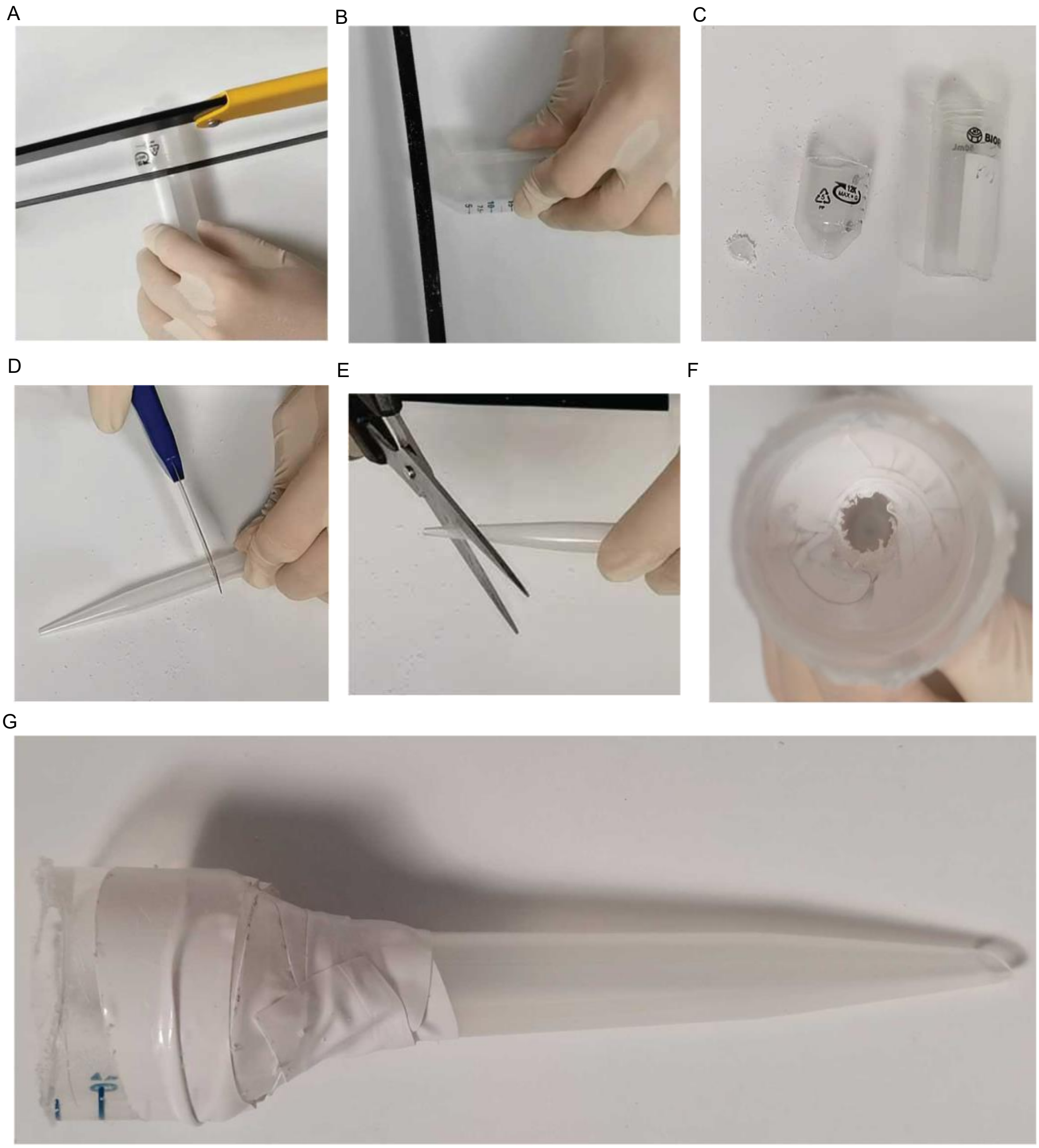

Fig 8, drosophila transfer funnel. A, cut the centrifuge tube at about one third. B, cut off the bottom of the tube. C, the upper part of the funnel. D, cut the pipette tip in the middle. E, truncate the pipette tip by scissors. F, top view of the funnel. G, front view of the funnel.

2.5. Lighting set up

Bright and uniform lighting is important in the video recording. We use two 50x70CM soft-box with 150W LED photography kits as the lighting source in the 3m*4m drosophila behavior lab room. The lighting might be changed according to the room size or wall color of the behavior room.

3. Software deployment

We provided both the server edition and the desktop edition software to help researcher conduct the fly behavior analysis. The server edition supports batch analysis and is suitable for coding and Linux experienced researchers. The desktop edition supports graphical user interface and runs on personal computer which is friendly to researchers with no coding or no Linux experience.

3.1. DVT desktop edition software deployment

3.1.1. Feature supported

- Video clip. Clip the video according to the specified start and end points. The clipped video will be piped into the following analysis.
- Video background extraction and removal. Extract and generate the video background from the clipped video. Remove background from the video and piped the video into next analysis step.
- Locomotion and social behavior analysis. Analysis on the whole chip of data. The analysis results show a general summary of drosophila behavior across the whole video.
- Visualization. Trace plot. Velocity, and social space distance time series plot.
- Video annotation. Mark the id and historical trace on drosophila in the video.
- Graphical user interface supported.

3.1.2. Software deployment

DVT desktop edition is running at Microsoft Windows 7/ 10/ 11 @1920*1024. Download DVT desktop edition from DVT/DVT desktop edition/release at main · Xingyinliu-Lab/DVT (github.com). Double-Click the file and DVT desktop edition is ready to use.

3.2. DVT server edition software deployment

3.2.1. Feature supported

- Video clip. Clip the video according to the specified start and end points. The clipped video will be piped into the following analysis.
- Video background extraction and removal. Extract and generate the video background from the clipped video. Remove background from the video and piped the video into next analysis step.
- Locomotion and social behavior analysis
  - Analysis on the whole chip of data. The analysis results show a general summary of drosophila behavior across the whole video.
  - Analysis on the sliced timeline. The analysis results show drosophila behavior changes across the moving time window.
- Visualization. Trace plot. Velocity, and social space distance time series plot. Grouped boxplot or violin plot, and timeseries plot of drosophila behavior.
- Video annotation. Mark the id and historical trace on drosophila in the video.
- Batch analysis support on the above features.

3.2.2. DVT server edition deployment

The software deployment has been tested on Ubuntu 20.04 LTS/ Ubuntu 16.04 LTS/ Centos-release-7-9.2009.1.el7.centos.x86_64. Miniconda is used to deploy and manage the software environment. Miniconda acquisition and installing introduction can be found at https://docs.conda.io/projects/conda/en/latest/user-guide/install/download.html.

```
conda create -n DVT python=3.7
```

Activate the environment.

```
1. conda activate DVT
```

Git clone the source from Github and move into the directory.

```
1. git clone https://github.com/Xingyinliu-Lab/DVT.git
   Drosophila_track_DVT
2. cd Drosophila_track_DVT/
```

Go to the DVT server edition/source folder and install dependent python packages.

```
1. pip install -r requirements.txt
```

Or install the packages manually.

```
1. pip install numpy==1.20.1
2. pip install pandas==1.2.3
3. pip install scipy==1.6.1
4. pip install scikit-learn==0.24.1
5. pip install opencv-python==4.5.1.48
6. pip install networkx==2.5.1
7. pip install matplotlib==3.3.4
8. pip install moviepy==1.0.3
9. pip install seaborn==0.11.2
```

3.2.3. DVT metahelper deployment

To help preparing the meta info used in the batch analysis of DVT server edition, we provided DVT metahelper. DVT metahelper is running at Microsoft Windows 7/ 10/ 11 @1920*1024. Download DVT metahelper from DVT/DVT_metahelper/release at main · Xingyinliu-Lab/DVT (github.com). Double-Click the file and DVT metahelper is ready to use.

3.3. Other software used in DVT analysis

- Adobe Photoshop 2021 Official Adobe Photoshop | Photo and design software or GIMP 2.10.30 GIMP - GNU Image Manipulation Program
- PotPlayer x64 210318 potplayer.daum.net
- UMATracker x64 Release 14 UMATracker (ymnk13.github.io)

Note: Adobe Photoshop or GIMP is used to remove the drosophila ghost in the generated background image. PotPlayer is used to record the video. UMATracker is used to detect drosophila traces. In this four software, GIMP, PotPlayer and UMATracker are free of charge. We have tested the four software. Other versions of software with similar functions are ok.

4. Recommended practices

4.1. Experiment design and conduction

4.1.1. DVT experiment design tips

The three basic experiment design principles, Randomization, Replication and Local Control should be obeyed in DVT experiment design. If we go into details, we have serval tips in the DVT experiment design.

- The number of flies in the chamber should be strictly control. Make sure all your chambers have same number of flies recorded.
- Perform the experiment between Zeitgeber time 01 and ZT04.
- Maintain bright and uniform lighting.
- Setup multi cameras and fly behavior in different groups should be recorded at the same time. Do not record treatment flies after the control ones. Fly behavior varies across the Zeitgeber time.

4.1.2. Drosophila preparation

One to two days prior to the experiment, collect 6 flies under cold anesthesia or carbon dioxide anesthesia. Maintain flies in vials containing standard Drosophila fly food with 6 flies per vial (fig 9). Label the vial properly with the genotype, sex, treatment, date information. We highly recommend 1-2 spare vials for each group in the drosophila preparation. For example, if 12 vials of flies in each group are going to be recording, it is better to prepare 14 vials for each group in case of failure during drosophila transference.

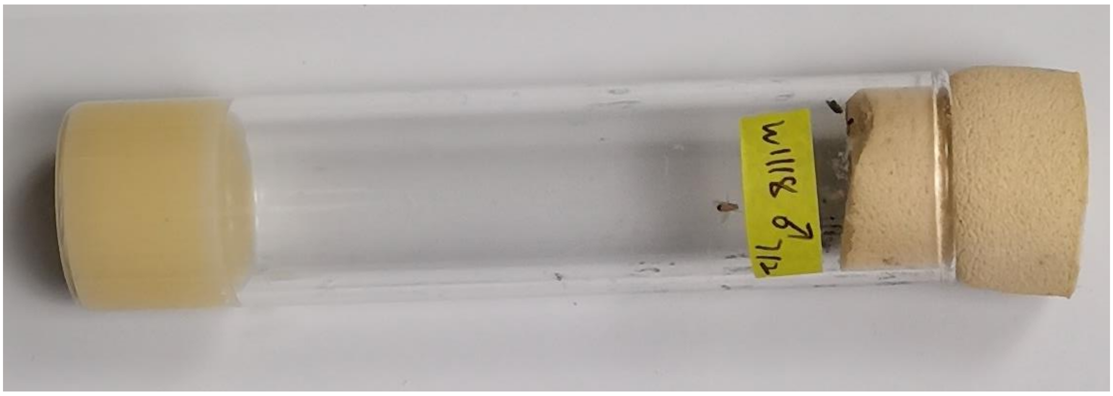

Fig 9, prepared fly vial

4.1.3. Drosophila behavior lab room preparation

The lab room should be maintained at 25°C, 50-60% humidity or be consistent with the Drosophila Incubator.

4.1.4. Camera setup

The recording devices should be setup before start the drosophila transference. PotPlayer is used to record the video. After the camera are connected to the computer, open PotPlayer in the computer.

Right-click on the PotPlayer, go to **open > Device settings…** (Fig 10, A). In Device Setting panel, choose the **Device** to the connected camera (Fig 10, B) and set the **Format** to MJPG 1024x768(p 4:3) (Fig 10, C). Click on the **Open device**, the PotPlayer will show the captured scene from camera.

Right-click on the PotPlayer, go to **Video > Video Recording > Record Video…** (Fig 10, D). Set the **Storage** file place and the **Filename prefix**. **Please avoid common illegal characters. Don't start**

or end you're the prefix with a space, period. Avoid using spaces or non-alphanumeric characters. Select **File Format** to MPEG4 MP4, check the **Use HQ Compression** and **Use buffering**. Set Bitrate to 50000 kbps. Now, PotPlayer is ready to record video.

According to the computer configuration, we may have 2-4 cameras connected to one computer. In this case, open several PotPlayer instances, each for one camera. Select corresponding camera in Device Setting panel and set different **Filename prefix** to distinguish the video recorded from different cameras.

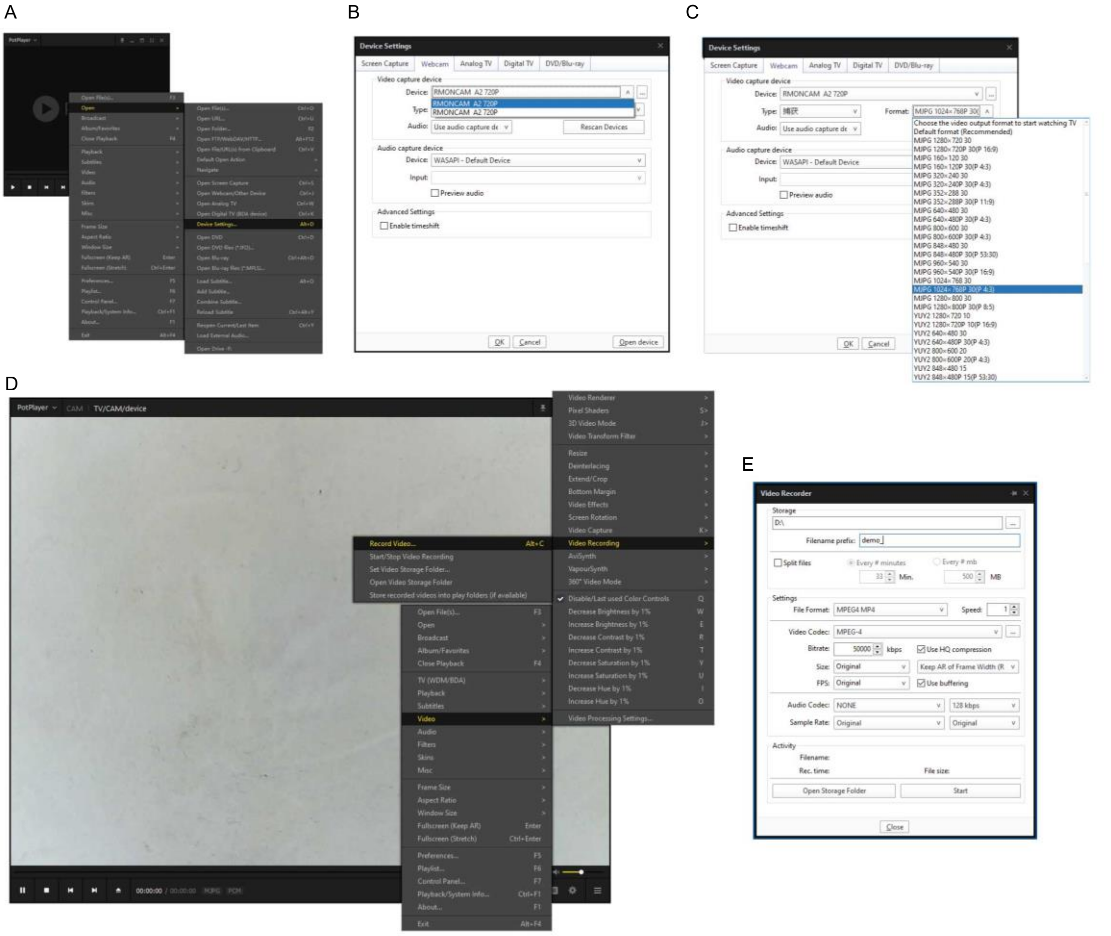

Fig 10, setup PotPlayer before recording. A, Device settings menu. B-C, Device settings panel. D, Record video menu. E, Video recorder panel.

4.1.5. Drosophila transference

Place the fly vials on the work surface of lab room before experiment for 1 hr. Before fly transference, take a quick check on the fly number in the vial. Insert the funnel into the chamber plug hole (fig 11, A). Put the vial into the opening part of the funnel and knock the funnel to allow flies to slip through the funnel and drop into the chamber (fig 11, B). When all flies have slipped into the chamber, re-plug the chamber acrylic plug and secure the bind chip. Peel off the label in the vial and tape it on the chamber corner. Mind that the label should not block the arena. If there's too many vials to transfer, two or more people might work together to complete this job. It is not wanted fly spends too much time in the chamber before the video recording.

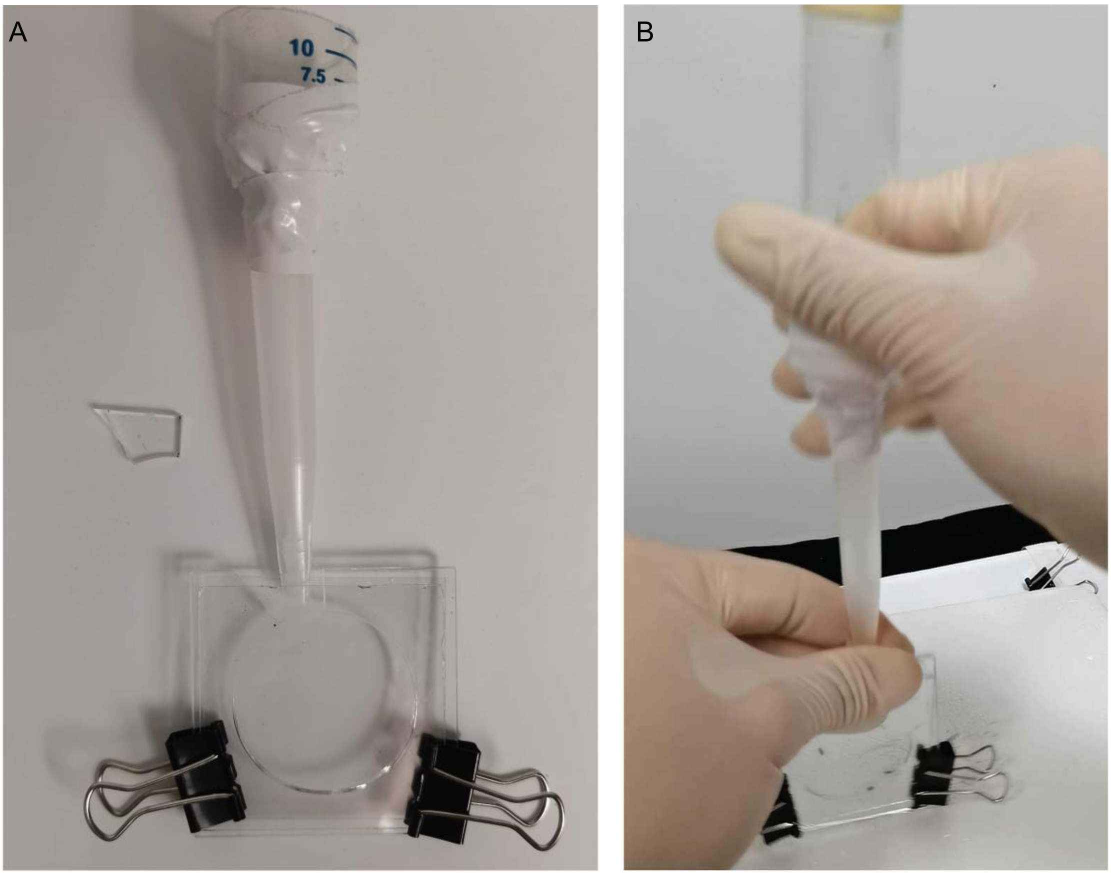

Fig 11, drosophila transference. A, Insert the funnel into the chamber. B, Knock down flies into the chamber.

4.1.6. Video recording

After all flies are transferred from vials to chambers, place the chamber into the camera brackets. Adjust the chambers to make the arena in the scene center. Fig 12 demonstrates qualified scene (fig 12, A) and unqualified ones (fig 12, B-F). The reason why the captured scene is unqualified includes improper lighting, incomplete arena or label etc. Check carefully before starting the video recording. Click the **Start** button in the **Video recorder** panel of PotPlayer in fig 10E, the recording is on. Start a timer and leave the lab room. When the timer finishes, click the **Stop** button in the **Video recorder** panel and stop recording. PotPlayer automatically name the recorded video as filename prefix + start point timestamp.

We recommend take 2 or 3 minutes longer recording than the video clip to be analyzed. Because the first 1min or 1.5min of video might be cut off due to background disturbance brought by setting up the chamber. The video length into analysis pipeline is better longer than 30 minutes. Hence, the recording time is better longer than 33minutes.

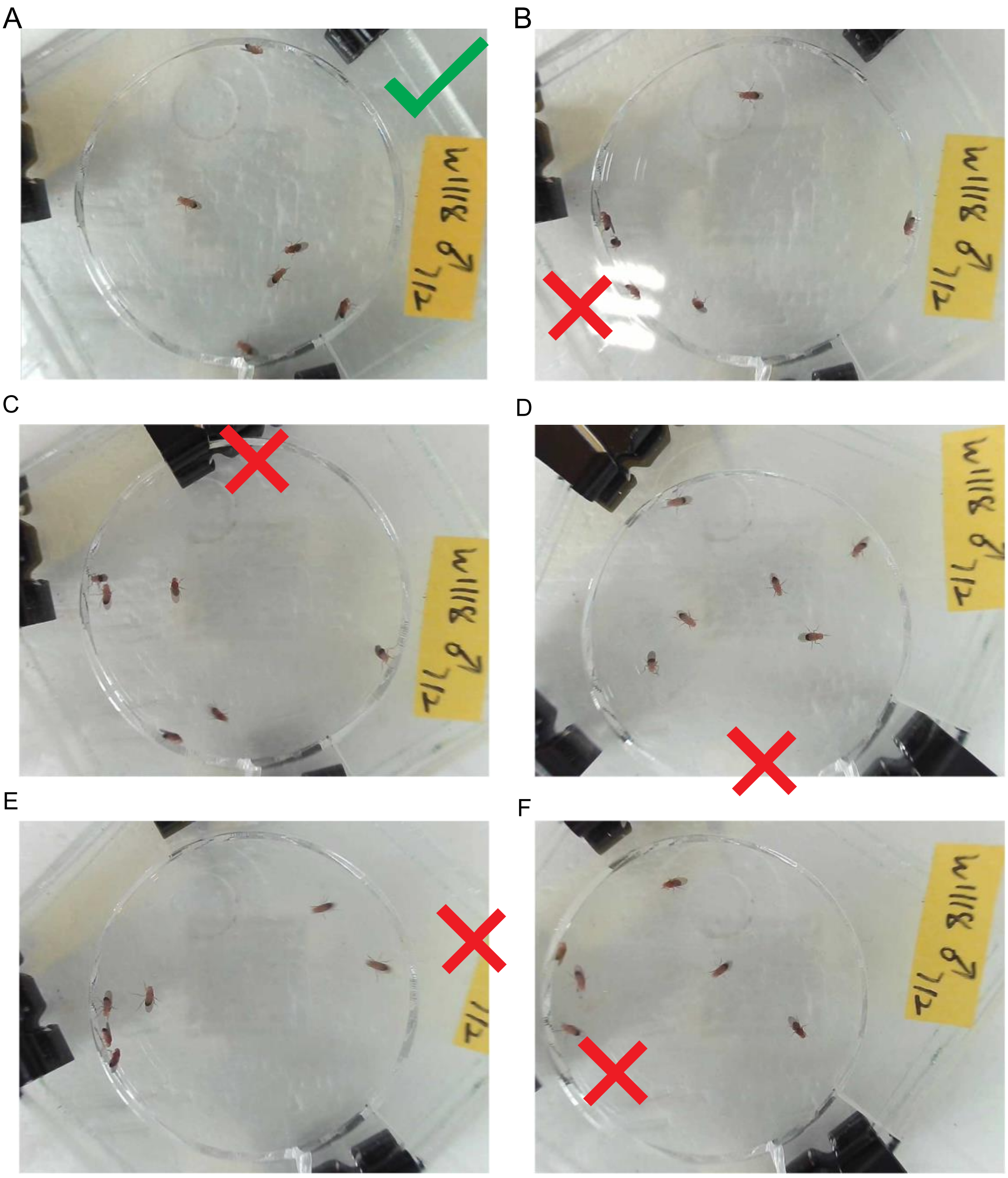

Fig 12, qualified and unqualified scenes. A, qualified scene. B, improper lighting. C, arena blocked by bind clips. D, incomplete arena. E, incomplete label. F, biased scene.

4.2. Data analysis by DVT desktop edition

4.2.1. Generate background image and clip video

DVT desktop edition runs on personal computer and all operations are graphical user interface supported. After video recording, double click **Videotrackanalysis.exe**, go to **Step 1**, click **Open video** to select the video to be analyzed. After the video is chosen, the software will show the frames, resolution and other information about the video. A captured screen is also shown in the panel. According to the image, the **Projectname** can be set. It is recommended using the experiment information as the project name. Mind that, DVT desktop edition handle video one by one. So, the project name is better to be numbered. **Please avoid common illegal characters in Porjectname and the video filepath. The space, period or other non-alphanumeric characters**

should also be avoided.

After Specifying the **Starttime** and **ENDtime**, Click the **Select Saveplace** button, browser the file folder to store the extracted background image and clipped video. If users want to clip 30-minutes-long video, set the **Starttime** and check **the 30mins video box**. Click run button, DVT will extract background image and clip video now (Fig 13). After the job finished, a message box will pop up to report its status.

The background image and clipped video will be name after the **Projectname** and stored at the **Saveplace** defined in the panel.

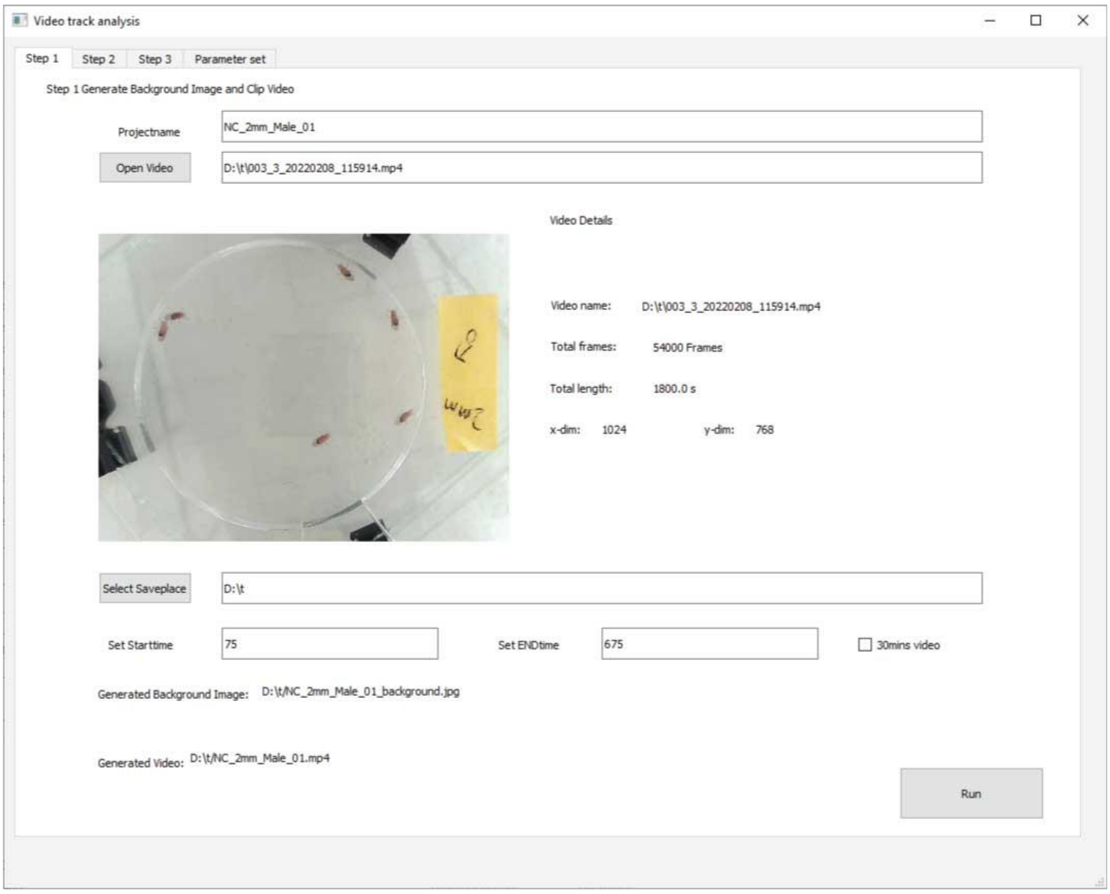

Fig 13, Generate background image and clip video in DVT desktop edition

4.2.2. Remove fly ghost in background images

DVT identify immobile pixels as video background (fig 14, A, B). Sometimes if the fly keeps motionless, it might be treated as background by mistake. In this case, the fly ghost in background images should be removed manually. Fig 14 C gives several fly ghost images as examples.

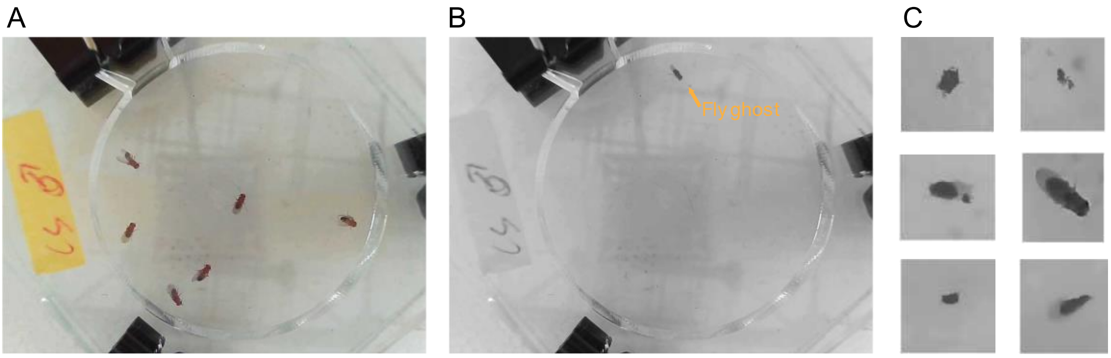

Fig 14, fly ghost. A, video screen capture. B, extracted background with fly ghost. C, examples on fly ghost.

Both Adobe photoshop and GIMP can be used to remove fly ghost in the image. In Adobe photoshop, use **Lasso tool** (fig15, A) to select the fly ghost and **Edit > Fill**(fig15, B)**,** by **Content-Aware** fill (fig15, C), the residual fly can be removed (fig15, D).

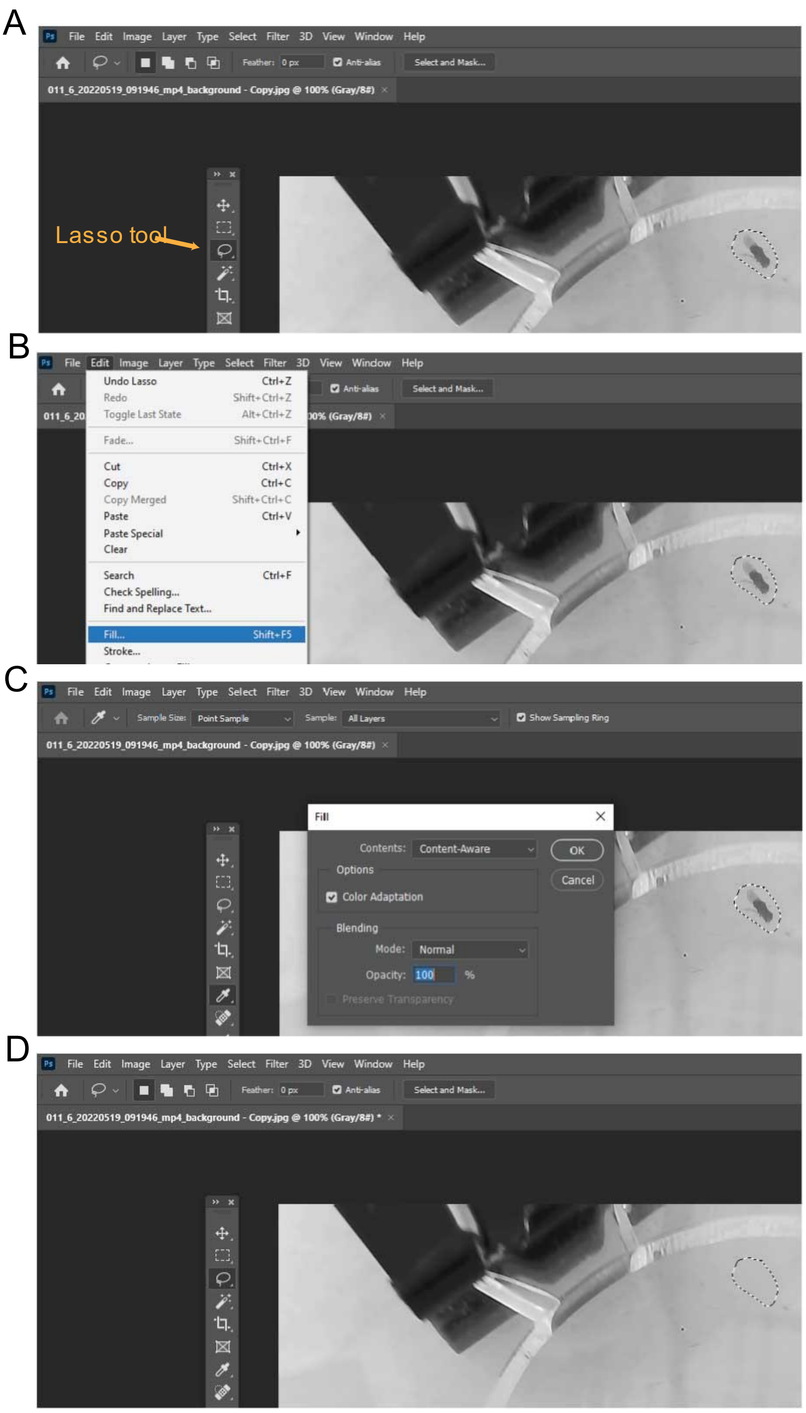

Fig 15, procedures of remove fly ghost by Adobe photoshop. A, Use Lasso tool to select the ghost. B, Go to Edit>Fill. C, Set to Content-Aware in the Fill panel, click OK. D, the ghost removal effect.

In GIMP, use **Free Selection tool** (fig16, A) to select the fly ghost and **Filters > Enhance > Heal selection** (fig16, B)**,** click **OK** at the popped panel (fig16, C), the residual fly can be removed

(fig16, D). The selection heal function is provided by GIMP plugin resynthesizer https://github.com/bootchk/resynthesizer.

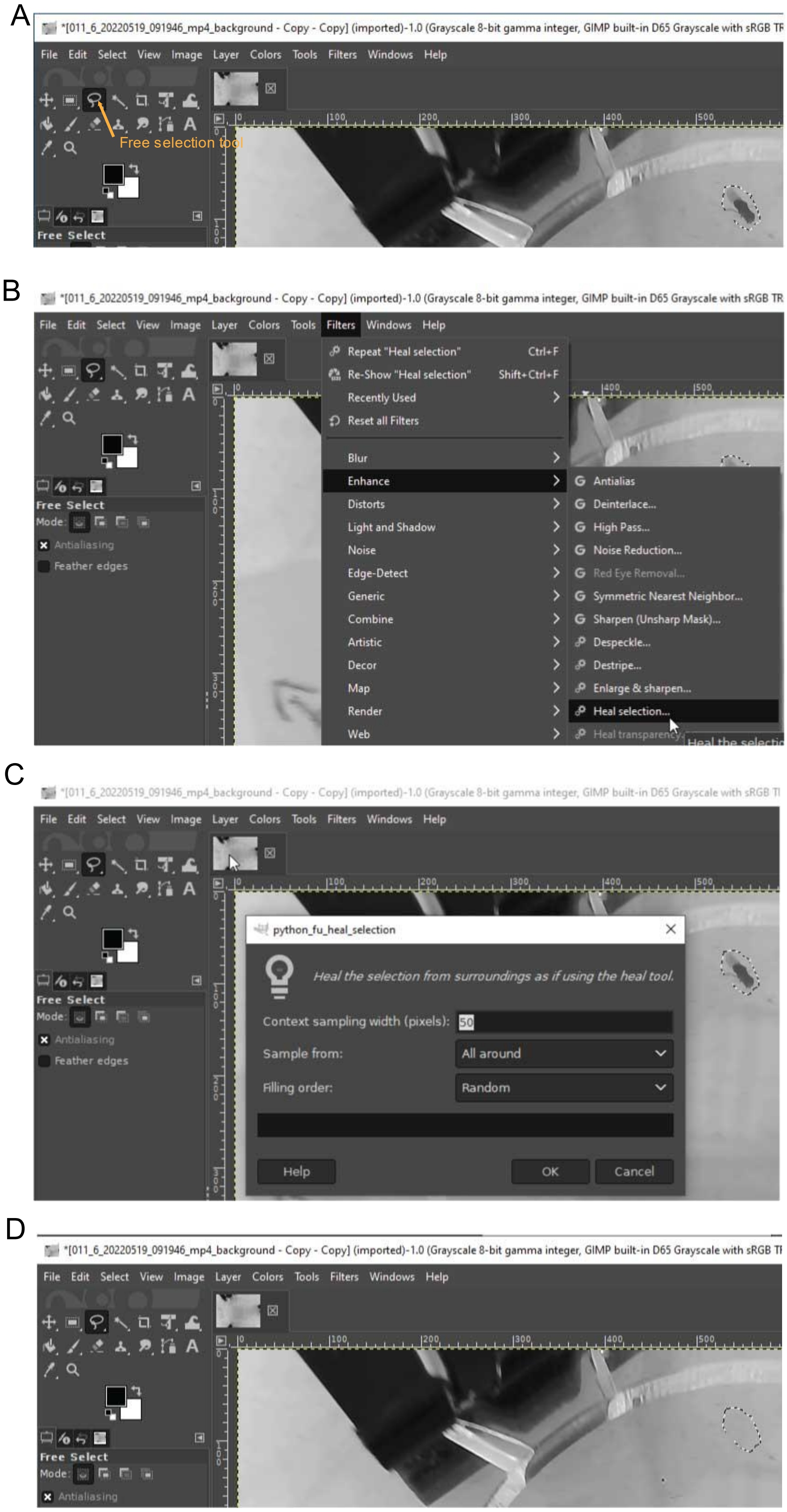

Fig 16, procedures of remove fly ghost by GIMP. A, Use Free selection tool to select the ghost.

B, Go to Filters>Enhance>Heal selection. C, Click OK at the popped panel. D, the ghost removal effect.

4.2.3. Arena identification and background removal

Step 2 is to identify arena and remove background from the video (Fig 17). Go to **Step 2**, **Open Image** to browser to the background image. The **Projectname** is recommended to follow the one in **Step 1**.

Select three points at the arena edge in the background image by single click. Then click **Compute Center of Circle**, the center of circle and radius is calculated. Click **Open ClippedVideo** to choose the clipped video outputted by step 1.

Click the **Select Saveplace** button, browser the file folder to store the video with background removal. Click run button to start the process.

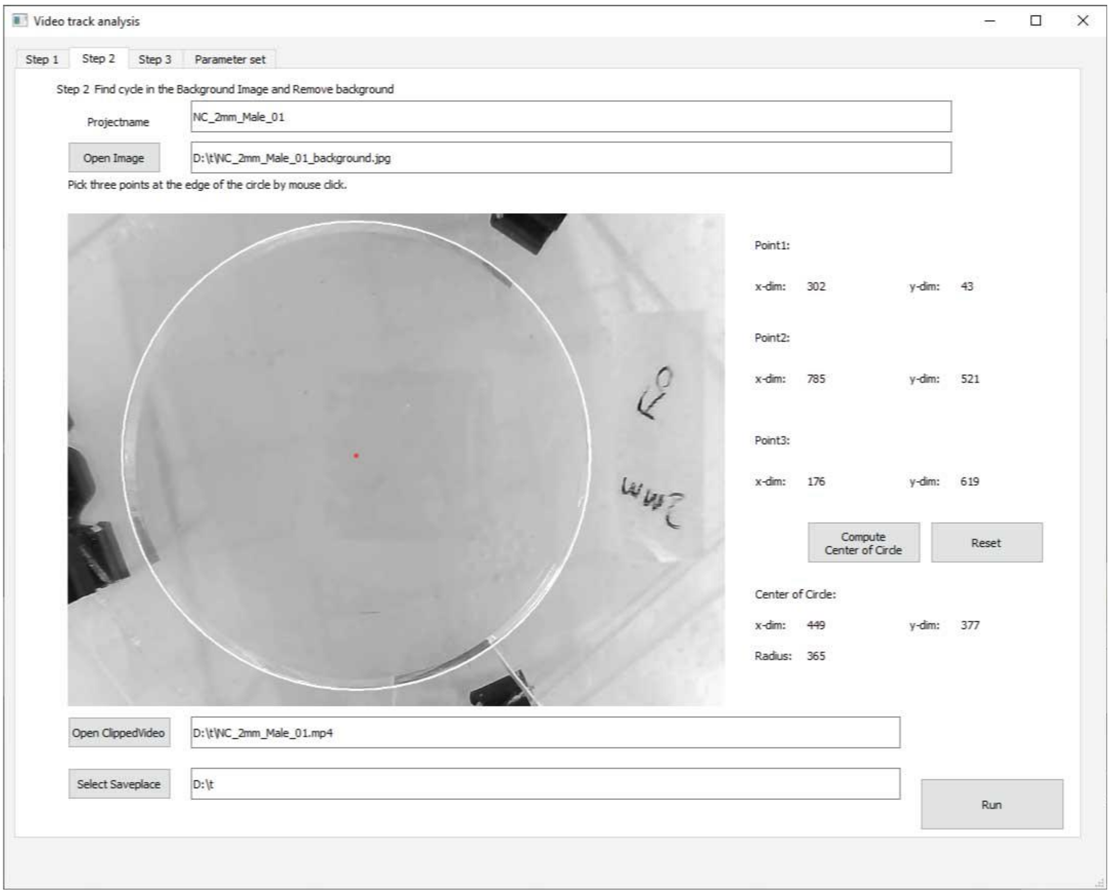

Fig 17, Arena identification and background removal

4.2.4. Detect fly trace

We recommend to use UMATracker to detect fly traces. Welcome to UMATracker's documentation! — UMATracker User's Guide (English) 1.0 ドキュメント gives a quick guide on UMATracker usage. Other trace detection software, Ctrax[1], ilastrk[2] might also perform this job well. If the trace is generated by other software, make sure the trace file has the same data definition as UMATracker.

Creating proper filter is always a tricky work in UMA tracking. For convivence, we provided two general filter at DVT/DVT desktop edition/release at main · Xingyinliu-Lab/DVT (github.com). The first filter, **general.filter** (fig 18, A), is suitable for almost all video generated by DVT. The second one, **general_complexed.filter** (fig 18, B), is more robust for noise in the video. of cause, the second

one is more computing stressful. Users can change the threshold according to the video brightness, etc. by **UMATracker-FilterGenerator**.

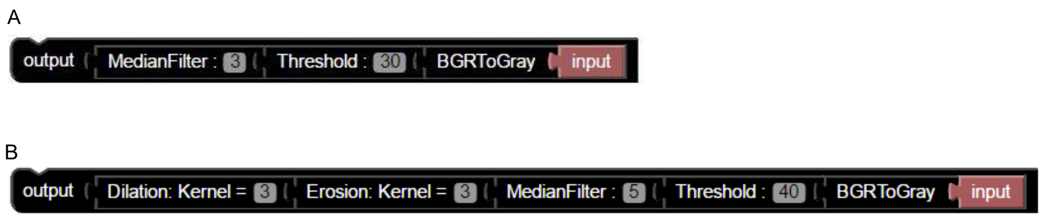

Fig 18, video filter in UMATracker. A, a general filter. B, a complexed but robust filter.

In the fly trace detection, open **UMATracker-Tracking.** Go to **Files > Open Video**, select a video in `/data/DVT/VT_dmeo_480p/`. Go to **Files > Open Filter**, select the **general.filter.** Set the **# of objects** and **# of k-means** to the fly number, hence 6 here, click **Set/Reset** button. Click the **PLAY** button and the program starts its work (Fig 19). After the tracing finished, save the fly trace results to `/data/DVT/VT_dmeo_480p/`. **Please do not rename the trace file.**

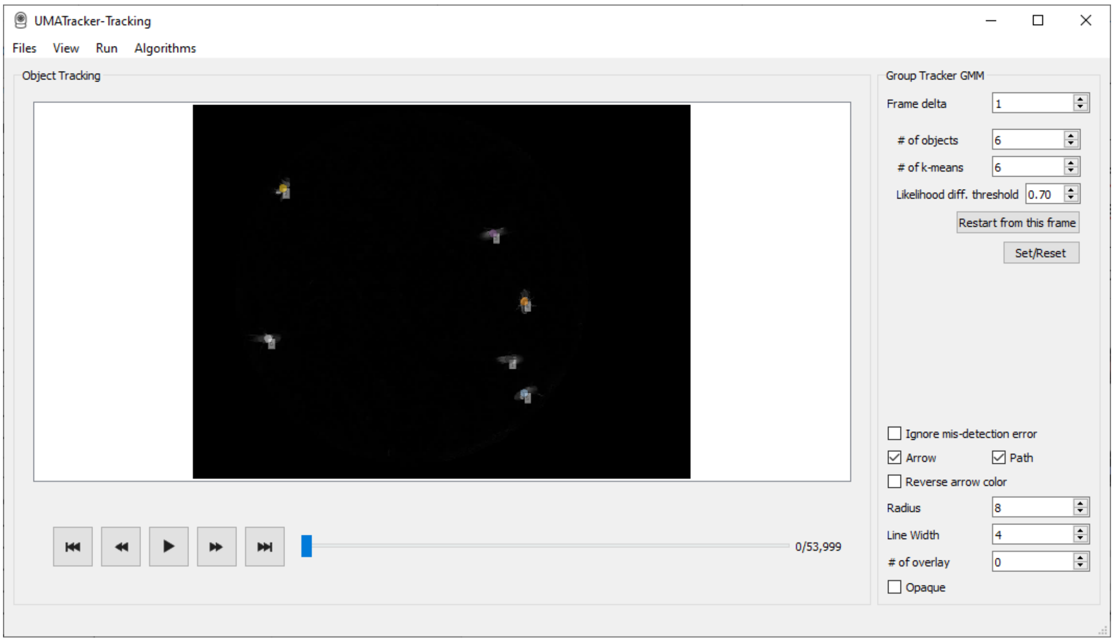

Fig 19, fly tracing by UMATracker-Tracking .

Another tip is to deal with multi videos, multi-instances of **UMATracker-Tracking** can be opened. In our practices, we build a MS windows virtual machine on KVM in our Linux server. It takes about 3 hours at Intel(R) Xeon(R) CPU E5-2683 v3 @ 2.00GHz with 48 UMATracker instance to trace 48 30minute-long videos simultaneously.

UMATracker provided **UMATracker-TrackingCorrector** for trace correction on reID and misdetection errors. If users fixed their trace file, please remember to modify the corresponding csv name column in the metadata.csv file. Generally speaking, if the chamber contains heterogeneous flies, that means flies with different genotypes, genders or undergone different treatments in one chamber, and the connections between the heterogeneity and different behavior pattern is interested, there's a need to make trace correction. Only after trace correction, it is meaningful to take individual fly behavior into statistical analysis.

Trace correction is such a labor-intensive work. We have proved the average-by-video

behavior before trace correction is equal to or expected equal to that after trace correction. That is to say, if the flies in one chamber are homogeneous, average-by-video behavior feature would be a better choice to pipped into the statistical analysis instead of trace correction.

4.2.5. Fly behavior analysis

After the fly trace is detected by UMATracker, now we go to Step 3 to analyze drosophila behavior (Fig 20). Set the **Projectname** and click **Open Trackfile** to choose the trace file generated by UMATracker. Click **Open ClippedVideo** to choose the clipped video outputted by step 1.

Input the number of drosophila, the center and the radius of circle. Click the **Check** button, a random frame in the video will be extracted and the corresponding fly position detected by UMATracker will be shown in the frame image. Take a quick check and make sure the trace file is the right one. Click the **Select Saveplace** button, browser the file folder to store the analysis result. If annotated video is wanted, check the box at bottom. Click run button to start the process.

Note that during analysis process (step 1, 2, 3 included), the DVT desktop edition may lose responding. The software will recover itself until the analysis finishes. So, do not force quit the program (Fig 20).

The DVT desktop software does not support batch analysis for fly behaviors. If users have multiple videos to analysis, the output results by DVT desktop software have to be integrated manually.

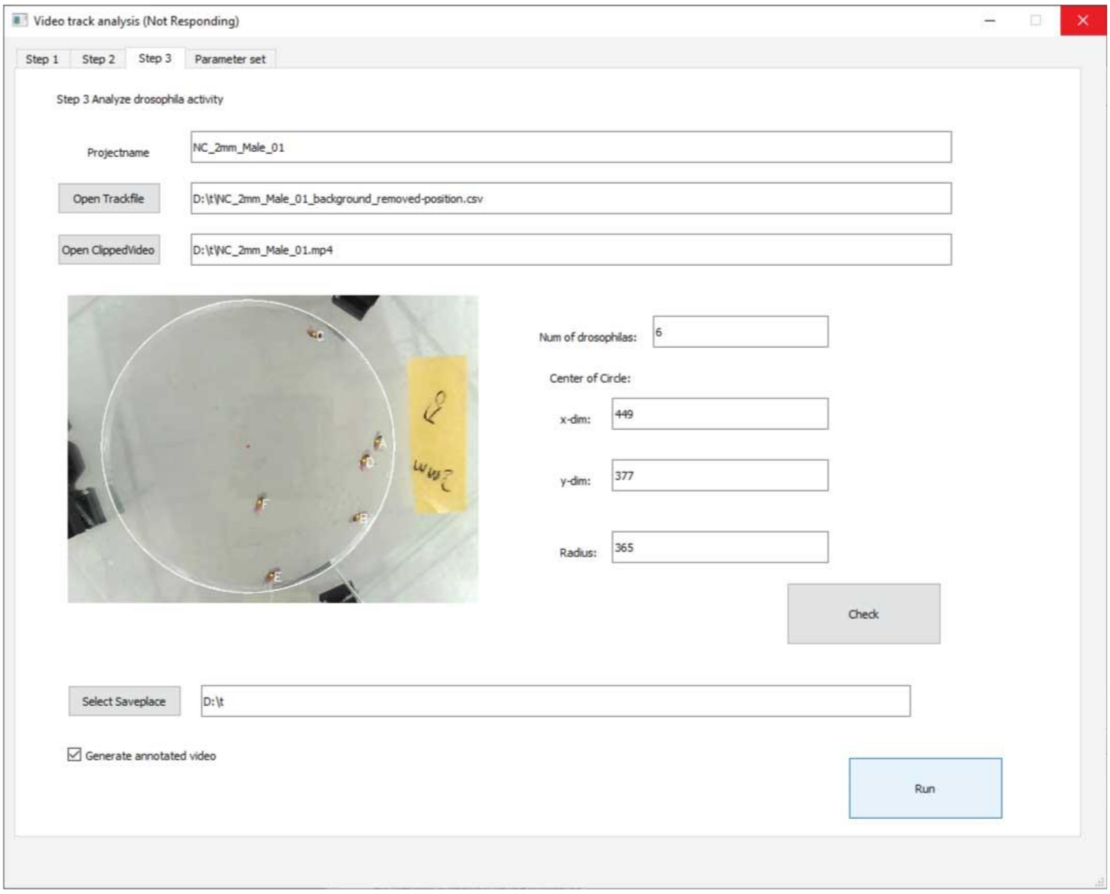

Fig 20, Fly behavior analysis

4.3. Data analysis by DVT server edition

4.3.1. Transfer recorded video to the server

Transfer video files to the Linux server by portable driver or *scp* command. **Please avoid**

common illegal filename and directory characters. Don't start or end your filename with a space, period. Avoid using spaces or non-alphanumeric characters. Let's suppose the recorded video is stored at `/data/DVT/VT_dmeo_raw/`. Do not put irrelevant files at `/data/DVT/VT_dmeo_raw/`. `Make sure you have full access to /data/DVT/`. Sharing the file folder `/data/DVT/` between the server and windows pc client is recommended because there's multi operations on images and videos. Researchers can also use xRDP to remote the server. In this case, no folder sharing is needed. Let's suppose the recorded video is stored at `/data/DVT/VT_dmeo_raw/`.

4.3.2. Clip the video and extract background images

Usually, the first 1minute or 1.5minute of video is to be cut off because there might be background disturbance. The end point is determined by the start point plus the video length to be analyzed. The s99.1_batchscript.py performs this function. A usage demo is shown below.

```
cd Drosophila_track_DVT/
python s99.1_batchscript.py 48 /data/DVT/VT_dmeo_raw/ /data/DVT/VT_dmeo_clip/ 75 1875
```

The script takes following arguments sequentially.

```
1. processors: Number of computation threads to use. The number of threads should be lesser or equal to the number of available CPU cores. In the demo case, it is 48.
2. rawvideo_fileplace: fileplace where stores the recorded video. In the demo case, it is /data/DVT/VT_dmeo_raw/.
3. clipedvideo_fileplace: fileplace to store the cliped video. In the demo case, it is /data/DVT/VT_dmeo_clip/.
4. clipedvideo_start: start point of video clipping (in seconds). In the demo case, it is 75.
5. clipedvideo_end: end point of video clipping (in seconds). In the demo case, it is 1875.
```

The demo script cut the videos in `/data/DVT/VT_dmeo_raw/` from 75s to 1875s, that is exactly 30 minutes, and stores the clipped video to `/data/DVT/VT_dmeo_clip/`. This script also generate a drafted metadata.csv lacking genotype, sex and other required information. It takes about 2-3 hours at Intel(R) Xeon(R) CPU E5-2683 v3 @ 2.00GHz with processors = 48 to clip 48 videos to 30minute ones. Check the image and if fly ghost exists, remove the residual ghost following section 4.2.2 instructions.

4.3.3. Collect meta info

In step 2 a draft meta file is generated. Before proceeding to the next step, double click **DVT_metahelper.exe**, this software will help to complete the meta information required.

Copy the draft metadata.csv and all background images to windows client. In our practices, we mapped the server folder as one client local driver. Click **Select imageplace**, browser to the folder where stores the background images. Click **Open metadata**, browser to the draft metadata.csv file. If all setup, the first background image should be shown in the left-bottom corner of the software (Fig 21, A).

The first thing is to identify the arena cycle. Select three points at the arena edge in the background image by single click as dispersed as possible (fig 21, B). If not satisfied, click **Remove point** button at the left panel. After point selection, you can click **Check** button to have quick look at the arena cycle identified (fig 21, C).

Other meta info to be completed including the number, genotype, gender of flies in the arena, experiment condition and replicate information. Fill this information at the right-bottom corner panel.

As all information are complete, the **Next** button can proceed to the next video. Historical information about the genotype, number, replicate is shown in the below columns. Users can choose the proper one by single click (fig 21, D).

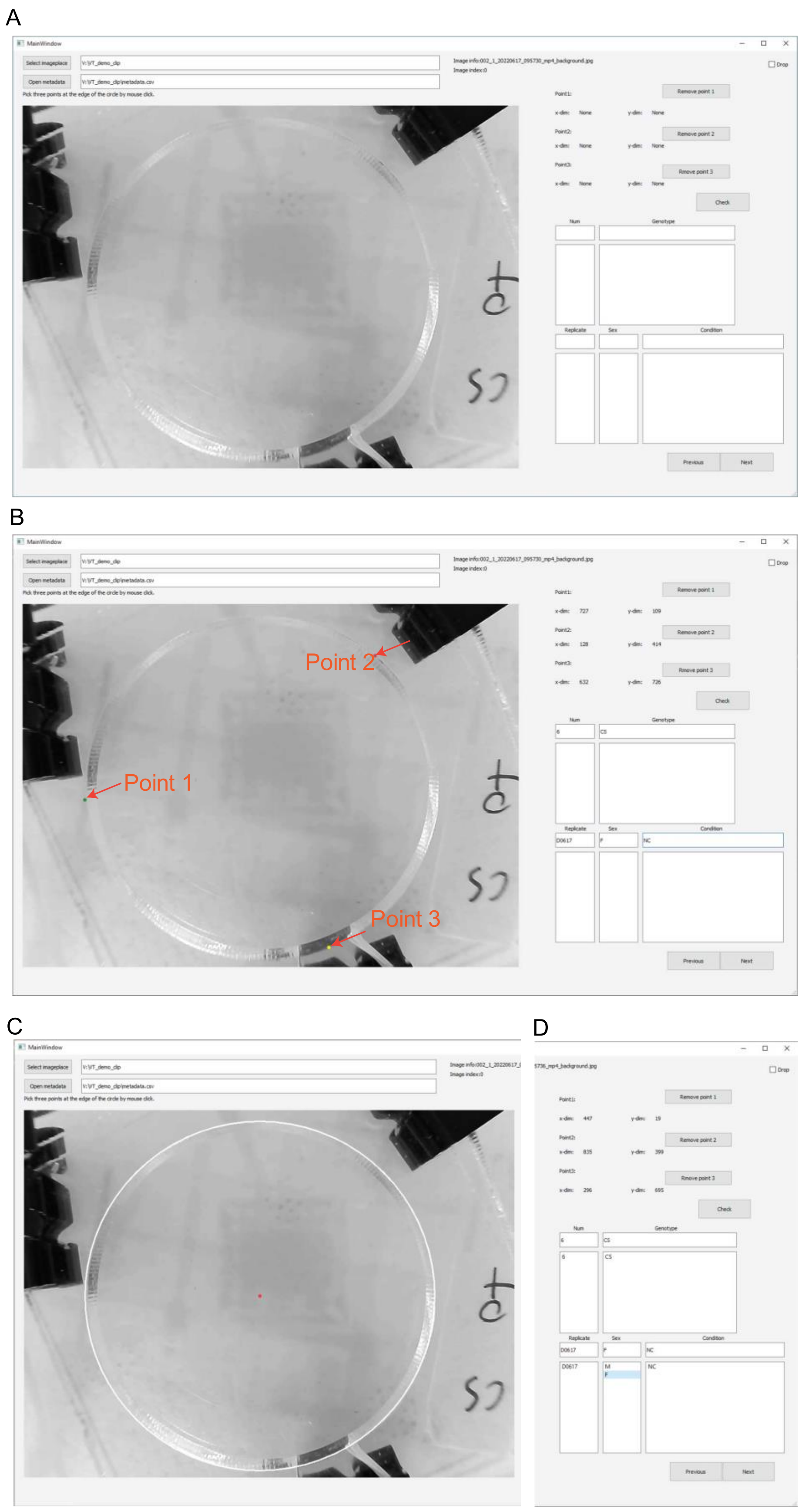

Fig 21, DVT metahelper usage. A, Main graphic user interface of metahelper. B, completion of the meta info of video. C, arena identification check. D, autocomplete function of metahelper.

4.3.4. Remove background and scale the video

To avoid disturbance on the trace detection, in this step the background is removed from the video. Fig 22 shows the comparison between video screen before and after the background removal (Fig 22).

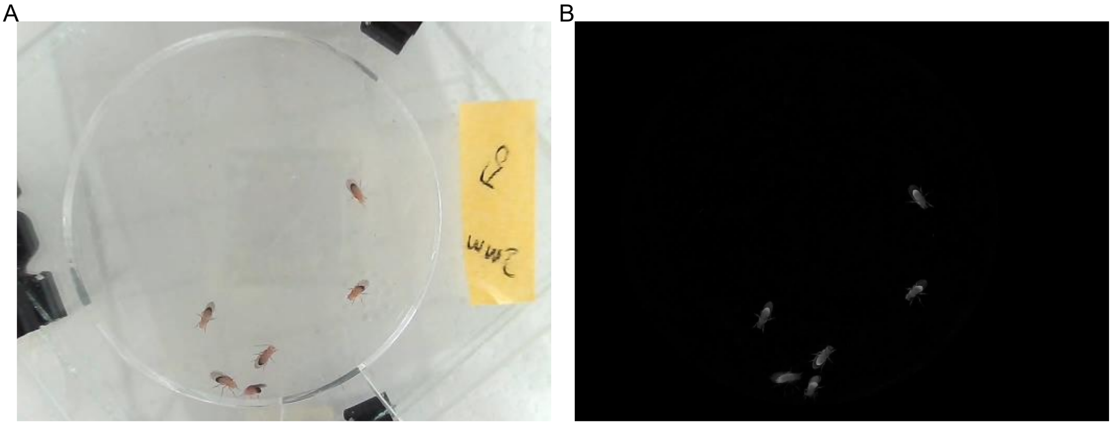

Fig 22, background removal. A, origin video screen. B, background removed video screen.

The s99.2_batchscript.py performs this function. A usage demo is shown below.

```
cd Drosophila_track_DVT/
python s99.2_batchscript.py 48 /data/DVT/VT_dmeo_clip/ /data/DVT/VT_dmeo_clean/ /data/DVT/VT_dmeo_480p/
```

The script takes following arguments sequentially.

```
1. processors: Number of computation threads to use. The number of threads should be lesser or equal to the number of available CPU cores.
2. clipedvideo_fileplace: fileplace where stores the cliped video
3. cleaneddvideo_fileplace: fileplace to store the video with background removed
4. video480p_fileplace: fileplace to store the scaled video
```

The demo script removes background of the videos in `/data/DVT/VT_dmeo_clip/`, and stores the generated videos to `/data/DVT/VT_dmeo_clean/`. Then the videos are scaled to 640x480p in order to reduce the computing stress in fly trace detection. The scaled video are stored in `/data/DVT/VT_dmeo_480p/`. It takes about 3 hours at Intel(R) Xeon(R) CPU E5-2683 v3 @ 2.00GHz with processors = 48 to remove background and scale 48 30-minutes videos. After background removal, use UMATracker to detect fly traces as described in section 4.2.4.

4.3.5. Fly behavior analysis and visualization

When all trace files are settled, it's time to analyze the behaviors of flies. The s99.4_batchscript.py performs this function. A usage demo is shown below.

```
cd Drosophila_track_DVT/
python s99.4_batchscript.py 48 /data/DVT/VT_dmeo_480p/ 0 0
```

If users want the video annotated, try:

```
cd Drosophila_track_DVT/
python s99.4_batchscript.py 48 /data/DVT/VT_dmeo_480p/ 0 1 /data/DVT/VT_dmeo_annotated/
```

The script takes following arguments sequentially.

```
1. processors: Number of computation threads to use. The number of threa
   ds should be lesser or equal to the number of available CPU cores.
2. fileplace: fileplace where stores the fly trace data
3. prefix: 1 for analyze the frist 10 minutes of trace. 2 for analyze th
   e frist 20 minutes of trace. 3 for analyze the frist 20 minutes of tr
   ace. 0 for analyze the whole trace file.
4. annotated: 1 for marking the id and historical trace on drosophila in
    the video. 0 for no marking.
5. annotated_video_place: fileplace to store the annotated video. If ann
   otated is set to 1, this parameter should be provided.
```

The analysis results can be found at `/data/DVT/VT_dmeo_480p/analysis + prefix/`, in this case, `/data/DVT/VT_dmeo_480p/analysis0/`. A detailed information about the analysis result and its interpretation can be found at the following section 4.4. It takes about 5 hours at Intel(R) Xeon(R) CPU E5-2683 v3 @ 2.00GHz with processors = 48 to analysis fly behavior of 48 30-minutes traces.

4.4. Results interpretation

4.4.1. Fly behavior features definition

DVT outputs about 70 fly behavior features. Following gives an expounded definition on each behavior feature.

1. Exploration efficiency by time

$$\text{Exploration efficiency by time} = \frac{\text{The time used to complete 80\% of the arena exploration}}{\text{Video length}}$$

2. Area explored by given time

$$\text{Area explored by given time} = \frac{\text{The arena exploration in a given time (10 minutes)}}{\text{The arena area}}$$

3. Exploration efficiency by travel length

$$\text{Exploration efficiency by travel length} = \frac{0.8}{\text{Fly move length during the exploration of 80\% of the arena}}$$

4. Avg. velocity[1] (mm/s).

$$\text{Avg. velocity} = \frac{\text{Fly move length}}{\text{Fly move time}}$$

[1] Note 1: the speed was below the lower threshold (0.5 mm/s) the fly was classified as at rest or inactive. Fly movement, including, velocity, move length, turning angular etc. was not taken into account.

5. Avg. velocity at arena edge[1,2] (mm/s).

$$\text{Avg. velocity at arena edge} = \frac{\text{Fly move length at arena edge}}{\text{Fly move time at arena edge}}$$

[2] Note 2: 3mm to center from the circumference was considered as the arena edge. The remained arena was arena centre.

6. Avg. velocity at centre[1,2](mm/s).

$$\text{Avg. velocity at arena centre} = \frac{\text{Fly move length at arena centre}}{\text{Fly move time at arena centre}}$$

7. Max. velocity[1,3]. 95th percentile of fly moving velocity (mm/s).

[3] Note 3: we take the 95th percentile of features as the maximum value in order to avoid

misleading results brought out by reID or mis-detection error.

8. Max. velocity at arena edge[1,3]. 95th percentile of fly moving velocity at arena edge (mm/s).
9. Max. velocity at centre[1]. 95th percentile of fly moving velocity at arena centre (mm/s).
10. Total move length[1]. Fly total move length(mm).
11. Move length at arena edge. Fly move length when moving at arena edge. (mm)
12. Move length at arena centre. Fly move length when moving at arena centre. (mm)
13. Total move time[1].

$$\text{Total move time} = \frac{\text{Fly move time}}{\text{Video length}}$$

14. Move time prop. at arena edge.

$$\text{Move time prop. at arena edge} = \frac{\text{Fly move time at arena edge}}{\text{Total time spent at arena edge}}$$

15. Move time prop. at arena centre

$$\text{Move time prop. at arena centre} = \frac{\text{Fly move time at arena centre}}{\text{Total time spent at arena centre}}$$

16. Avg. distance from the arena centre

$$\text{Avg. distance from the arena centre} = \frac{\text{Avg. distance from fly position to the centre}}{\text{Arena radius}}$$

17. Time prop. spent at edge

$$\text{Time prop. spent at edge} = \frac{\text{Total time spent at arena edge}}{\text{Video length}}$$

18. Movelength ratio at edge

$$\text{Movelength ratio at edge} = \frac{\text{Fly move length at arena edge}}{\text{Total move length}}$$

19. Tracks number[4]. Counts of tracks.

[4] Note 4: A track is defined as the path treaded by a fly when it moves continuously. This concept is inherited from Aggarwal's work [3].

20. Avg. track duration. Average time duration of each track (s).
21. Avg. track length. Average path length of each track (mm).
22. Long stop episodes number[5]. Counts of long stop episodes.

[5] Note 5: Inactivity with duration longer than 2s is defined as long stop episodes.

23. Avg. inactivity duration. Average time duration of each inactivity episode (s).
24. Avg. long stop episodes duration. Average time duration of each long-stop episode (s).
25. Avg. track straightness[6]. Average straightness of track path.

[6]Note 6: The track straightness is the coefficient of determination, r^2 value, of the linear regression model of the fly position in the time windows. The time window was set to 1s. This concept is inherited from Aggarwal's work [3].

26. Track straightness at arena centre[6]. Average straightness of track path when fly moves at the arena centre.
27. Track straightness at arena edge[6]. Average straightness of track path when fly moves at the arena edge.
28. Avg. angular velocity[7]. Average angular velocity when fly is being moving (rad/s).

$$\text{Avg. angular velocity} = \text{Average}\left(\frac{\text{Fly turning angle in the time window}}{\text{Time window size}}\right)$$

[7] Note 7: the calculation on angular velocity, and meander, the time window was set to 0.2s to be consistent with Martin's work [4].

29. Avg. angular velocity at arena centre. Average angular velocity when fly is being moving at arena centre (rad/s).
30. Avg. angular velocity at arena edge. Average angular velocity when fly is being moving at arena edge (rad/s).
31. Max. angular velocity[3]. 95th percentile of fly angular velocity(rad/s).
32. Max. angular velocity at arena centre[3]. 95th percentile of fly angular velocity at arena centre(rad/s).
33. Max. angular velocity at arena edge[3]. 95th percentile of fly angular velocity at arena edge(rad/s).
34. Avg. meander (rad/mm)[7].

$$\text{Avg. meander} = \text{Average}\left(\frac{\text{Fly turning angle in the time window}}{\text{Fly move length in the time window}}\right)$$

35. Avg. meander at centre (rad/mm). Average meander when fly is being moving at arena centre.
36. Avg. meander at edge (rad/mm). Average meander when fly is being moving at arena edge.
37. Max. meander (rad/mm)[3]. 95th percentile of fly meander.
38. Max. meander at centre (rad/mm)[3]. 95th percentile of fly meander at arena centre.
39. Max. meander at arena edge (rad/mm)[3]. 95th percentile of fly meander at arena edge.
40. Acquaintance.

$$\text{Acquaintance} = \frac{\text{The max interaction duration with other flies}}{\text{Total interaction duration with other flies}} \Bigg/ \frac{1}{\text{number of flies in arena} - 1}$$

41. Social space distance. Averaged the distance to the closest neighbor by frame (mm). This concepts comes from McNeil's work [5]. An improvement in DVT is DVT calculate the space distance every frame in the video and take average of all frames. While McNeil's work took one photo 30 minutes after flies were transferred to the chamber and calculate the distance according to that photo.
42. Space distance at arena edge. Averaged the distance to the closest neighbor by frame when fly locates at the arena edge (mm).
43. Space distance at arena centre. Averaged the distance to the closest neighbor by frame when fly locates at the arena centre (mm).
44. Space distance at activity episodes. Averaged the distance to the closest neighbor by frame when fly is being moving (mm).
45. Space distance at inactivity episodes. Averaged the distance to the closest neighbor by frame when fly is being inactive(mm).
46. SSI[8]. In fly SSI (social space index) calculation, the distances between the fly and other fly in every frame was binned by 5mm. The SSI is subtracting the percentage of flies in the 2nd bin from the percentage of flies in the 1st bin.

[8], Note 8: This concept comes from Simon's work [6].

47. SSI at arena edge. Fly social space index when fly locates at the arena edge.
48. SSI at arena centre. Fly social space index when fly locates at the arena centre.

49. SSI at activity episodes. Fly social space index when fly is being moving.
50. SSI at inactivity episodes. Fly social space index when fly is being inactive.
51. Total interaction duration[9].

$$\text{Total interaction duration} = \frac{\sum \text{Fly interaction time with other flies}}{\text{Video length}}$$

9, Note 9: Interaction with different flies at the same time was treated as different interaction. The total interaction duration might be larger than 100% because there's a chance fly interacts with two or more flies at the same time.

52. Interaction duration at edge.

$$\text{Interaction duration at edge} = \frac{\sum \text{Fly interaction time with other flies when fly locates at the arena edge}}{\text{Video length}}$$

53. Interaction time prop. at edge

$$\text{Interaction time prop. at edge} = \frac{\sum \text{Fly interaction time with other flies when fly locates at the arena edge}}{\text{Total time spent at arena edge}}$$

54. Interaction duration at centre

$$\text{Interaction duration at centre} = \frac{\sum \text{Fly interaction time with other flies when fly locates at the arena centre}}{\text{Video length}}$$

55. Interaction time prop. at centre

$$\text{Interaction time prop. at centre} = \frac{\sum \text{Fly interaction time with other flies when fly locates at the arena centre}}{\text{Total time spent at arena centre}}$$

56. Interaction duration at activity episodes.

$$\text{Interaction duration at activity episodes} = \frac{\sum \text{Fly interaction time with other flies when fly is being moving}}{\text{Video length}}$$

57. Interaction time prop. at activity episodes

$$\text{Interaction time prop. at activity episodes} = \frac{\sum \text{Fly interaction time with other flies when fly is being moving}}{\text{Total move time}}$$

58. Interaction duration at inactivity episodes

$$\text{Interaction duration at inactivity episodes} = \frac{\sum \text{Fly interaction time with other flies when fly is being inactive}}{\text{Video length}}$$

59. Interaction time prop. at inactivity episodes

$$\text{Interaction time prop. at inactivity episodes} = \frac{\sum \text{Fly interaction time with other flies when fly is being inactive}}{\text{Total move time}}$$

60. Total interaction duration at long-stop

$$\text{Interaction duration at long} - \text{stop} = \frac{\sum \text{Fly interaction time with other flies when fly is at long stop episodes}}{\text{Video length}}$$

61. Interaction time prop. at long-stop

$$\text{Interaction time prop. at long} - \text{stop} = \frac{\sum \text{Fly interaction time with other flies when fly is at long stop episodes}}{\text{Duration time of all long stop episodes}}$$

62. Interaction episode count. Number of Interaction episodes.
63. Interaction episode duration (s). Average duration of fly interaction episode.
64. Avg. number of crowded dro. Average number of flies interact with the same fly simultaneously.
65. Degree assortativity coefficient[10].

Assortativity measures the similarity of connections in the graph with respect to the node degree. The degree assortativity in a network is defined as the Pearson correlation coefficient of the degree of connected nodes, measured over the set of all edges.

[10] Note 10: In DVT, the social network topology features including Degree assortativity coefficient, Clustering coefficient, Betweenness centrality, Network diameter, Network degree, Unconnected social network prop., Global efficiency, Closeness centrality, Eccentricity and Dominating is calculated and averaged for every iterative network using a moving-social-network-window. The moving-social-network-window represents 50% of the total number of interactions possible for flies. For example, the moving-social-network-window is 8 for a 6-fly social community in the chamber. DVT calculates the network features for the social network composed by the first 8 interactions and then the second network by the 9th to 16th interactions. For 8-fly community, the moving-social-network-window is 14. This calculation procedures are inherited from Schneider's work [7]. Definition about these features was quoted from Degree assortativity (konect.cc) and Software for Complex Networks — NetworkX 2.8.4 documentation. Most calculation of the features are supported by NetworkX in python.

66. Clustering coefficient.

Clustering coefficient of a node in social network is the fraction of possible triangles through that node that exist.

67. Betweenness centrality

Betweenness centrality of a node is the sum of the fraction of all-pairs shortest paths that pass through the node.

68. Network diameter

The diameter is the maximum eccentricity.

69. Network degree.

Degrees of nodes in the fly social network.

70. Unconnected social network prop.

The unconnected social network proportion in all iterative networks separated by the moving-social-network-window.

71. Global efficiency

The efficiency of a pair of nodes in a graph is the multiplicative inverse of the shortest path distance between the nodes. The global efficiency of a graph is the average efficiency of all pairs of nodes.

72. Closeness centrality[11]

Closeness centrality of a node is the reciprocal of the average shortest path distance to the node over all reachable nodes.

[11] Note 11: Closeness centrality, Eccentricity and Dominating is available for fly individuals.

73. Eccentricity

The eccentricity of a node is the maximum distance from the node to all other nodes in the social network.

74. Dominating

A dominating set for social network with node set V is a subset D of V such that every node not in D is adjacent to at least one member of D.

4.4.2. Analysis data generated by DVT

There're slightly differences in the generated analysis data between DVT server edition and desktop edition.

For DVT server edition, all results are stored at `/data/DVT/VT_dmeo_clip/`, `/data/DVT/VT_dmeo_clean/`, and `/data/DVT/VT_dmeo_480p/` three folder.

`/data/DVT/VT_dmeo_clip/` includes:

- The clipped video.
- Extracted background image, *_background.jpg.
- metadata.csv, drafted meta info file with no genotype or other information.
- Capture image (the 100th frame) from video, stored in N_img/.
- Extracted images for fly body size measure, stored in img_for_body_size_measure/.

`/data/DVT/VT_dmeo_clean/` includes:

- Video with background removal

`/data/DVT/VT_dmeo_480p/` includes:

- Scaled video with background removal
- Fly trace files
    - *.ffindex
    - *-colors.txt
    - *-info.txt
    - *-position.csv, fly position at every frame of the video.
- metadata.csv with completed meta info
- Fly trace analysis folder
    - Summarized Visualization
        - motion_analysis_plot.pdf. Grouped boxplot or violin plot for fly locomotion behaviors. Fig 23 makes demonstration on this file.

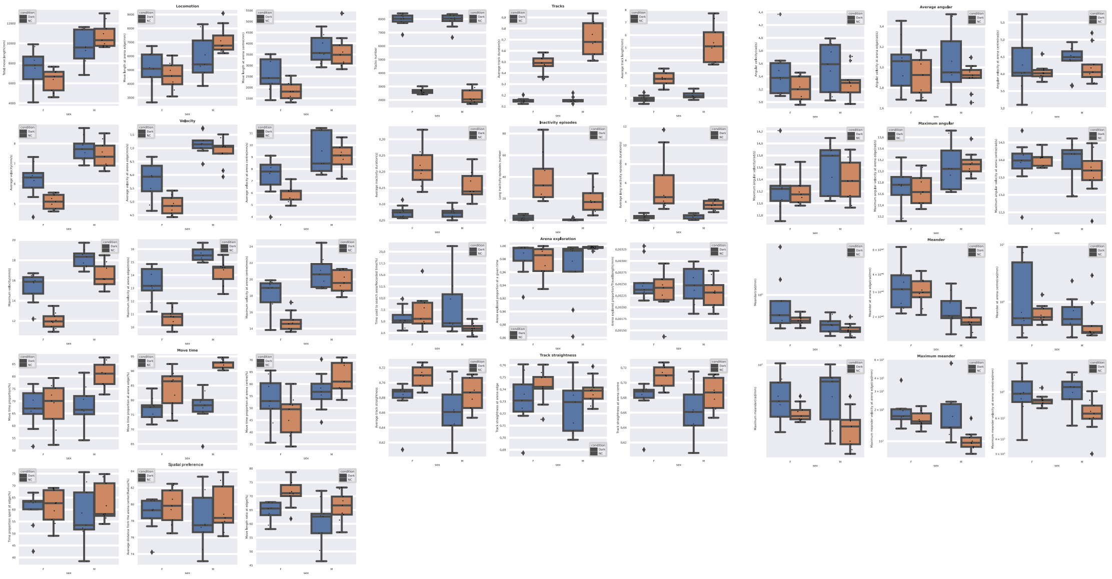

Fig 23, Grouped locomotion behavior boxplot demo. The demo showed fly locomotion difference between under normal lighting and under dark mode.

- interaction_analysis_plot.pdf. Grouped boxplot or violin plot for fly social behaviors. Fig 24 makes demonstration on this file.

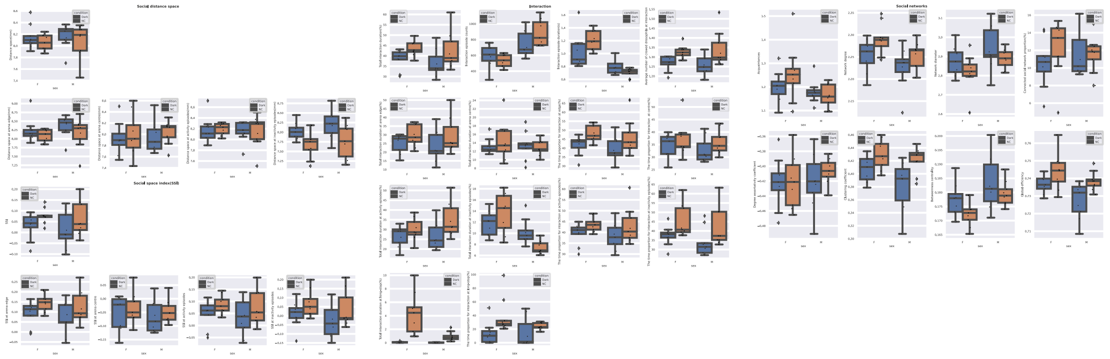

Fig 24, Grouped social behavior boxplot demo. The demo showed fly social behavior difference between under normal lighting and under dark mode.

- motion_analysis_sliced_plot.pdf. Grouped timeseries plot of drosophila locomotion behavior. Fig 25 makes demonstration on this file.

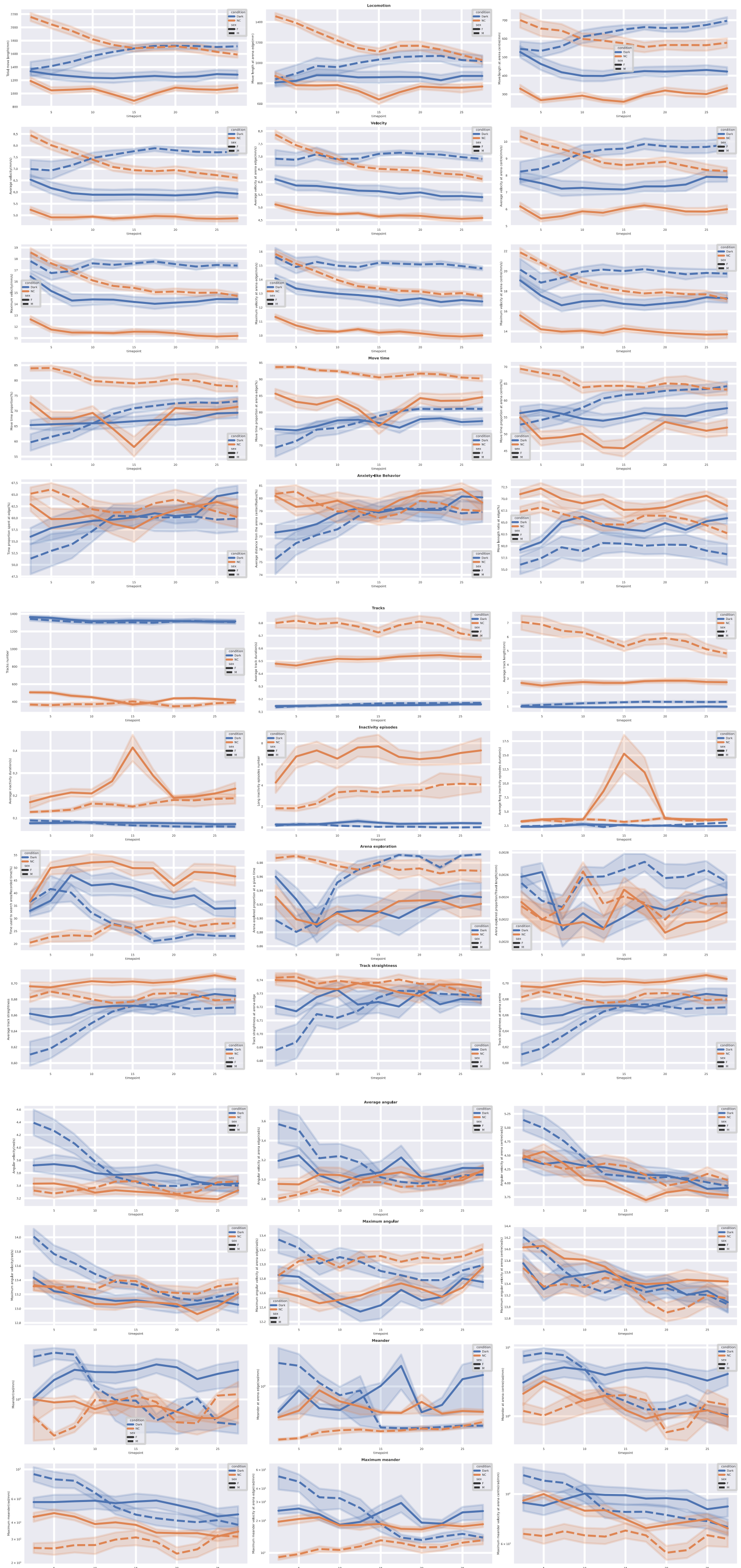

Fig 25, Grouped timeseries plot of drosophila locomotion behavior demo. The demo showed fly locomotion changing pattern across timeline between under normal lighting and under dark mode.

- interaction_analysis_sliced_plot.pdf. Grouped timeseries plot of drosophila social behavior. Fig 26 makes demonstration on this file.

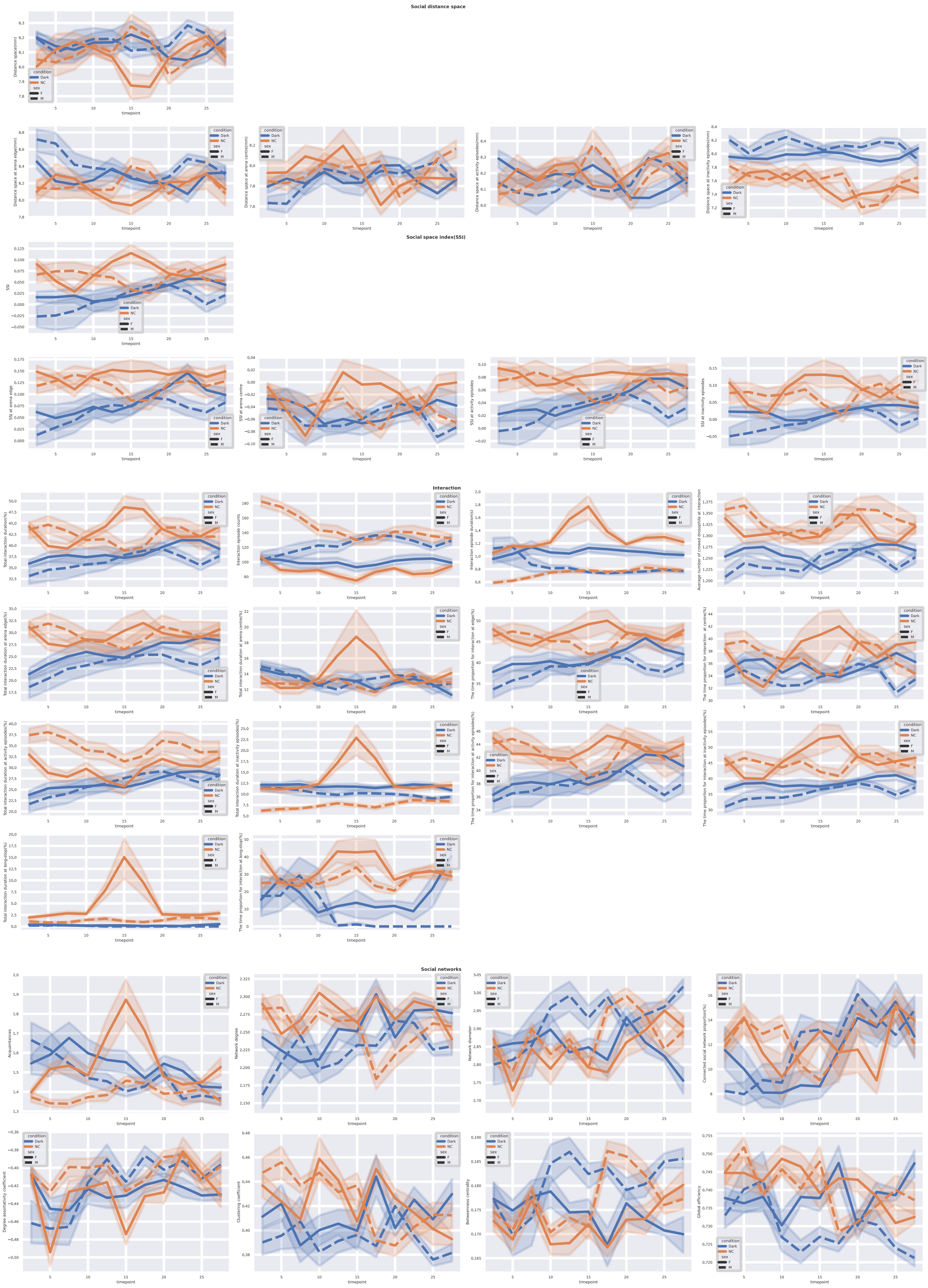

Fig 26, Grouped timeseries plot of drosophila social behavior demo. The demo showed fly social behavior changing pattern across timeline between under normal lighting and under dark mode.

- Integrated each-video-averaged fly behavior feature data

    - avg_motion.csv. Integrated each-video-averaged fly locomotion behavior data.
    - avg_interaction.csv. Integrated each-video-averaged fly social behavior data.
    - avg_motion_sliced.csv. Integrated each-video-averaged fly locomotion behavior data at sequential timepoint.
    - avg_interaction_sliced.csv. Integrated each-video-averaged fly social behavior data at sequential timepoint.
- Integrated fly behavior feature data for each fly
    - ind_motion.csv. All locomotion behavior data for each fly.
    - ind_interaction.csv. All social behavior data for each fly.
    - ind_motion_sliced.csv. Locomotion behavior data at sequential timepoint for each fly.
    - ind_interaction_sliced.csv. Social behavior data at sequential timepoint for each fly.
- Detailed analysis for each video
    - *_ind_motion.csv. Locomotion behavior data for each fly in the video.
    - *_ind_interaction.csv. Social behavior data for each fly in the video.
    - * _ind_motion_sliced.csv. Locomotion behavior data at sequential timepoint for each fly in the video.
    - * _ind_interaction_sliced.csv. Social behavior data at sequential timepoint for each fly in the video.
    - * _avg_motion.csv. Locomotion behavior data averaged by flies in the video.
    - *_avg_interaction.csv. Social behavior data averaged by flies in the video.
    - _avg_motion_sliced.csv. Locomotion behavior data at sequentially timepoint averaged by flies in the video.
    - _avg_interaction_sliced.csv. Social behavior data at sequentially timepoint averaged by flies in the video.
    - *_interaction_bytimeline.csv. Fly interaction event records.
    - *_group_interaction_subject_to_subject.csv. Total interaction duration of flies to each other.
    - *_ind_moveinfo.csv. Fly move velocity, angular, whether fly locates at the arena edge at each frame included.
    - *_ind_moveinfo2.csv. Fly social space, interaction at each frame included.
- Visualization plot for each video
    - *_motion_trace.png. Fly trace in arena. Plots for each fly are provided. Fig 27 makes demonstration on this file.

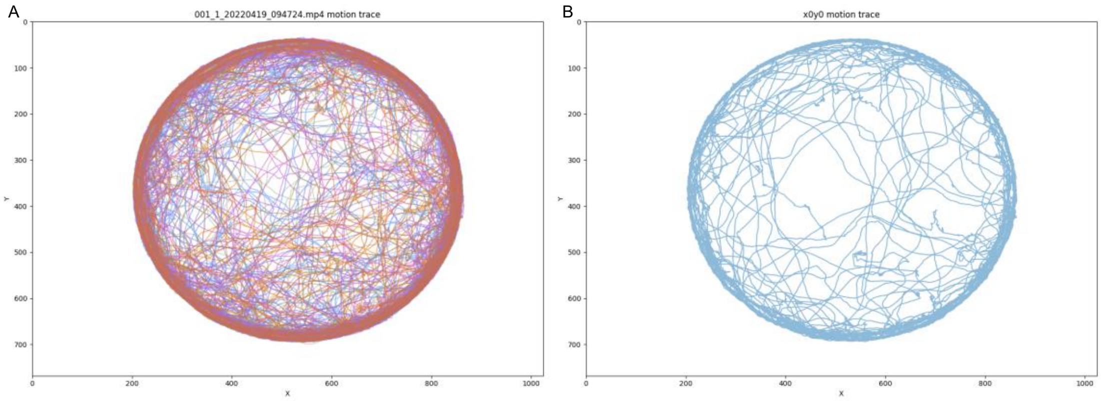

Fig 27, fly motion trace plot during the video length. A, all fly trace were plotted. B, motion trace of fly with id 0.

- * _social_space.png. Timeseries plot of social space distance. Plot for each fly and the video averaged feature are provided. Fig 28 makes demonstration on this file.

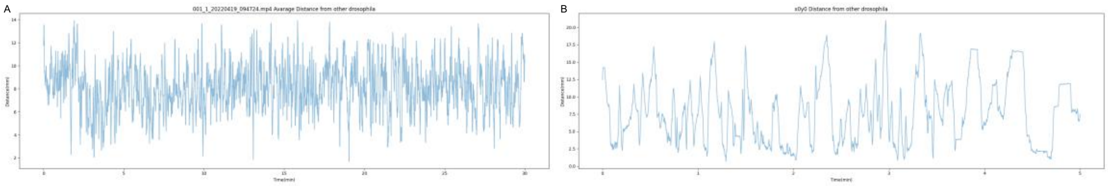

Fig 27, plot on fly social space during the video length. A, averaged fly social space distance. B, social space of fly with id 0.

- *_motion_velocity.png. Timeseries plot of velocity. Plot for each fly and the video averaged feature are provided. Fig 29 makes demonstration on this file.

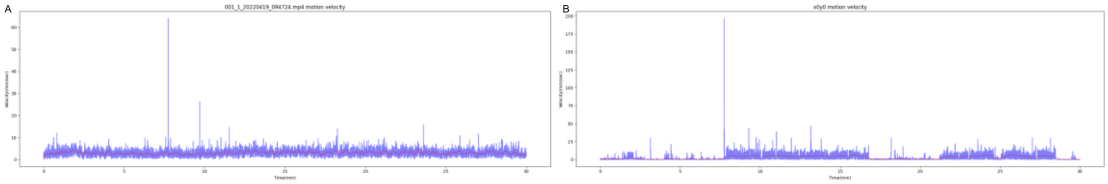

Fig 29, plot on fly velocity during the video length. A, averaged fly velocity. B, velocity of fly with id 0.

- *_group_interaction.jpg. Interaction network. Edge denotes the time duration of fly interaction. Fig 30 makes demonstration on this file.

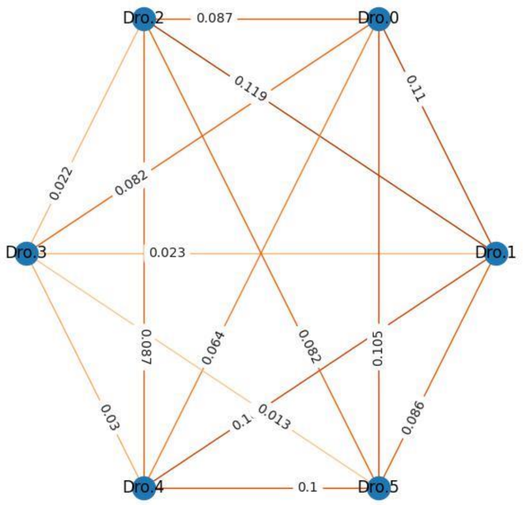

Fig 30, fly social interaction network.

DVT desktop edition stores its results at the **Saveplace**, including:

- Background image
- Video with background removal
- Fly trace files
  - *.ffindex
  - *-colors.txt
  - *-info.txt
  - *-position.csv, fly position at every frame of the video.
- Detailed analysis for each video
  - *_ind_motion.csv. Locomotion behavior data for each fly in the video.
  - *_ind_interaction.csv. Social behavior data for each fly in the video.
  - * _avg_motion.csv. Locomotion behavior data averaged by flies in the video.
  - *_avg_interaction.csv. Social behavior data averaged by flies in the video.
  - *_interaction_bytimeline.csv. Fly interaction event records.
  - *_group_interaction_subject_to_subject.csv. Total interaction duration of flies to each other.
  - *_ind_moveinfo.csv. Fly move velocity, angular, whether fly locates at the arena edge at each frame included.
  - *_ind_moveinfo2.csv. Fly social space, interaction at each frame included.
- Visualization plot for each video
  - *_motion_trace.png. Fly trace in arena. Plot for each fly and the video averaged feature are provided.
  - * _social_space.png. Timeseries plot of social space distance. Plot for each fly and the video averaged feature are provided.
  - *_motion_velocity.png. Timeseries plot of velocity. Plot for each fly and the video averaged feature are provided.
  - *_group_interaction.jpg. Interaction network. Edge denotes the time duration of fly interaction.

4.4.3. Analysis output file definition

DVT outputs serval data or visualization files. In this section a detailed explanation is made on the column definition of serval data file for convenience of second development or further investigation.

- *-position.csv.

*-position.csv refers to the generated fly trace data file with columns definition shown in table 1.

Table 1 fly trace data column definition

Index	Column name	Definition
1	position	Frame in the video
2	x0	x-coordinate of fly with id 0
3	y0	y-coordinate of fly with id 0
4	x1	x-coordinate of fly with id 1
5	y1	y-coordinate of fly with id 1
	…	…

- *_motion.csv

*_motion.csv refers to fly locomotion behavior data file with columns shown in table 2. DVT generates four types motion data file, i.e. *_ind_motion.csv, *_avg_motion.csv, *_ind_motion_sliced.csv and *_avg_motion_sliced.csv. Note some columns might not be available at all four type files.

Table 2 fly locomotion behavior data column definition

Index	Column name	Definition
1	videoname	Video name
2	id	Fly id, for example, 0, 1, 2…
3	xlabel	x-coordinate label of fly, for example, x0, x1, x2…
4	ylabel	y-coordinate label of fly, for example, y0, y1, y2…
5	timepoint	Middle point of time window
6	search_area_time	Exploration efficiency by time
7	search_area	Area explored by given time
8	search_area_unit_move	Exploration efficiency by travel length
9	velocity_threshed	Avg. velocity
10	velocity_at_edge	Avg. velocity at arena edge
11	velocity_at_center	Avg. velocity at centre
12	max_velocity_threshed	Max. velocity
13	max_velocity_at_edge	Max. velocity at arena edge
14	max_velocity_at_center	Max. velocity at centre
15	total_move_length_threshed	Total move length
16	movelength_at_edge	Move length at arena edge
17	movelength_at_center	Move length at arena centre
18	move_time_threshed	Total move time
19	move_proportion_at_edge	Move time prop. at arena edge
20	move_proportion_at_center	Move time prop. at arena centre
21	r_dist	Avg. distance from the arena center
22	r_edge	Time prop. spent at edge

23	tracks_num	Tracks number
24	tracks_duration	Avg. track duration
25	tracks_length	Avg. track length
26	long_stop_num	Long stop episodes number
27	stop_duration	Avg. inactivity duration
28	long_stop_duration	Avg. long stop episodes duration
29	track_straightness	Avg. track straightness
30	track_straightness_non_edge	Track straightness at arena centre
31	track_straightness_at_edge	Track straightness at arena edge
32	angular_velocity	Avg. angular velocity
33	angular_velocity_non_edge	Avg. angular velocity at arena centre
34	angular_velocity_at_edge	Avg. angular velocity at arena edge
35	max_angular_velocity	Max. angular velocity
36	max_angular_velocity_non_edge	Max. angular velocity at arena centre
37	max_angular_velocity_at_edge	Max. angular velocity at arena edge
38	meander	Avg. meander
39	meander_non_edge	Avg. meander at centre
40	meander_at_edge	Avg. meander at arena edge
41	max_meander	Max. meander
42	max_meander_non_edge	Max. meander at centre
43	max_meander_at_edge	Max. meander at arena edge
44	num	Number of flies in the arena
45	sex	Fly gender
46	replicate	Experiment replicate
47	condition	Experiment condition
48	genotype	Fly genotype

- *_interaction.csv

*_ interaction.csv refers to fly social behavior data file with columns shown in table 3. DVT generates four types social behavior data file, i.e. *_ind_interaction.csv, *_avg_interaction.csv, *_ind_interaction_sliced.csv and *_avg_interaction_sliced.csv. Note some columns might not be available at all four type files.

Table 3 fly social behavior data column definition

Index	Column name	Definition
1	videoname	Video name
2	id	Fly id, for example, 0, 1, 2…
3	xlabel	x-coordinate label of fly, for example, x0, x1, x2…
4	ylabel	y-coordinate label of fly, for example, y0, y1, y2…
5	timepoint	Middle point of time window
6	acquaintances	Acquaintance
7	distance_space	Social space distance
8	distance_space_at_edge	Space distance at arena edge

9	distance_space_at_center	Space distance at arena centre
10	distance_space_at_move	Space distance at activity episodes
11	distance_space_at_stop	Space distance at inactivity episodes
12	SSI	SSI
13	SSI_at_edge	SSI at arena edge
14	SSI_at_center	SSI at arena centre
15	SSI_at_move	SSI at activity episodes
16	SSI_at_stop	SSI at inactivity episodes
17	interaction	Total interaction duration
18	interaction_at_edge	Interaction duration at edge
19	interaction_at_edge_proportion	Interaction time prop. at edge
20	interaction_at_center	Interaction duration at centre
21	interaction_at_center_proportion	Interaction time prop. at centre
22	interaction_at_move	Interaction duration at activity episodes
23	interaction_at_move_proportion	Interaction time prop. at activity episodes
24	interaction_at_stop	Interaction duration at inactivity episodes
25	interaction_at_stop_proportion	Interaction time prop. at inactivity episodes
26	interaction_at_long_stop	Total interaction duration at long-stop
27	interaction_at_long_stop_proportion	Interaction time prop. at long-stop
28	interaction_counts	Interaction episode count
29	interaction_duration	Interaction episode duration
30	interaction_members	Avg. number of crowded dro.
31	degree_assortativity_coefficient	Degree assortativity coefficient
32	clustering_coefficient	Clustering coefficient
33	betweenness_centrality	Betweenness centrality
34	diameter	Network diameter
35	degree	Network degree
36	Unconnected_social_network_proportion	Unconnected social network prop.
37	global_efficiency	Global efficiency
38	num	Number of flies in the arena
39	sex	Fly gender
40	replicate	Experiment replicate
41	condition	Experiment condition
42	genotype	Fly genotype

- *_group_interaction_subject_to_subject.csv

*_group_interaction_subject_to_subject.csv records total interaction duration of flies to each other. The column definition is shown in table 4.

Table 4 *_group_interaction_subject_to_subject.csv column definition

index	Column name	Definition
1	id1	The first fly id

2	id2	The second fly id
3	interaction	the interaction prop. between the first fly and the second fly in the first fly's all interaction
4	videoname	Video name
5	xlabel1	x-coordinate label of the first fly, for example, x0, x1, x2…
6	xlabel2	x-coordinate label of the second fly, for example, x0, x1, x2…
7	ylabel1	y-coordinate label of the first fly, for example, y0, y1, y2…
8	ylabel2	y-coordinate label of the first fly, for example, y0, y1, y2…

- *_interaction_bytimeline.csv

*_interaction_bytimeline.csv records fly interaction event records. The column definition is shown in table 5.

Table 5 *_interaction_bytimeline.csv column definition

index	column name	definition
1	Begin	The beginning frame of an interaction event
2	End	The ending frame of an interaction event
3	time_duration	Interaction duration time (frames)
4	interactions	Intermediate Data, default is 1
5	subject1	The first fly id in the interaction event
6	subject2	The second fly id in the interaction event

4.4.4. Use fishbone diagram in result interpretation

DVT paradigm outputs multi behavior features and there's a large number of potential relationships between features. It is highly recommended using fishbone diagram to conduct the feature deviation causes decomposition in the interpretation of results. For convenience, serval fishbone diagram template is provided in this manual (fig 31).

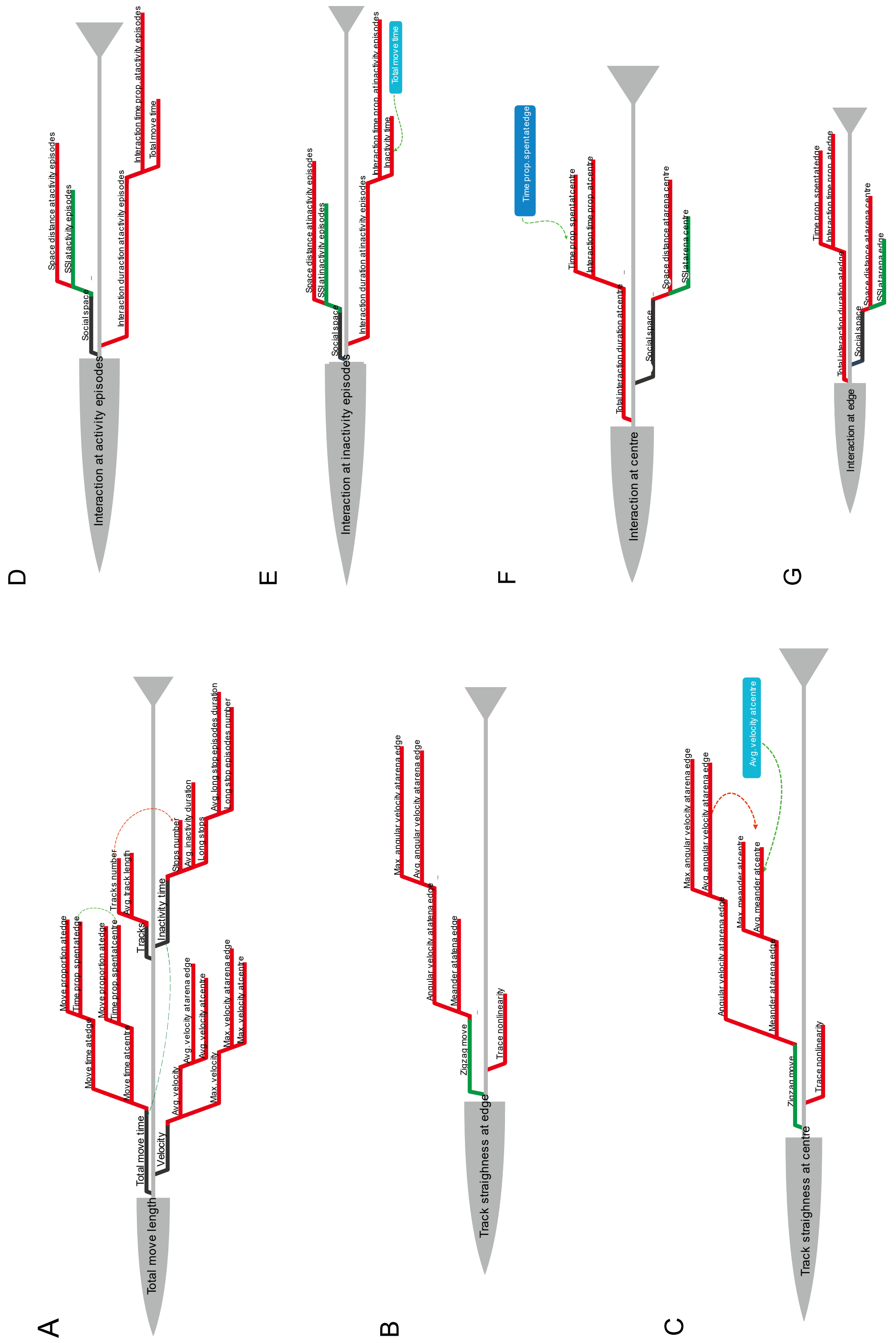

Fig 31, fishbone diagram template

4.4.5. Track straightness related features and Network topology features interpretation

Compared with velocity or move length, track straightness and network topology related features are not easy to interpretation.

So, for track straightness, DVT provided a visualized chart to explain the relationship between

track straightness, angular velocity and meander. Sometimes fly walks in zigzag mode, that is keeping turning left and right while moving (Fig 32, A). Since the time window to calculate the angular velocity was set to 0.2s, the average move length in the time window is about 1mm to 3mm, that is approximately 0.5 or 1 fly body length. Hence angular velocity is a good feature to describe the zigzag movement in DVT (Fig 32, B). While the meander is calculated as the ratio between turning angular and the moving distance.

In another hand, the track straightness was calculated in DVT with a time window set to 1s. In this larger window, flies can move a longer distance. As shown in fig32, C-F, track straightness depends on the trace linearity and zigzag move. If fly has a specific target to reach and it move straightly (fig 32, C,D), the track straightness is determined by fly's zigzag move. But if the trace is non-linear itself, track straightness would be lower (E, F).

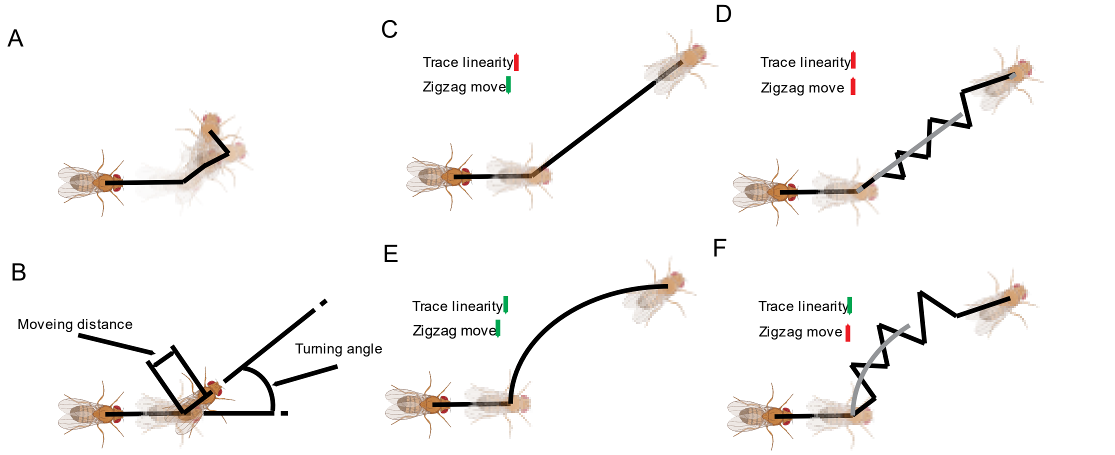

Fig 32, move straightness interpretation. A, fly moves in zigzag mode. B, angular velocity and meander calculation. C-F, different move straightness corresponds to different move pattern.

The interpretation of social network topology related features is also a tricky work. Plenty topology knowledge is needed for a correct interpretation. To mitigate this gap, we generated three typical social networks, string-like network, star-like network, highly connected social network (Fig 33, A-C) and their variants. The topology features of all network variants were calculated and ordered ascendingly (Fig 33, D).

In the interpretation work, let's take the fly behavior change under dark mode compared with normal lightning as an example. In the dark mode, fly social network showed a decreased network degree, decreased cluster coefficient and decreased degree assortativity coefficient. Check the three corresponding columns in fig 33, D and easily, it could be found that, all three features of star-like network are relatively lower than others. Hence fly social interaction depends on the hub-fly in the dark mode can be deduced.

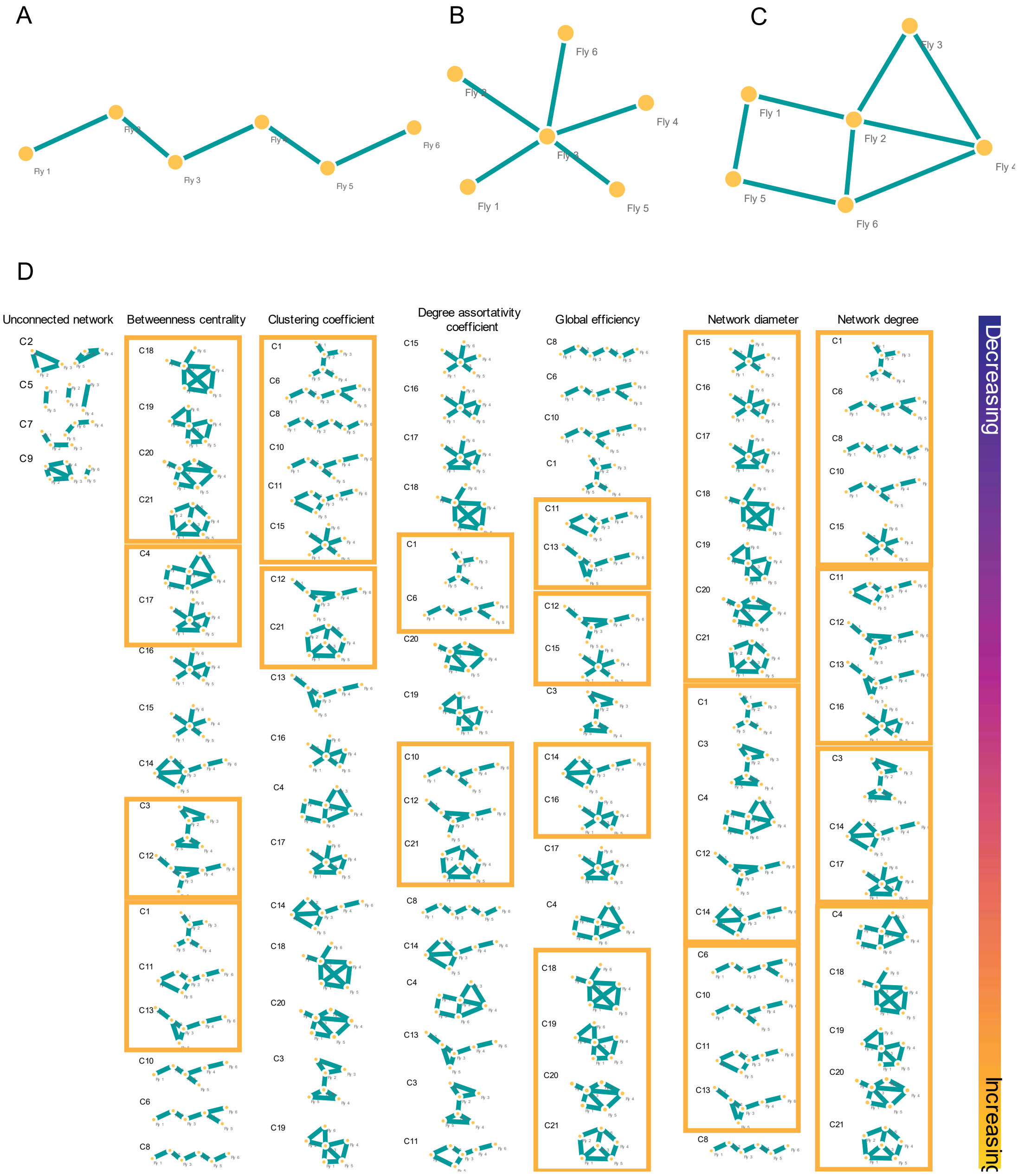

Fig 33, network topology interpretation. A, string-like network. B, star-like network. C, highly connected social network. D, topology related features of demonstrative networks. Networks with same feature value was put into the orange box.

4.5. Behavior criteria practices and DVT analysis parameter configuration

4.5.1. Considerations on behavior criteria setup

Behavior criteria varied across literatures. Following we listed the reported practices in fly behavior criteria setup and the criteria used in DVT paradigm.

- Interaction criteria

Sechneider et al. proposed an automated identification method of social interaction criteria in Drosophila by subtracting the simulated trails from the real ones. Three criteria, including encounter distance (about 1.25-3 body length), encounter angle (90-160°), and encounter time (0.4-1.1s) was recommended for different species of flies[8]. Ctrax defined the interaction or encounter event as those trajectory intervals in which the distance between a pair of flies was less

than 10 mm in which was ~ 3 body length [1]. Flytrack defined touch as a head-to tail touch event in a single frame. A touch interaction is obtained when a touch lasts for at least 15 consecutive frames (~0.5s)[9].

However, we noticed that Hoyer et al. reported a whole Male Aggression, a pair of five-dayold CS males performed 3.85 +-2.82 lunges/min and a complete lunges process lasts for 118ms[10]. Hence, if the we take interaction time larger than 0.4s as our criteria, lots of interaction could be treated as false negatives. As reported by Simon et al, erroneous orientation was about 1 s-1 at floor to 4 s-1 for individual flies [11]. This is kind of unacceptable. Thus, undirected social network was constructed to avoid orientation error in DVT paradigm. Therefore, the flies had an interaction from each other if the distance between them was less than 1.5 * body length in DVT.

- Move criteria

Colomb et al. reported flies with velocity less than (1mm/s) was classified as at rest [12]. White et al. reported the threshold as 0.25mm/s[13]. In DVT, the criteria was set to 0.5mm/s. The speed was below the lower threshold (0.5 mm/s) the fly was classified as at rest or inactive.

- Edge boundary

Bath et al. set the inner 36mm in a 54mm circular chamber as the edge boundary [14], while Besson et al. made it to 20mm in a 40mm*40mm squared chamber[15]. We investigated the spatial preference of fly in DVT chamber. Then a peak emerged at 0.6mm-3mm from the arena edge for fly spatial distribution. Hence, in DVT paradigm we set 3mm from the arena edge as the boundary between edge and centre.

- Angular and meander time windows

The time window of angular velocity and meander related features was set to 0.2s which is consistent with Martin's work[4]. 0.2s is equivalent to the average step period at average speed 7.2mm/s identified by Mendes's work[16].

- Track straightness

The time window of track straightness related features was set to 1s which is consistent with Aggarwal's work [3].

- Network size

The Network size is set to 0.5 in DVT. That is the moving-social-network-window represents 50% of the total number of interactions possible for flies. For example, the moving-social-network-window is 8 for a 6-fly social community in the chamber. DVT calculates the network features for the social network composed by the first 8 interactions and then the second network by the 9th to 16th interactions. For 8-fly community, the moving-social-network-window is 14. This calculation procedures are inherited from Schneider's work [7].

4.5.2. DVT analysis parameter configuration

DVT has serval configurable parameters to make a flexible analysis paradigm. For DVT server edition, the config file is stored at `Dorsophila_track_DVT/config.ini, researcher can use vi or other editors to change the parameter set.`

There're three sub-sections in DVT server edition. This parameters in *Fixed_para* section connects to the hardware. An annotation of the para is shown below.

```
# video fps
fps = 30
#frames to generate background. the background is kind of median of t
hese frames
```

```
frames_to_generate_background = 200
# images extracted for fly body size measurement. this can be done by imagej
imgs_for_body_size_measure = 3
# recorded video resolution x-coordinate
x = 1024
# recorded video resolution y-coordinate
y = 768
# resized video resolution y-coordinate. The resized video is to be pipped into UMATracker
resized_x = 640
# resized video resolution y-coordinate. The resized video is to be pipped into UMATracker
resized_y = 480
# scaling ratio. 1.6=1024/640
scaling = 1.6
# arena diameter(mm)
diameter = 37
# time_periods for sliced analysis. This were set for 30-mins video
time_periods=11
time_windows=2.5
```

Here we would like to make a more detailed explanation on the last two parameters. DVT server edition can conduct analysis on the sliced timeline which gives an intuitive vision on drosophila behavior pattern across the moving time window. In this analysis, the move step is 2.5 minutes which is set by **time_windows**, and the window size is 5 minutes which is two times of the **time_windows**. In this way, for 30minutes video, there're 11 sliced periods shown in table 6. If researcher wants to change these two parameters, make sure the sliced time periods should cover the whole timeline.

Table 6, sliced time window definition in DVT

index	timepoint			
1	2.5	0	2.5	5
2	5	2.5	5	7.5
3	7.5	5	7.5	10
4	10	7.5	10	12.5
5	12.5	10	12.5	15
6	15	12.5	15	17.5
7	17.5	15	17.5	20
8	20	17.5	20	22.5
9	22.5	20	22.5	25
10	25	22.5	25	27.5
11	27.5	25	27.5	30

The second section is Adjustable_para. Most parameters in this part are thresholds used in behavior calculation. The definition is shown below. Usage of the corresponding parameters can be found at section 4.4.1.

```
# drosophila body length (in pixel)
sensing_area = 60
# 0.5 mm/s. Fly is inactive below this threshold.
move_thresh = 0.5
# 3mm. Arena edge threshold.
r_thresh = 3
# 95%. The 95th percentile of features is taken as the maximum value
max_v_thresh = 0.95
#0.2s. The angular velocity and meander calculation window length
angular_velocity_window = 0.2
#1s. The track straightness calculation window length
track_straightness_window = 1
# 2s. Inactivity with duration longer than 2s is defined as long stop episodes.
long_stop_thresh = 2
# 10 minutes. The explorated area is calculated in the first 10 minutes of recorded video.
area_search_time_thresh = 10
# 80%. The time used or move length travelled to complete 80% of the arena exploration
area_thresh = 0.8
# interaction criteria. The distance of two drosophila < 1.5*bodylength is an interaction event.
scale_to_interaction = 1.5
#5mm. SSI bins for Social space index
ssi_bin = 5
#50%. The moving-social-network-window represents 50% of the total number of interactions possible for flies.
network_size = 0.5
```

The last section is Plot_para. The definition is shown below. The **plot_factors** records the sequence in the plot. The last variable factor will be taken as hue parameter in the seaborn plot. And the second last variable factor will be taken as the x-axis labels. If in these five factors, only one variable factor exists, it will be set as the x-axis labels. The **plot_by_video** controls whether to use video-averaged features. If trace correction was done and researchers are interested in individual fly's behavior, this parameter should be 0, otherwise 1.

```
#Sequenced by x-axis/hue
plot_factors= replicate,sex,num,condition,genotype
# 1 for boxplot; 2 for violin plot
plot_type = 1
#1 for plot swarm points; 0 for plot no points
plot_swarm = 1
#1 for by video averaged data; 0 for by individual fly data
```

```
plot_by_video=1
#1 for plot with logged y scale; 0 for plot without logged y scale.
#This works for meander related measures only
plot_with_logged_yscale=1
#Confidence interval 0-100 for timeline plot
ci=50
```

Reference

1. Branson, K., et al., *High-throughput ethomics in large groups of Drosophila.* Nat Methods, 2009. **6**(6): p. 451-7.
2. Berg, S., et al., *ilastik: interactive machine learning for (bio)image analysis.* Nat Methods, 2019. **16**(12): p. 1226-1232.
3. Aggarwal, A., H. Reichert, and K. VijayRaghavan, *A locomotor assay reveals deficits in heterozygous Parkinson's disease model and proprioceptive mutants in adult Drosophila.* Proc Natl Acad Sci U S A, 2019. **116**(49): p. 24830-24839.
4. Martin, J.R., *A portrait of locomotor behaviour in Drosophila determined by a video-tracking paradigm.* Behav Processes, 2004. **67**(2): p. 207-19.
5. McNeil, A.R., et al., *Conditions Affecting Social Space in Drosophila melanogaster.* J Vis Exp, 2015(105): p. e53242.
6. Simon, A.F., et al., *A simple assay to study social behavior in Drosophila: measurement of social space within a group.* Genes Brain Behav, 2012. **11**(2): p. 243-52.
7. Schneider, J., M.H. Dickinson, and J.D. Levine, *Social structures depend on innate determinants and chemosensory processing in Drosophila.* Proc Natl Acad Sci U S A, 2012. **109 Suppl 2**: p. 17174-9.
8. Schneider, J. and J.D. Levine, *Automated identification of social interaction criteria in Drosophila melanogaster.* Biol Lett, 2014. **10**(10): p. 20140749.
9. Liu, G., et al., *A simple computer vision pipeline reveals the effects of isolation on social interaction dynamics in Drosophila.* PLoS Comput Biol, 2018. **14**(8): p. e1006410.
10. Hoyer, S.C., et al., *Octopamine in male aggression of Drosophila.* Curr Biol, 2008. **18**(3): p. 159-67.
11. Simon, J.C. and M.H. Dickinson, *A new chamber for studying the behavior of Drosophila.* PLoS One, 2010. **5**(1): p. e8793.
12. Colomb, J., et al., *Open source tracking and analysis of adult Drosophila locomotion in Buridan's paradigm with and without visual targets.* PLoS One, 2012. **7**(8): p. e42247.
13. White, K.E., D.M. Humphrey, and F. Hirth, *The dopaminergic system in the aging brain of Drosophila.* Front Neurosci, 2010. **4**: p. 205.
14. Bath, E., J. Thomson, and J.C. Perry, *Anxiety-like behaviour is regulated independently from sex, mating status and the sex peptide receptor in Drosophila melanogaster.* Animal Behaviour, 2020. **166**: p. 1-7.
15. Besson, M. and J.R. Martin, *Centrophobism/thigmotaxis, a new role for the mushroom bodies in Drosophila.* J Neurobiol, 2005. **62**(3): p. 386-96.
16. Mendes, C.S., et al., *Quantification of gait parameters in freely walking wild type and sensory deprived Drosophila melanogaster.* Elife, 2013. **2**: p. e00231.
