# Supplementary material for: DVT: a high-throughput analysis pipeline for locomotion and social behavior in adult Drosophila melanogaster
Source: Cell Biosci. 2023 Oct 5;13:187. doi: 10.1186/s13578-023-01125-0 (PMC10557313; doi:10.1186/s13578-023-01125-0)
Supplement: Supplementary file 8 — Additional file 8: Supplemental Figures Figure S1 DVT analysis pipeline. A, Traditional analysis pipeline with reID error corrected. B, DVT analysis pipeline by average-by-video value of behavior metrics. C, Average-by-video fly behavior metrics has a strong correlation before and after reID error correction. D, Analysis pipeline for heterogeneous Dro. chamber. Statistical analysis: Pearson correlation were calculated between the average-by-video fly behavior metrics before and after reID error correction of n = 8 videos (4 videos from w1118 flies, and 4 videos from OR flies). Figure S2 Behavior deviation for flies in 2 mm-height chamber. A, Effect size for locomotion behavior deviation of drosophila in 2 mm-height chamber from that in 3 mm-height chamber. A.1 ~ A.6, Scatter plots for track straightness at arena edge of male or female flies in 2 mm-height or 3 mm-height chamber from different experimental replicates. B, Effect size for social behavior deviation of drosophila in 2 mm-height chamber from that in 3 mm-height chamber. Interpretation of the radar plot: The effect size was calculated to illustrate the behavior feature deviation of flies in treatment group chambers from that in control chambers. In the calculation, the mean difference was divided by maximum value of standard deviations of the control group and treatment group to denote the effect size. In this case, the control group was fly behavior features in 3 mm-height chamber and the treatment group was that in 2 mm-height profile. In general, both the mean difference between two groups and the intragroup variance affected the magnitude of effect size. if the magnitude of effect size is smaller than 0.2, it is considered to be a small deviation, an effect size of around 0.5 is considered to be a medium deviation, and an effect size of 0.8 or larger is considered to be a large deviation. In the visualization, fly behavior in 3 mm-height chamber was set as the bench and represented by the bold black ci [file 13578_2023_1125_MOESM8_ESM.pdf]

Figure S1

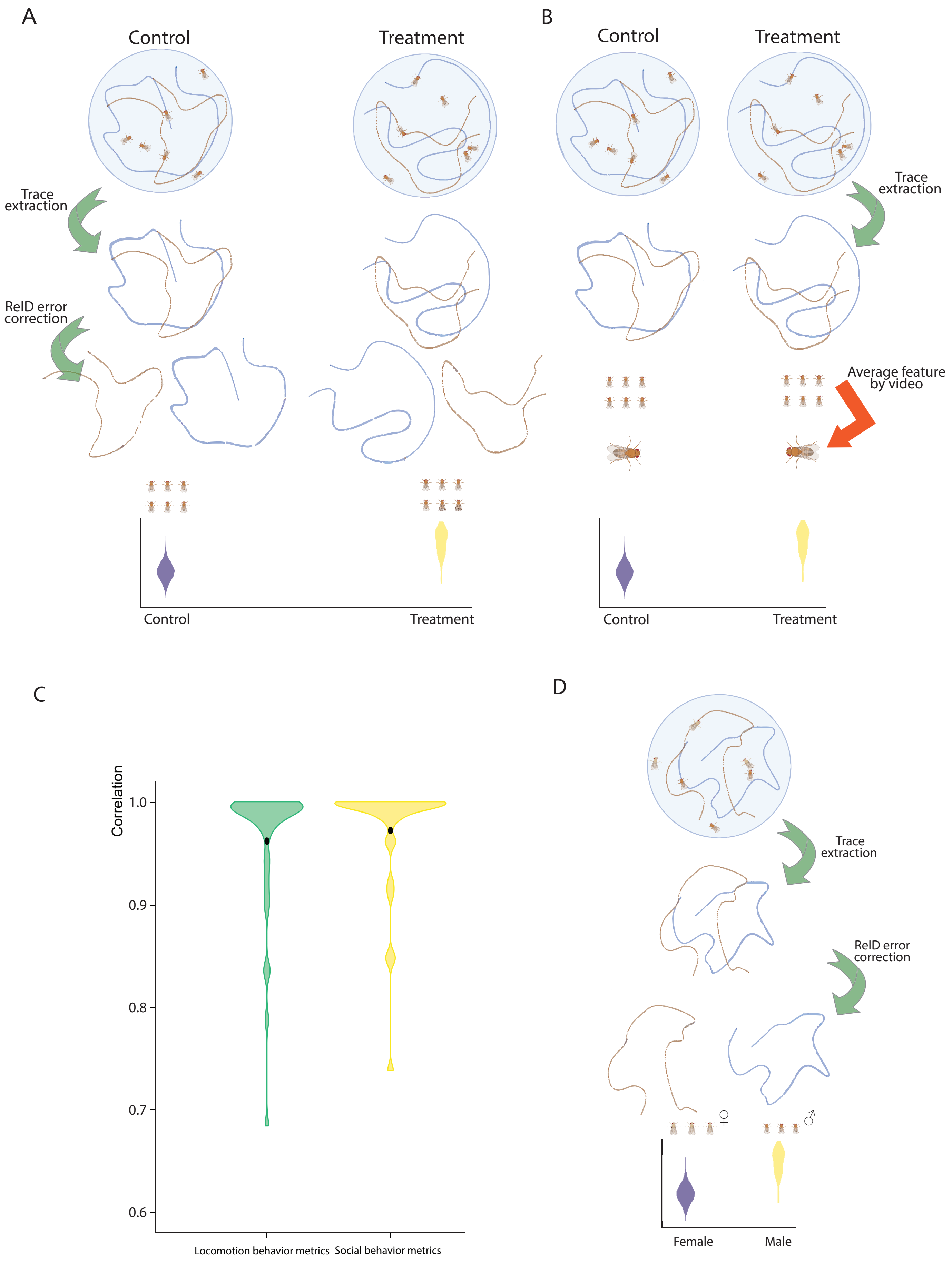

A
Control
Treatment
Trace extraction
ReID error correction
Control
Treatment
B
Control
Treatment
Trace extraction
Average feature by video
Control
Treatment
C
Correlation
1.0
0.9
0.8
0.7
0.6
Locomotion behavior metrics
Social behavior metrics
D
Trace extraction
ReID error correction
♀
♂
Female
Male

Figure S2

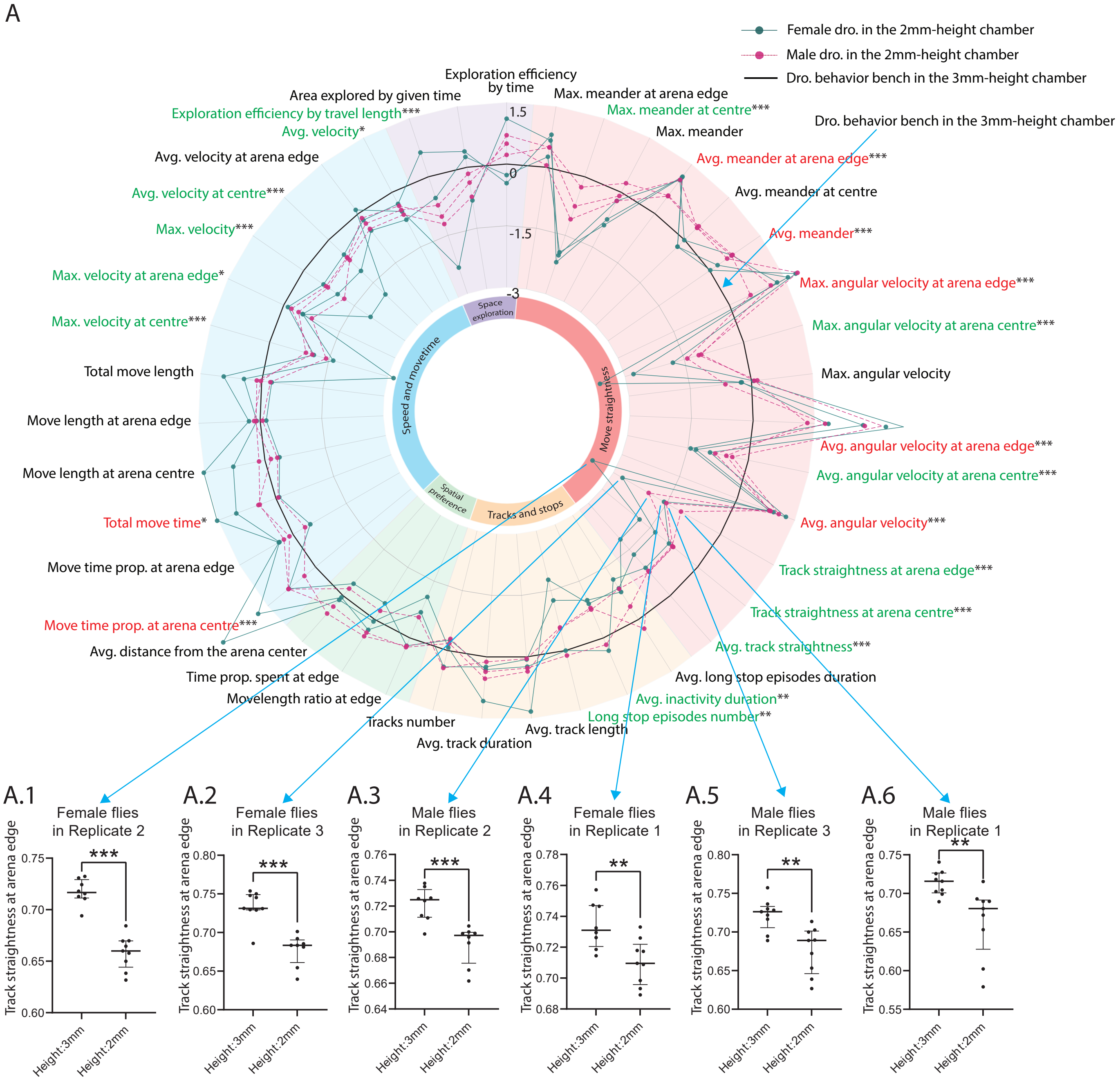
A
Female dro. in the 2mm-height chamber
Male dro. in the 2mm-height chamber
Dro. behavior bench in the 3mm-height chamber
Dro. behavior bench in the 3mm-height chamber
Exploration efficiency by time
Area explored by given time
Exploration efficiency by travel length***
Avg. velocity*
Avg. velocity at arena edge
Avg. velocity at centre***
Max. velocity***
Max. velocity at arena edge*
Max. velocity at centre***
Total move length
Move length at arena edge
Move length at arena centre
Total move time*
Move time prop. at arena edge
Move time prop. at arena centre***
Avg. distance from the arena center
Time prop. spent at edge
Movelength ratio at edge
Tracks number
Avg. track duration
Avg. track length
Long stop episodes number**
Avg. inactivity duration**
Avg. long stop episodes duration
Avg. track straightness***
Track straightness at arena centre***
Track straightness at arena edge***
Avg. angular velocity***
Avg. angular velocity at arena centre***
Avg. angular velocity at arena edge***
Max. angular velocity
Max. angular velocity at arena centre***
Max. angular velocity at arena edge***
Avg. meander***
Avg. meander at centre
Avg. meander at arena edge***
Max. meander
Max. meander at centre***
Max. meander at arena edge
1.5
0
-1.5
-3
Space exploration
Speed and movetime
Spatial preference
Tracks and stops
Move straightness
A.1
Female flies in Replicate 2
A.2
Female flies in Replicate 3
A.3
Male flies in Replicate 2
A.4
Female flies in Replicate 1
A.5
Male flies in Replicate 3
A.6
Male flies in Replicate 1
Track straightness at arena edge
***
**
Height:3mm
Height:2mm

B

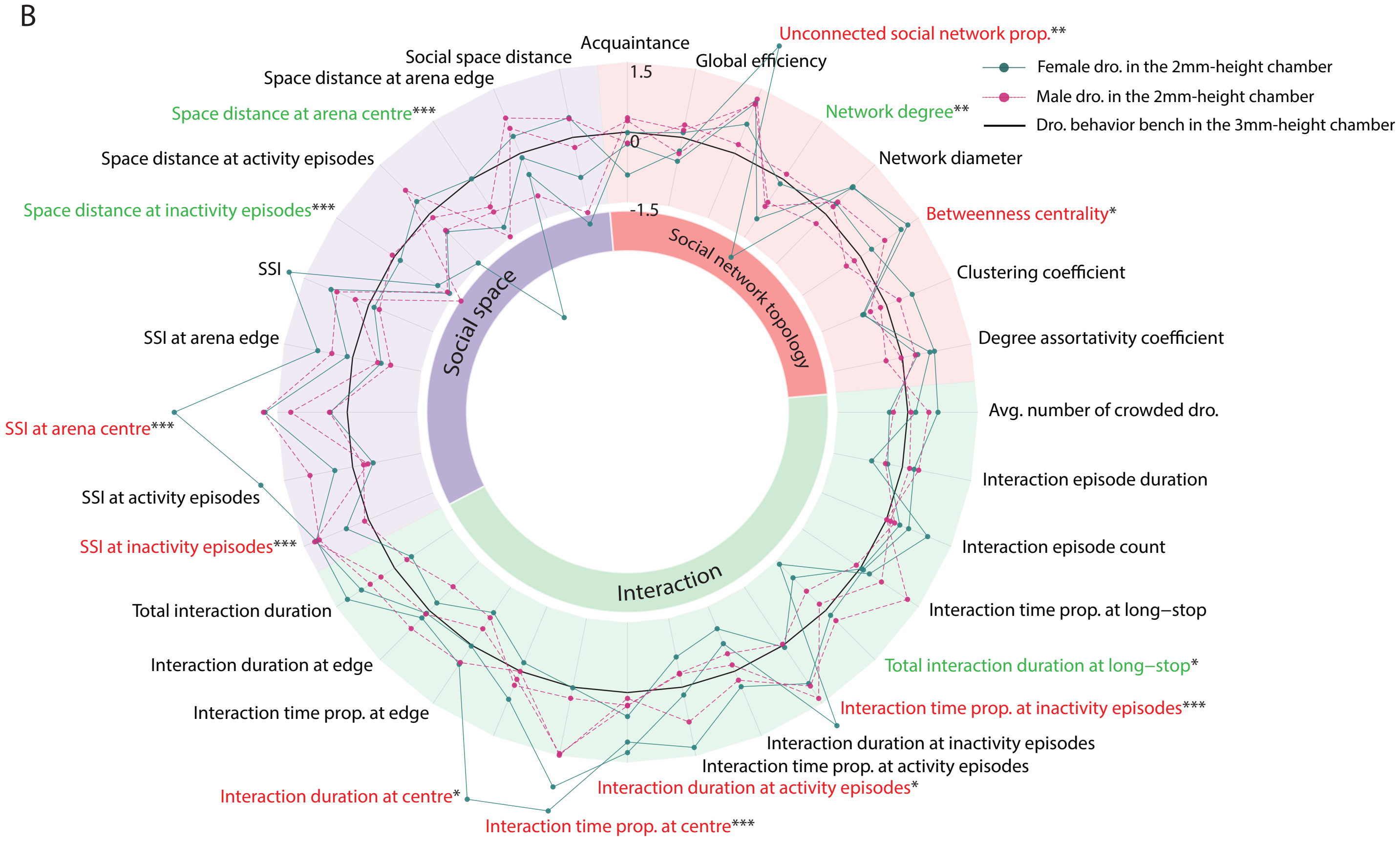
Female dro. in the 2mm-height chamber
Male dro. in the 2mm-height chamber
Dro. behavior bench in the 3mm-height chamber
1.5
0
-1.5
Social space
Social network topology
Interaction
Acquaintance
Global efficiency
Unconnected social network prop.**
Network degree**
Network diameter
Betweenness centrality*
Clustering coefficient
Degree assortativity coefficient
Avg. number of crowded dro.
Interaction episode duration
Interaction episode count
Interaction time prop. at long−stop
Total interaction duration at long−stop*
Interaction time prop. at inactivity episodes***
Interaction duration at inactivity episodes
Interaction time prop. at activity episodes
Interaction duration at activity episodes*
Interaction time prop. at centre***
Interaction duration at centre*
Interaction time prop. at edge
Interaction duration at edge
Total interaction duration
SSI at inactivity episodes***
SSI at activity episodes
SSI at arena centre***
SSI at arena edge
SSI
Space distance at inactivity episodes***
Space distance at activity episodes
Space distance at arena centre***
Space distance at arena edge
Social space distance

Figure S3

A

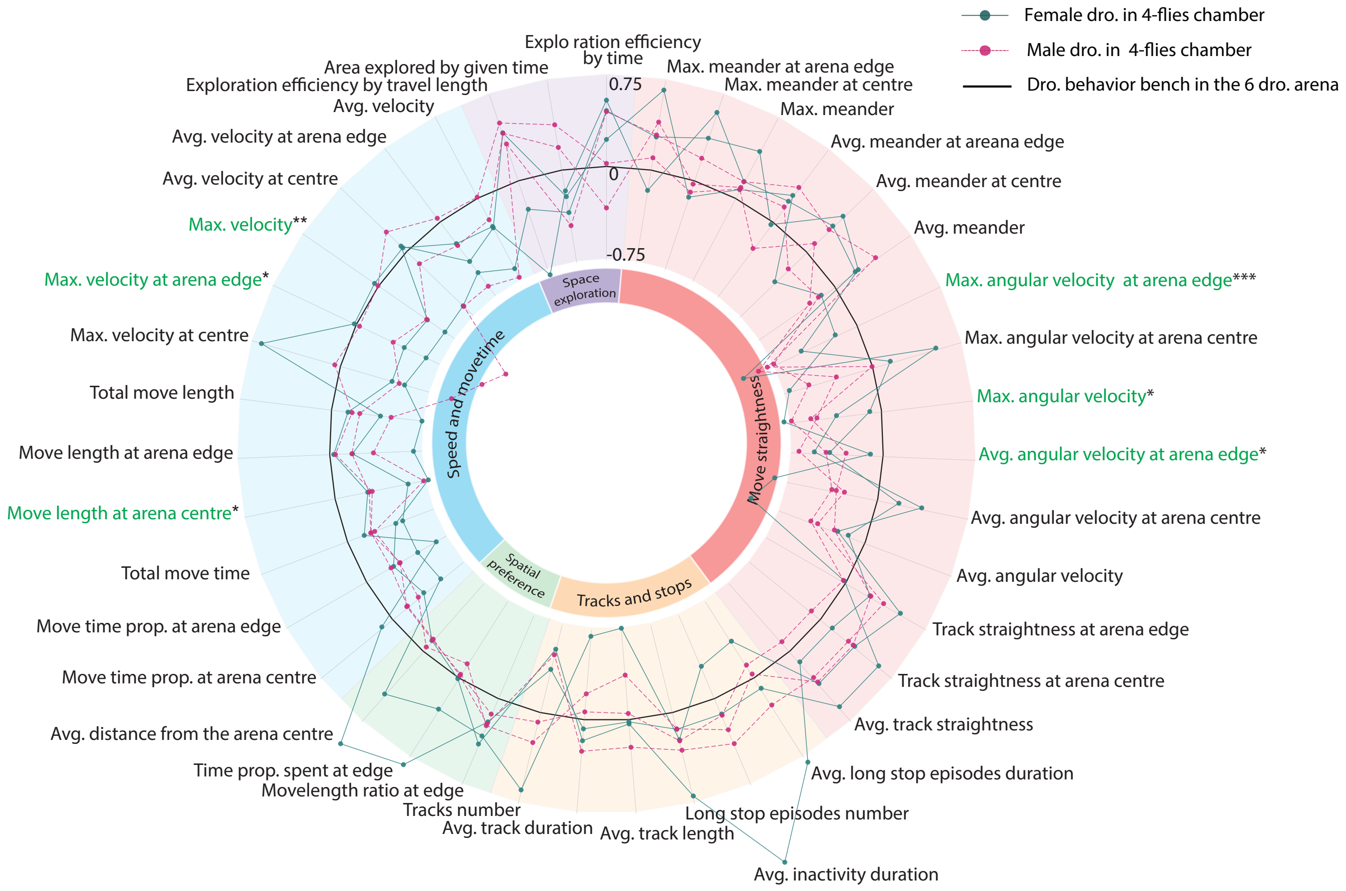
Female dro. in 4-flies chamber
Male dro. in 4-flies chamber
Dro. behavior bench in the 6 dro. arena
Explo ration efficiency by time
0.75
0
-0.75
Area explored by given time
Exploration efficiency by travel length
Avg. velocity
Avg. velocity at arena edge
Avg. velocity at centre
Max. velocity**
Max. velocity at arena edge*
Max. velocity at centre
Total move length
Move length at arena edge
Move length at arena centre*
Total move time
Move time prop. at arena edge
Move time prop. at arena centre
Avg. distance from the arena centre
Time prop. spent at edge
Movelength ratio at edge
Tracks number
Avg. track duration
Avg. track length
Long stop episodes number
Avg. inactivity duration
Avg. long stop episodes duration
Avg. track straightness
Track straightness at arena centre
Track straightness at arena edge
Avg. angular velocity
Avg. angular velocity at arena centre
Avg. angular velocity at arena edge*
Max. angular velocity*
Max. angular velocity at arena centre
Max. angular velocity at arena edge***
Avg. meander
Avg. meander at centre
Avg. meander at areana edge
Max. meander
Max. meander at centre
Max. meander at arena edge
Space exploration
Speed and movetime
Spatial preference
Tracks and stops
Move straightness

B

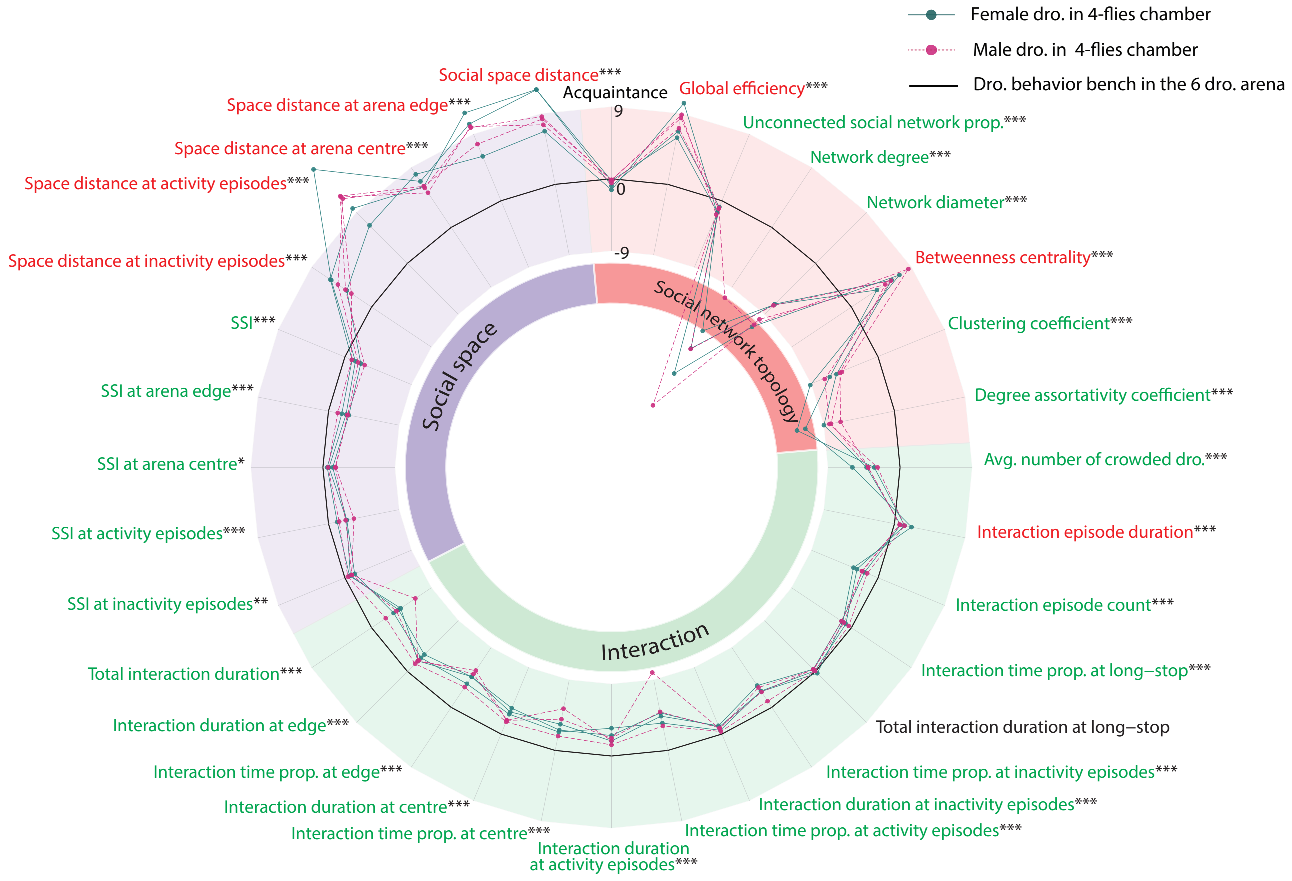
Female dro. in 4-flies chamber
Male dro. in 4-flies chamber
Dro. behavior bench in the 6 dro. arena
Acquaintance
9
0
-9
Social space distance***
Space distance at arena edge***
Space distance at arena centre***
Space distance at activity episodes***
Space distance at inactivity episodes***
SSI***
SSI at arena edge***
SSI at arena centre*
SSI at activity episodes***
SSI at inactivity episodes**
Total interaction duration***
Interaction duration at edge***
Interaction time prop. at edge***
Interaction duration at centre***
Interaction time prop. at centre***
Interaction duration at activity episodes***
Interaction time prop. at activity episodes***
Interaction duration at inactivity episodes***
Interaction time prop. at inactivity episodes***
Total interaction duration at long–stop
Interaction time prop. at long–stop***
Interaction episode count***
Interaction episode duration***
Avg. number of crowded dro.***
Degree assortativity coefficient***
Clustering coefficient***
Betweenness centrality***
Network diameter***
Network degree***
Unconnected social network prop.***
Global efficiency***
Social space
Social network topology
Interaction

C

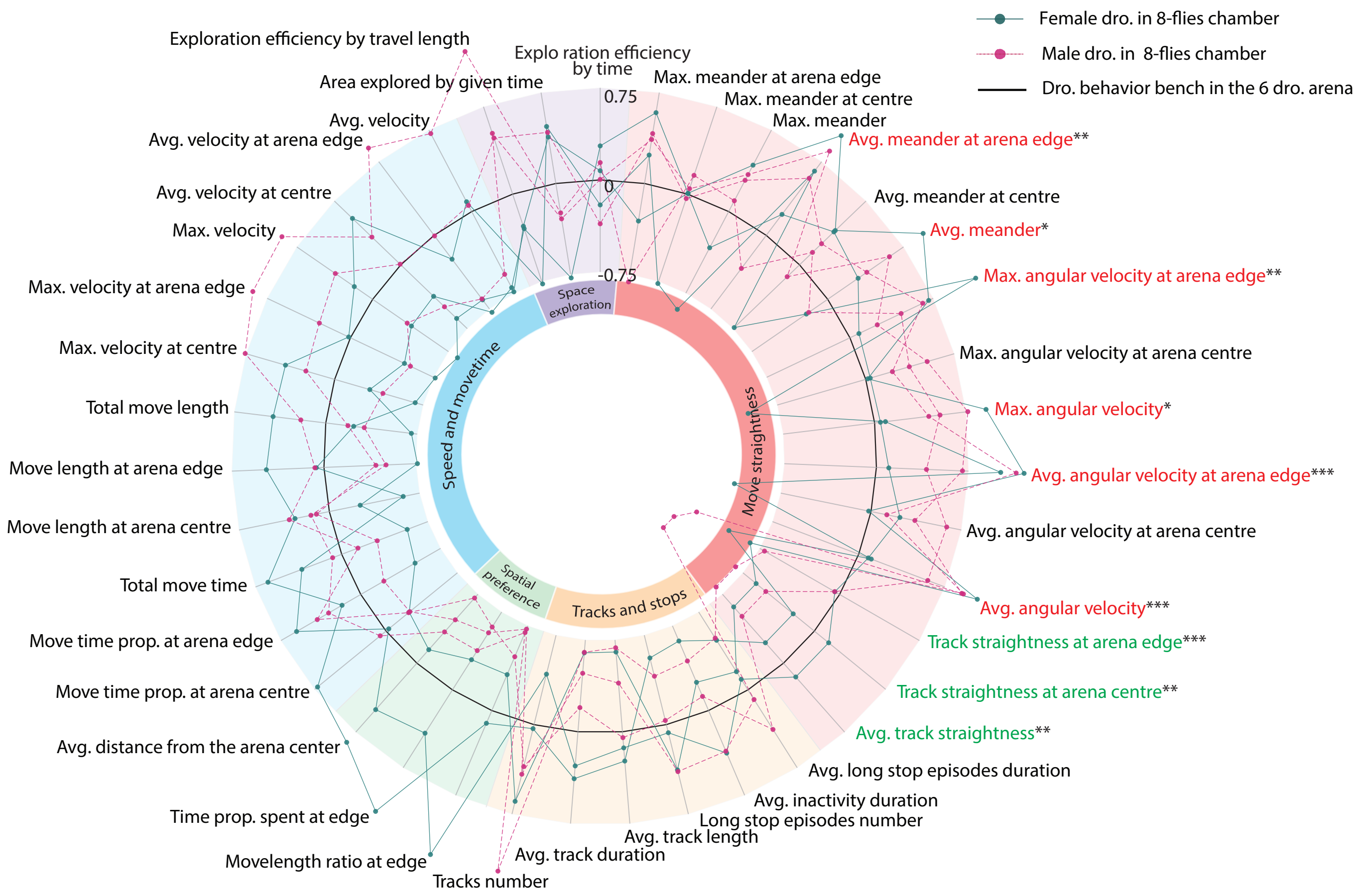

Female dro. in 8-flies chamber
Male dro. in 8-flies chamber
Dro. behavior bench in the 6 dro. arena
0.75
0
-0.75
Space exploration
Move straightness
Tracks and stops
Spatial preference
Speed and movetime
Exploration efficiency by travel length
Explo ration efficiency by time
Area explored by given time
Avg. velocity
Avg. velocity at arena edge
Avg. velocity at centre
Max. velocity
Max. velocity at arena edge
Max. velocity at centre
Total move length
Move length at arena edge
Move length at arena centre
Total move time
Move time prop. at arena edge
Move time prop. at arena centre
Avg. distance from the arena center
Time prop. spent at edge
Movelength ratio at edge
Tracks number
Avg. track duration
Avg. track length
Long stop episodes number
Avg. inactivity duration
Avg. long stop episodes duration
Avg. track straightness**
Track straightness at arena centre**
Track straightness at arena edge***
Avg. angular velocity***
Avg. angular velocity at arena centre
Avg. angular velocity at arena edge***
Max. angular velocity*
Max. angular velocity at arena centre
Max. angular velocity at arena edge**
Avg. meander*
Avg. meander at centre
Avg. meander at arena edge**
Max. meander
Max. meander at centre
Max. meander at arena edge

D

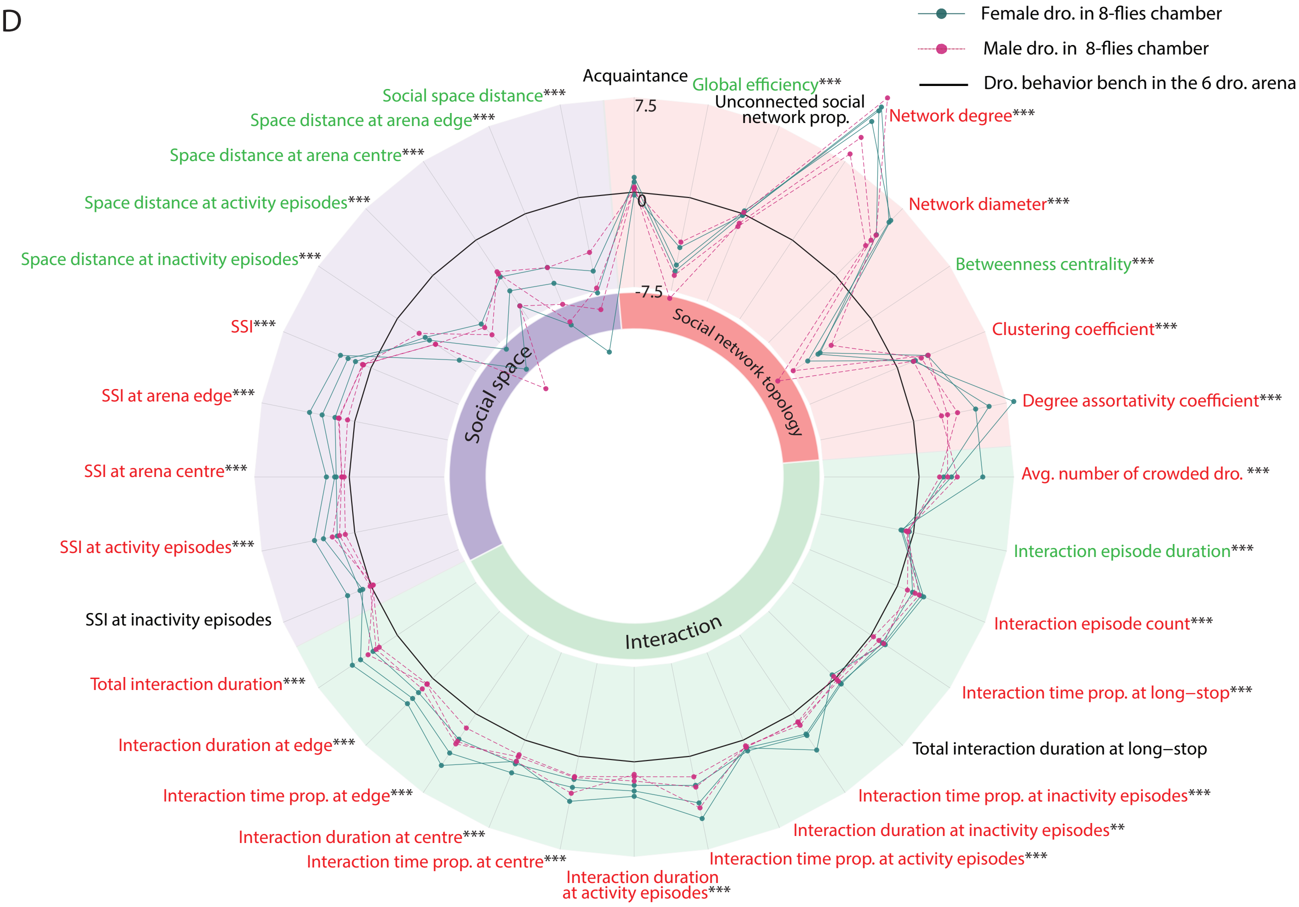

Female dro. in 8-flies chamber
Male dro. in 8-flies chamber
Dro. behavior bench in the 6 dro. arena
7.5
0
-7.5
Social network topology
Interaction
Social space
Acquaintance
Global efficiency***
Unconnected social network prop.
Network degree***
Network diameter***
Betweenness centrality***
Clustering coefficient***
Degree assortativity coefficient***
Avg. number of crowded dro. ***
Interaction episode duration***
Interaction episode count***
Interaction time prop. at long−stop***
Total interaction duration at long−stop
Interaction time prop. at inactivity episodes***
Interaction duration at inactivity episodes**
Interaction time prop. at activity episodes***
Interaction duration at activity episodes***
Interaction time prop. at centre***
Interaction duration at centre***
Interaction time prop. at edge***
Interaction duration at edge***
Total interaction duration***
SSI at inactivity episodes
SSI at activity episodes***
SSI at arena centre***
SSI at arena edge***
SSI***
Space distance at inactivity episodes***
Space distance at activity episodes***
Space distance at arena centre***
Space distance at arena edge***
Social space distance***

Figure S4

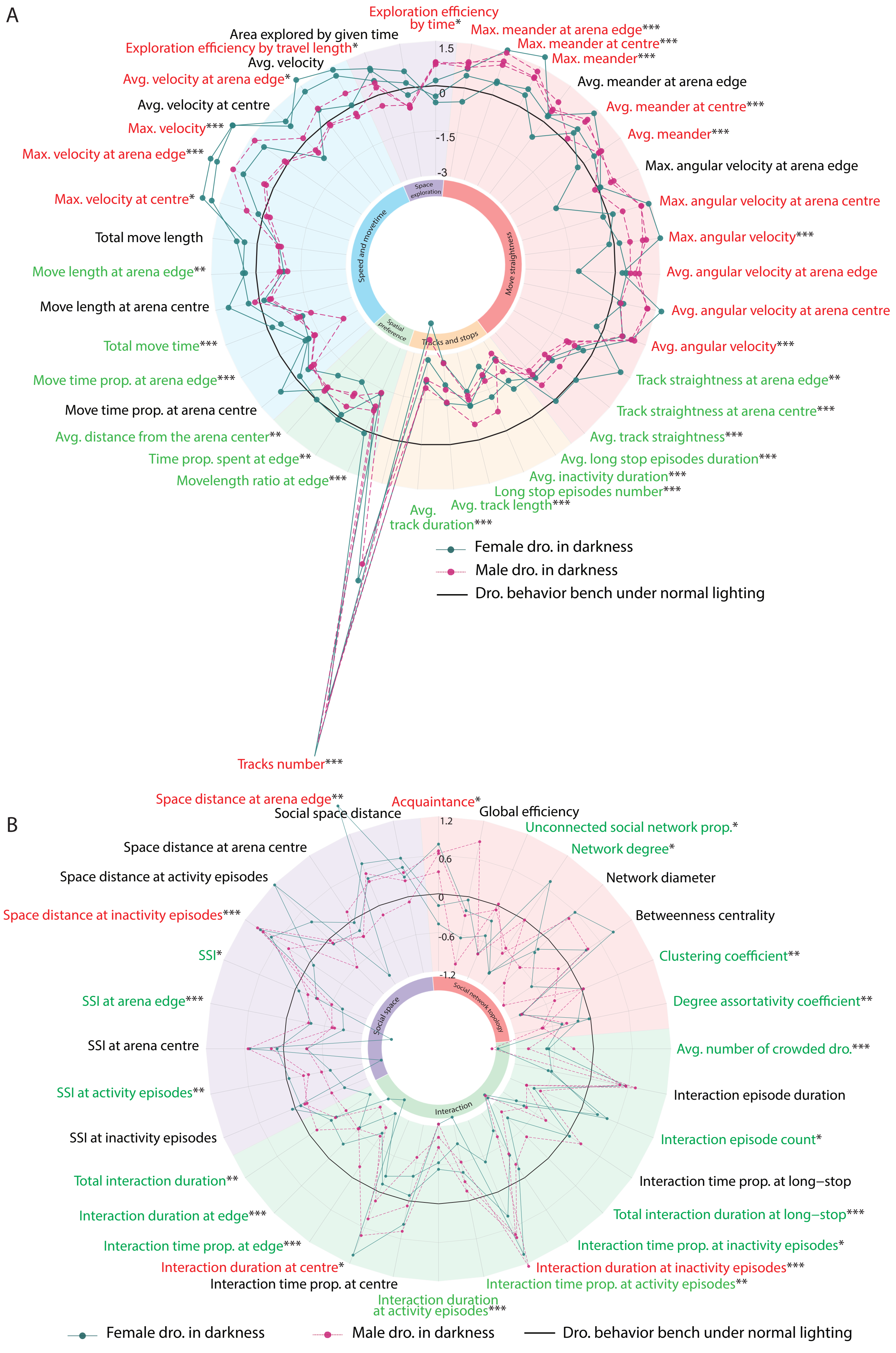
A
Exploration efficiency by time*
Area explored by given time
Exploration efficiency by travel length*
Avg. velocity
Avg. velocity at arena edge*
Avg. velocity at centre
Max. velocity***
Max. velocity at arena edge***
Max. velocity at centre*
Total move length
Move length at arena edge**
Move length at arena centre
Total move time***
Move time prop. at arena edge***
Move time prop. at arena centre
Avg. distance from the arena center**
Time prop. spent at edge**
Movelength ratio at edge***
Tracks number***
Avg. track duration***
Avg. track length***
Long stop episodes number***
Avg. inactivity duration***
Avg. long stop episodes duration***
Avg. track straightness***
Track straightness at arena centre***
Track straightness at arena edge**
Avg. angular velocity***
Avg. angular velocity at arena centre
Avg. angular velocity at arena edge
Max. angular velocity***
Max. angular velocity at arena centre
Max. angular velocity at arena edge
Avg. meander***
Avg. meander at centre***
Avg. meander at arena edge
Max. meander***
Max. meander at centre***
Max. meander at arena edge***
1.5
0
-1.5
-3
Space exploration
Speed and movetime
Move straightness
Spatial preference
Tracks and stops
Female dro. in darkness
Male dro. in darkness
Dro. behavior bench under normal lighting
B
Space distance at arena edge**
Social space distance
Acquaintance*
Global efficiency
Unconnected social network prop.*
Network degree*
Network diameter
Betweenness centrality
Clustering coefficient**
Degree assortativity coefficient**
Avg. number of crowded dro.***
Interaction episode duration
Interaction episode count*
Interaction time prop. at long–stop
Total interaction duration at long–stop***
Interaction time prop. at inactivity episodes*
Interaction duration at inactivity episodes***
Interaction time prop. at activity episodes**
Interaction duration at activity episodes***
Interaction time prop. at centre
Interaction duration at centre*
Interaction time prop. at edge***
Interaction duration at edge***
Total interaction duration**
SSI at inactivity episodes
SSI at activity episodes**
SSI at arena centre
SSI at arena edge***
SSI*
Space distance at inactivity episodes***
Space distance at activity episodes
Space distance at arena centre
1.2
0.6
0
-0.6
-1.2
Social space
Social network topology
Interaction
Female dro. in darkness
Male dro. in darkness
Dro. behavior bench under normal lighting

Figure S5

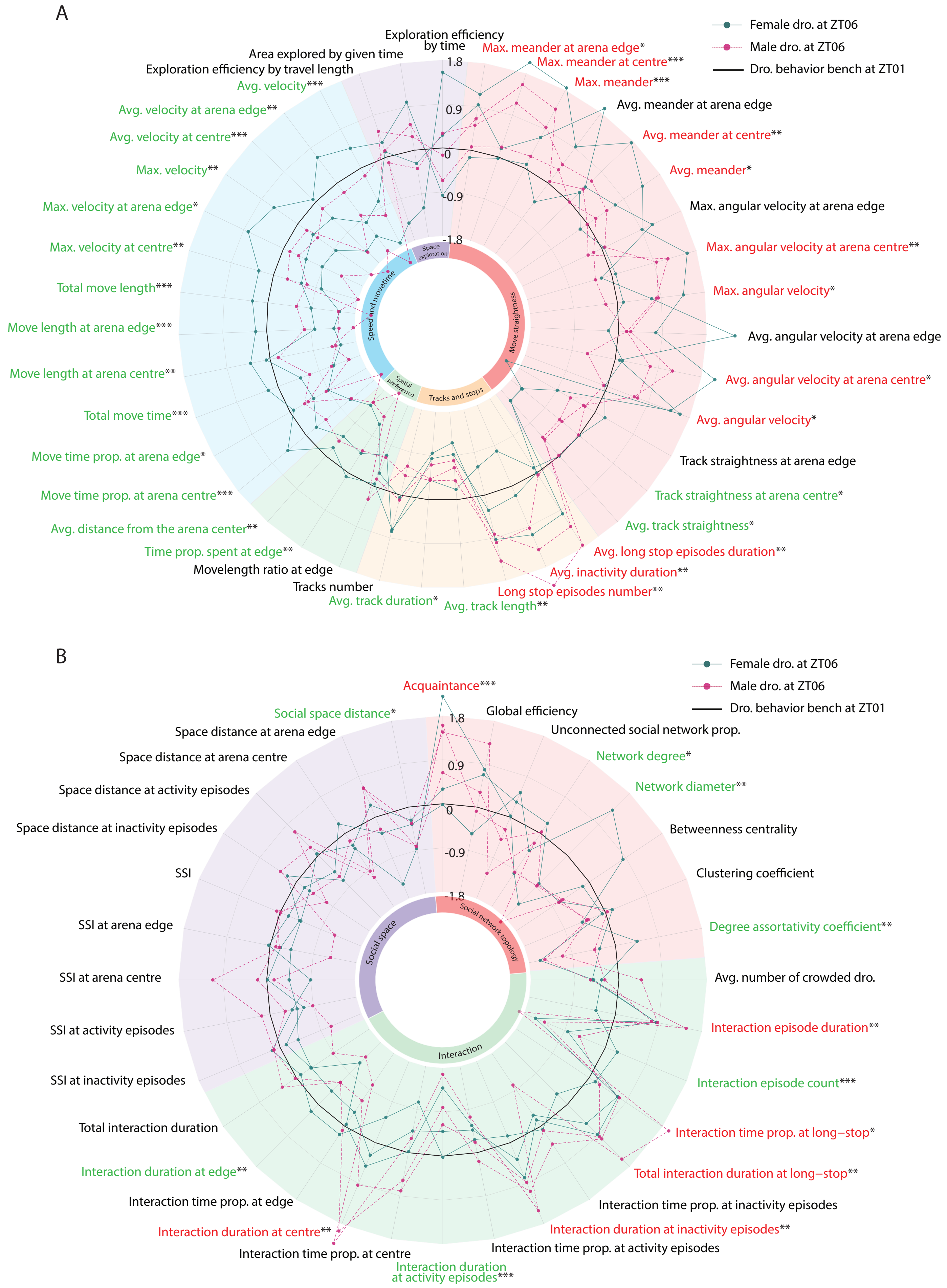
A
Female dro. at ZT06
Male dro. at ZT06
Dro. behavior bench at ZT01
Exploration efficiency by time
Area explored by given time
Exploration efficiency by travel length
Avg. velocity***
Avg. velocity at arena edge**
Avg. velocity at centre***
Max. velocity**
Max. velocity at arena edge*
Max. velocity at centre**
Total move length***
Move length at arena edge***
Move length at arena centre**
Total move time***
Move time prop. at arena edge*
Move time prop. at arena centre***
Avg. distance from the arena center**
Time prop. spent at edge**
Movelength ratio at edge
Tracks number
Avg. track duration*
Avg. track length**
Long stop episodes number**
Avg. inactivity duration**
Avg. long stop episodes duration**
Avg. track straightness*
Track straightness at arena centre*
Track straightness at arena edge
Avg. angular velocity*
Avg. angular velocity at arena centre*
Avg. angular velocity at arena edge
Max. angular velocity*
Max. angular velocity at arena centre**
Max. angular velocity at arena edge
Avg. meander*
Avg. meander at centre**
Avg. meander at arena edge
Max. meander***
Max. meander at centre***
Max. meander at arena edge*
1.8
0.9
0
-0.9
-1.8
Space exploration
Speed and movetime
Spatial preference
Tracks and stops
Move straightness
B
Female dro. at ZT06
Male dro. at ZT06
Dro. behavior bench at ZT01
Acquaintance***
Global efficiency
Unconnected social network prop.
Network degree*
Network diameter**
Betweenness centrality
Clustering coefficient
Degree assortativity coefficient**
Avg. number of crowded dro.
Interaction episode duration**
Interaction episode count***
Interaction time prop. at long–stop*
Total interaction duration at long–stop**
Interaction time prop. at inactivity episodes
Interaction duration at inactivity episodes**
Interaction time prop. at activity episodes
Interaction duration at activity episodes***
Interaction time prop. at centre
Interaction duration at centre**
Interaction time prop. at edge
Interaction duration at edge**
Total interaction duration
SSI at inactivity episodes
SSI at activity episodes
SSI at arena centre
SSI at arena edge
SSI
Space distance at inactivity episodes
Space distance at activity episodes
Space distance at arena centre
Space distance at arena edge
Social space distance*
1.8
0.9
0
-0.9
-1.8
Social network topology
Social space
Interaction

C

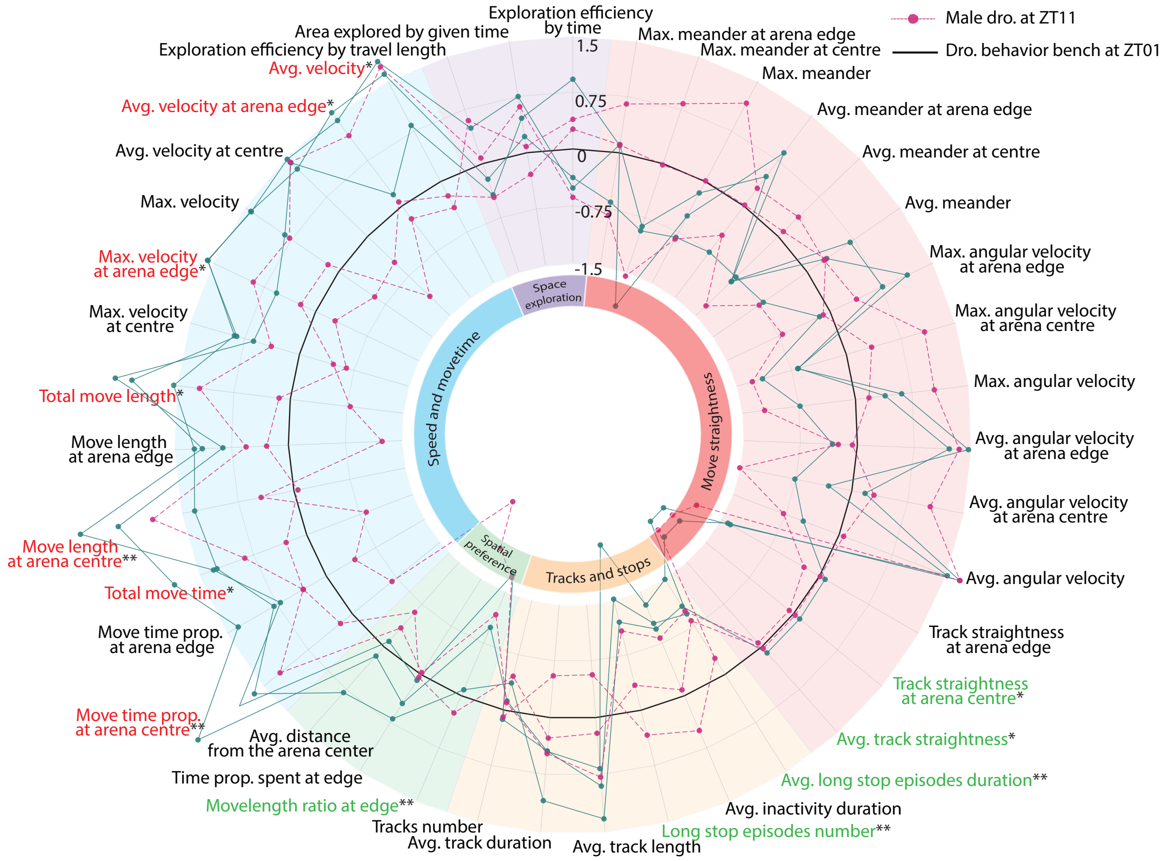

Female dro. at ZT11
Male dro. at ZT11
Dro. behavior bench at ZT01
Exploration efficiency by time
Area explored by given time
Exploration efficiency by travel length
Avg. velocity*
Avg. velocity at arena edge*
Avg. velocity at centre
Max. velocity
Max. velocity at arena edge*
Max. velocity at centre
Total move length*
Move length at arena edge
Move length at arena centre**
Total move time*
Move time prop. at arena edge
Move time prop. at arena centre**
Avg. distance from the arena center
Time prop. spent at edge
Movelength ratio at edge**
Tracks number
Avg. track duration
Avg. track length
Long stop episodes number**
Avg. inactivity duration
Avg. long stop episodes duration**
Avg. track straightness*
Track straightness at arena centre*
Track straightness at arena edge
Avg. angular velocity
Avg. angular velocity at arena centre
Avg. angular velocity at arena edge
Max. angular velocity
Max. angular velocity at arena centre
Max. angular velocity at arena edge
Avg. meander
Avg. meander at centre
Avg. meander at arena edge
Max. meander
Max. meander at centre
Max. meander at arena edge
1.5
0.75
0
-0.75
-1.5
Space exploration
Speed and movetime
Move straightness
Tracks and stops
Spatial preference

D

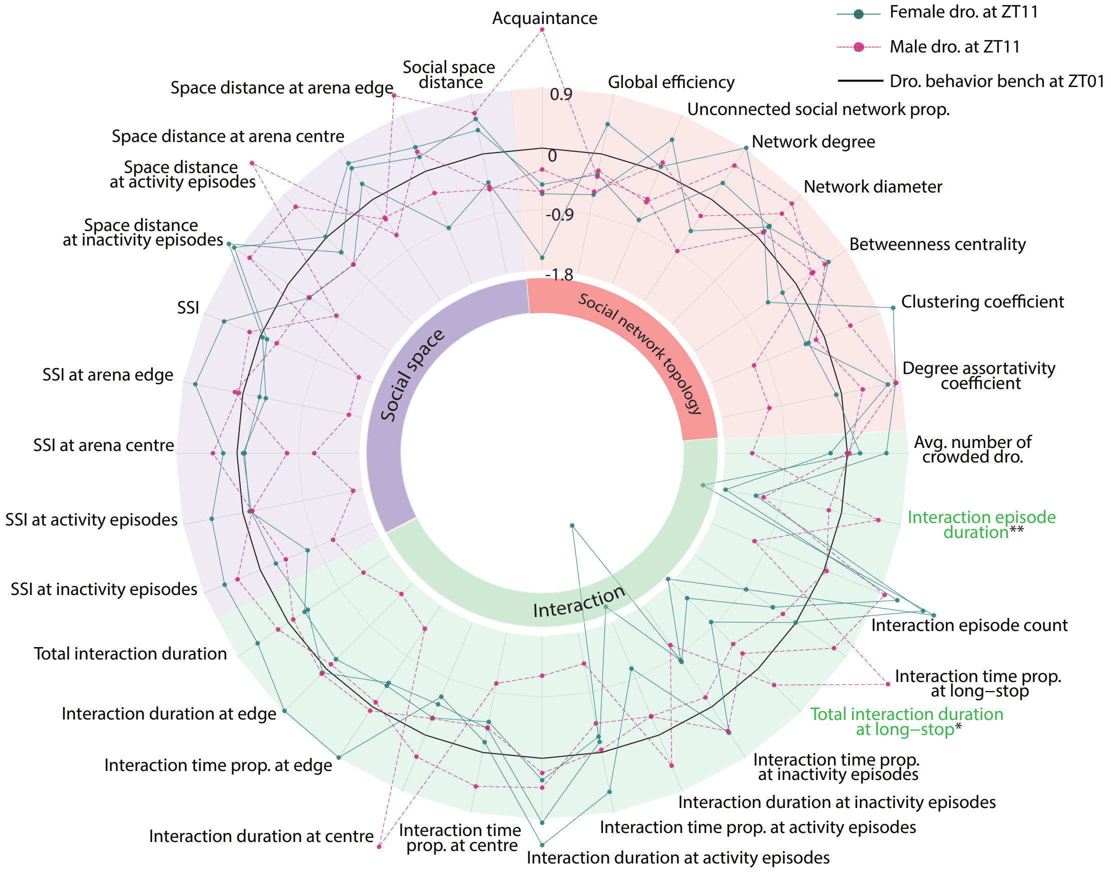

Female dro. at ZT11
Male dro. at ZT11
Dro. behavior bench at ZT01
Acquaintance
Social space distance
Space distance at arena edge
Space distance at arena centre
Space distance at activity episodes
Space distance at inactivity episodes
SSI
SSI at arena edge
SSI at arena centre
SSI at activity episodes
SSI at inactivity episodes
Total interaction duration
Interaction duration at edge
Interaction time prop. at edge
Interaction duration at centre
Interaction time prop. at centre
Interaction duration at activity episodes
Interaction time prop. at activity episodes
Interaction duration at inactivity episodes
Interaction time prop. at inactivity episodes
Total interaction duration at long−stop*
Interaction time prop. at long−stop
Interaction episode count
Interaction episode duration**
Avg. number of crowded dro.
Degree assortativity coefficient
Clustering coefficient
Betweenness centrality
Network diameter
Network degree
Unconnected social network prop.
Global efficiency
0.9
0
-0.9
-1.8
Social space
Social network topology
Interaction

Figure S6

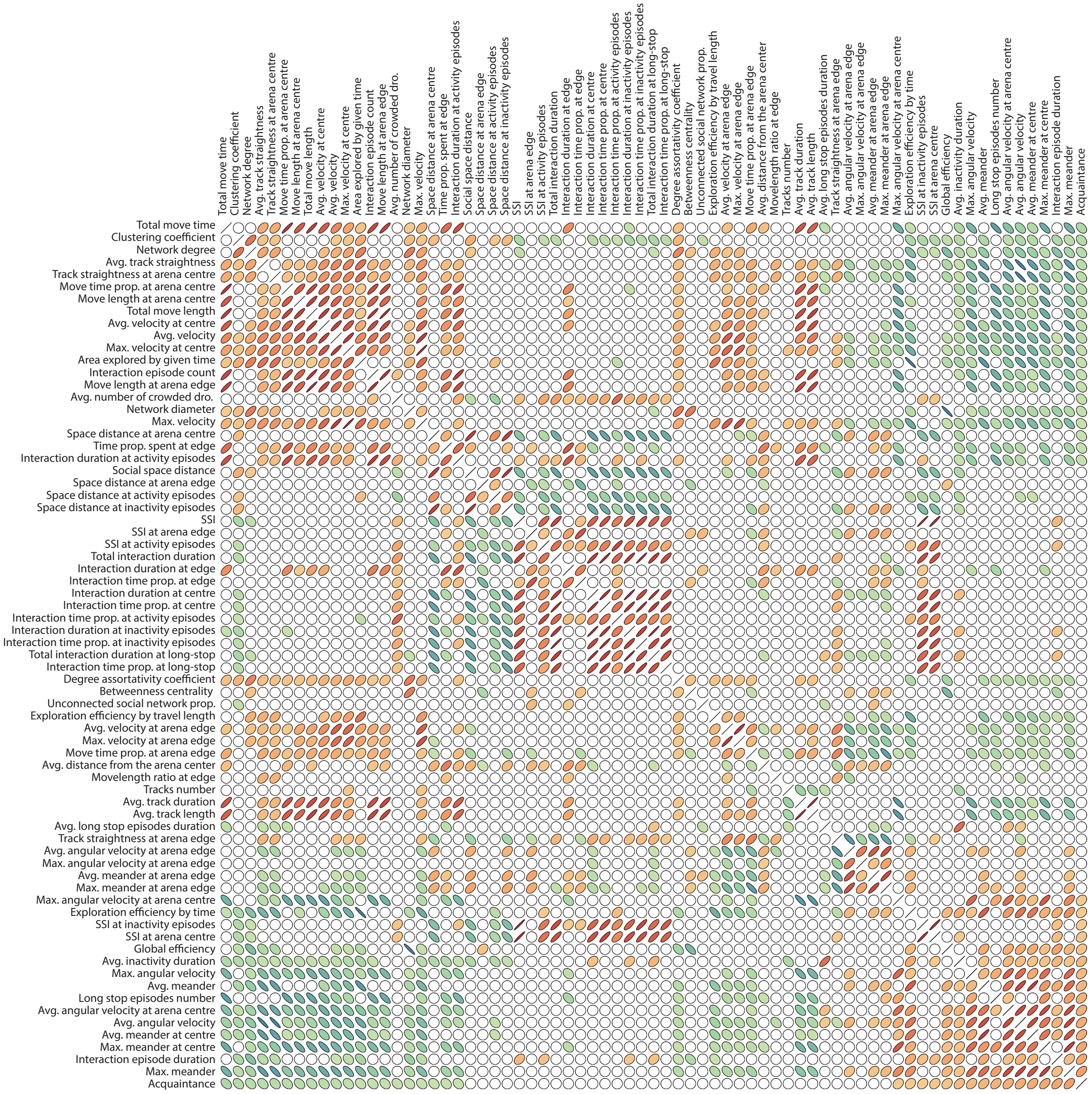
Total move time
Clustering coefficient
Network degree
Avg. track straightness
Track straightness at arena centre
Move time prop. at arena centre
Move length at arena centre
Total move length
Avg. velocity at centre
Avg. velocity
Max. velocity at centre
Area explored by given time
Interaction episode count
Move length at arena edge
Avg. number of crowded dro.
Network diameter
Max. velocity
Space distance at arena centre
Time prop. spent at edge
Interaction duration at activity episodes
Social space distance
Space distance at arena edge
Space distance at activity episodes
Space distance at inactivity episodes
SSI
SSI at arena edge
SSI at activity episodes
Total interaction duration
Interaction duration at edge
Interaction time prop. at edge
Interaction duration at centre
Interaction time prop. at centre
Interaction time prop. at activity episodes
Interaction duration at inactivity episodes
Interaction time prop. at inactivity episodes
Total interaction duration at long-stop
Interaction time prop. at long-stop
Degree assortativity coefficient
Betweenness centrality
Unconnected social network prop.
Exploration efficiency by travel length
Avg. velocity at arena edge
Max. velocity at arena edge
Move time prop. at arena edge
Avg. distance from the arena center
Movelength ratio at edge
Tracks number
Avg. track duration
Avg. track length
Avg. long stop episodes duration
Track straightness at arena edge
Avg. angular velocity at arena edge
Max. angular velocity at arena edge
Avg. meander at arena edge
Max. meander at arena edge
Max. angular velocity at arena centre
Exploration efficiency by time
SSI at inactivity episodes
SSI at arena centre
Global efficiency
Avg. inactivity duration
Max. angular velocity
Avg. meander
Long stop episodes number
Avg. angular velocity at arena centre
Avg. angular velocity
Avg. meander at centre
Max. meander at centre
Interaction episode duration
Max. meander
Acquaintance

Figur S7

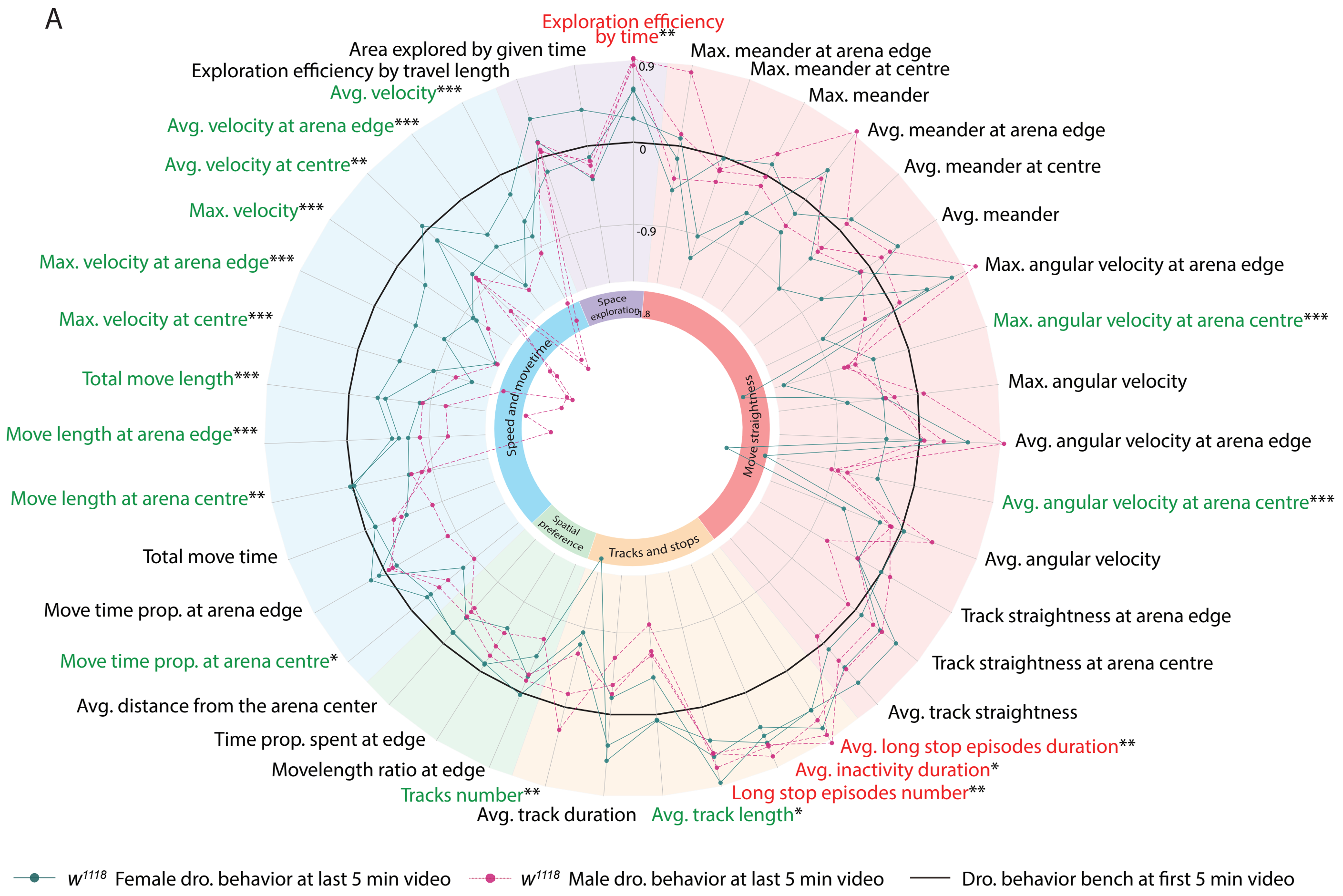
A
Exploration efficiency by time**
Area explored by given time
Exploration efficiency by travel length
Avg. velocity***
Avg. velocity at arena edge***
Avg. velocity at centre**
Max. velocity***
Max. velocity at arena edge***
Max. velocity at centre***
Total move length***
Move length at arena edge***
Move length at arena centre**
Total move time
Move time prop. at arena edge
Move time prop. at arena centre*
Avg. distance from the arena center
Time prop. spent at edge
Movelength ratio at edge
Tracks number**
Avg. track duration
Avg. track length*
Long stop episodes number**
Avg. inactivity duration*
Avg. long stop episodes duration**
Avg. track straightness
Track straightness at arena centre
Track straightness at arena edge
Avg. angular velocity
Avg. angular velocity at arena centre***
Avg. angular velocity at arena edge
Max. angular velocity
Max. angular velocity at arena centre***
Max. angular velocity at arena edge
Avg. meander
Avg. meander at centre
Avg. meander at arena edge
Max. meander
Max. meander at centre
Max. meander at arena edge
0.9
0
-0.9
Space exploration
Speed and movetime
Spatial preference
Tracks and stops
Move straightness
w1118 Female dro. behavior at last 5 min video
w1118 Male dro. behavior at last 5 min video
Dro. behavior bench at first 5 min video

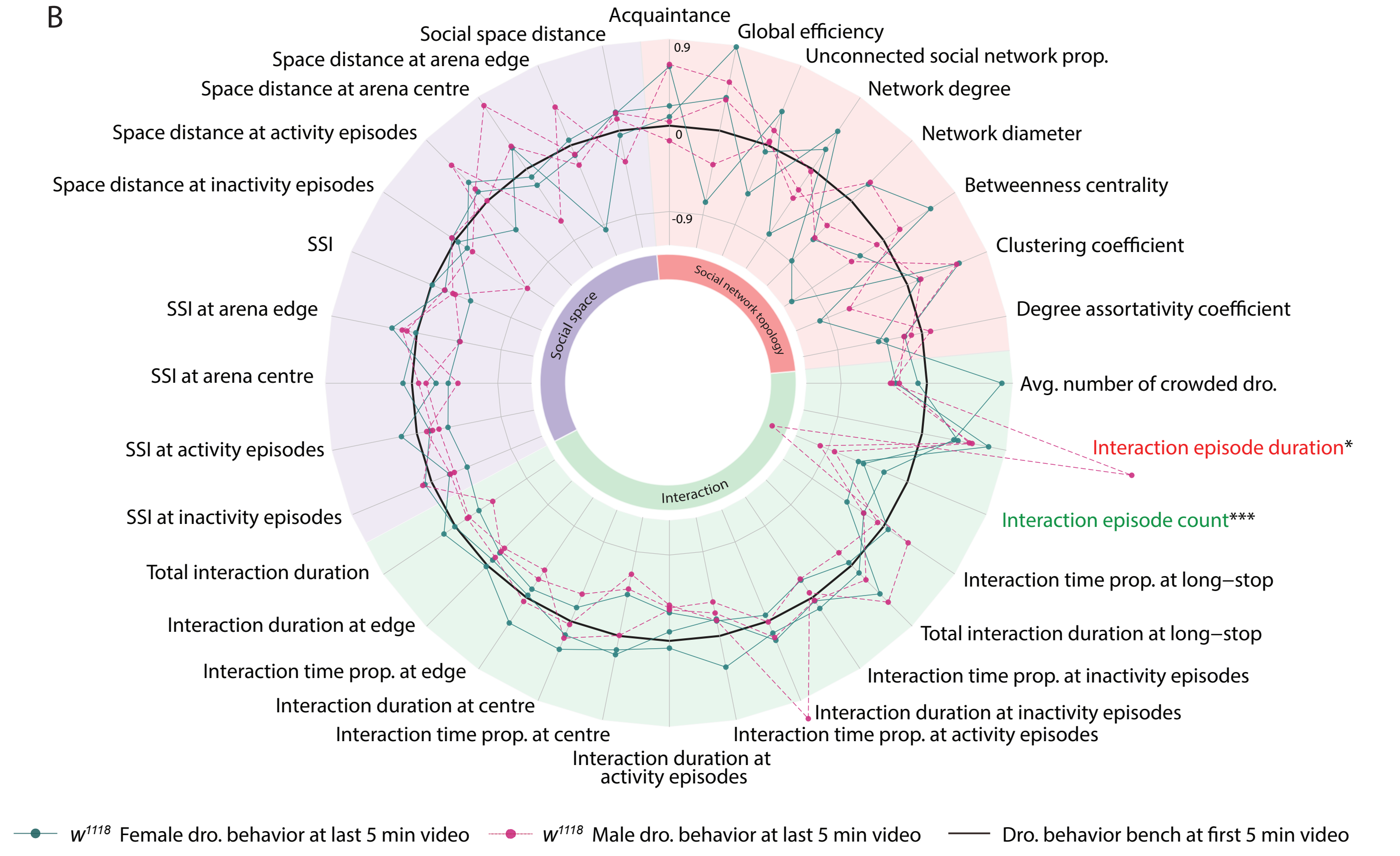
B
Acquaintance
Social space distance
Space distance at arena edge
Space distance at arena centre
Space distance at activity episodes
Space distance at inactivity episodes
SSI
SSI at arena edge
SSI at arena centre
SSI at activity episodes
SSI at inactivity episodes
Total interaction duration
Interaction duration at edge
Interaction time prop. at edge
Interaction duration at centre
Interaction time prop. at centre
Interaction duration at activity episodes
Interaction time prop. at activity episodes
Interaction duration at inactivity episodes
Interaction time prop. at inactivity episodes
Total interaction duration at long–stop
Interaction time prop. at long–stop
Interaction episode count***
Interaction episode duration*
Avg. number of crowded dro.
Degree assortativity coefficient
Clustering coefficient
Betweenness centrality
Network diameter
Network degree
Unconnected social network prop.
Global efficiency
0.9
0
-0.9
Social space
Social network topology
Interaction
w1118 Female dro. behavior at last 5 min video
w1118 Male dro. behavior at last 5 min video
Dro. behavior bench at first 5 min video

C

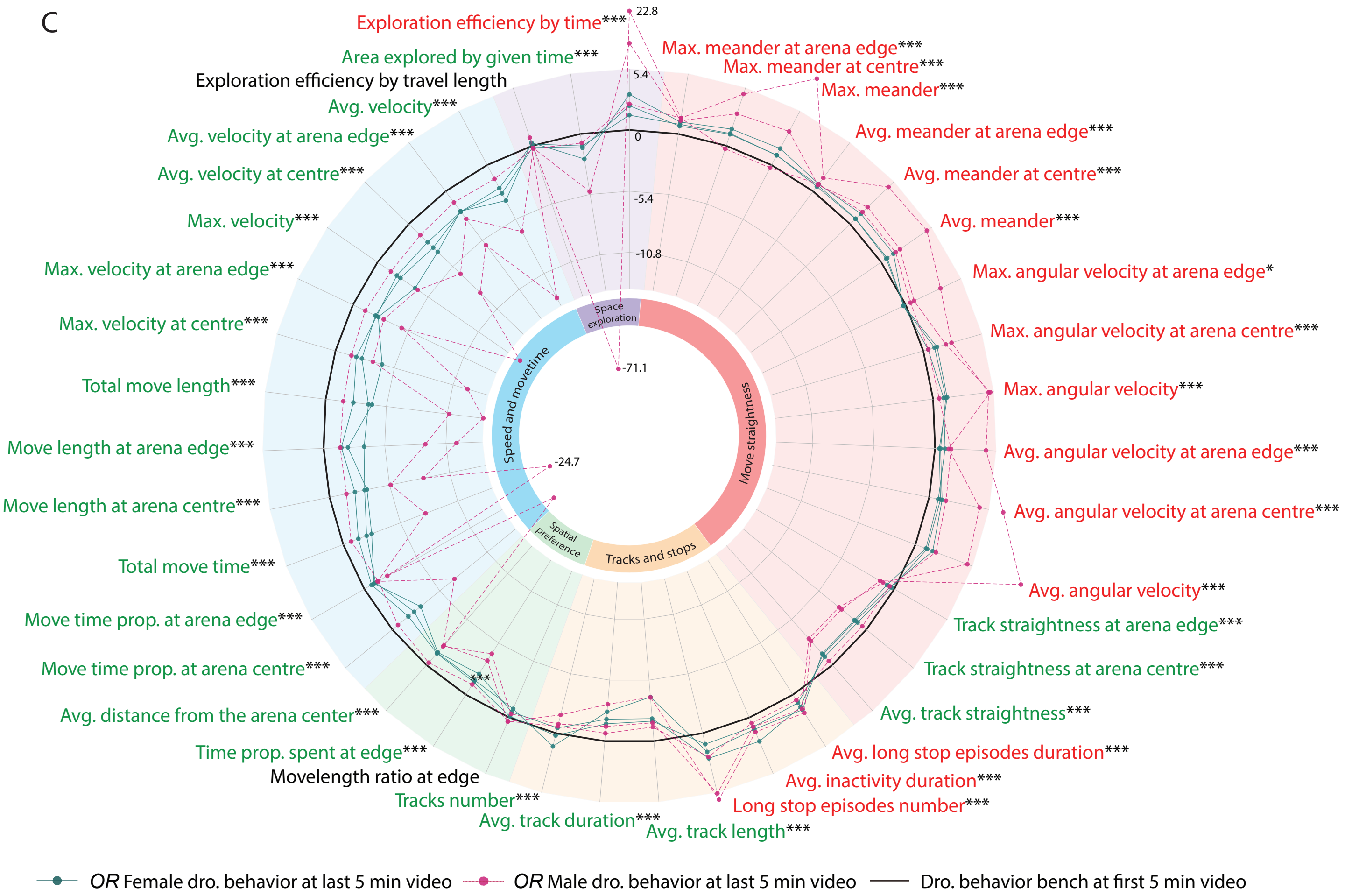
Exploration efficiency by time***
Area explored by given time***
Exploration efficiency by travel length
Avg. velocity***
Avg. velocity at arena edge***
Avg. velocity at centre***
Max. velocity***
Max. velocity at arena edge***
Max. velocity at centre***
Total move length***
Move length at arena edge***
Move length at arena centre***
Total move time***
Move time prop. at arena edge***
Move time prop. at arena centre***
Avg. distance from the arena center***
Time prop. spent at edge***
Movelength ratio at edge
Tracks number***
Avg. track duration***
Avg. track length***
Long stop episodes number***
Avg. inactivity duration***
Avg. long stop episodes duration***
Avg. track straightness***
Track straightness at arena centre***
Track straightness at arena edge***
Avg. angular velocity***
Avg. angular velocity at arena centre***
Avg. angular velocity at arena edge***
Max. angular velocity***
Max. angular velocity at arena centre***
Max. angular velocity at arena edge*
Avg. meander***
Avg. meander at centre***
Avg. meander at arena edge***
Max. meander***
Max. meander at centre***
Max. meander at arena edge***
22.8
5.4
0
-5.4
-10.8
-71.1
-24.7
***
Space exploration
Speed and movetime
Spatial preference
Tracks and stops
Move straightness
OR Female dro. behavior at last 5 min video
OR Male dro. behavior at last 5 min video
Dro. behavior bench at first 5 min video

D

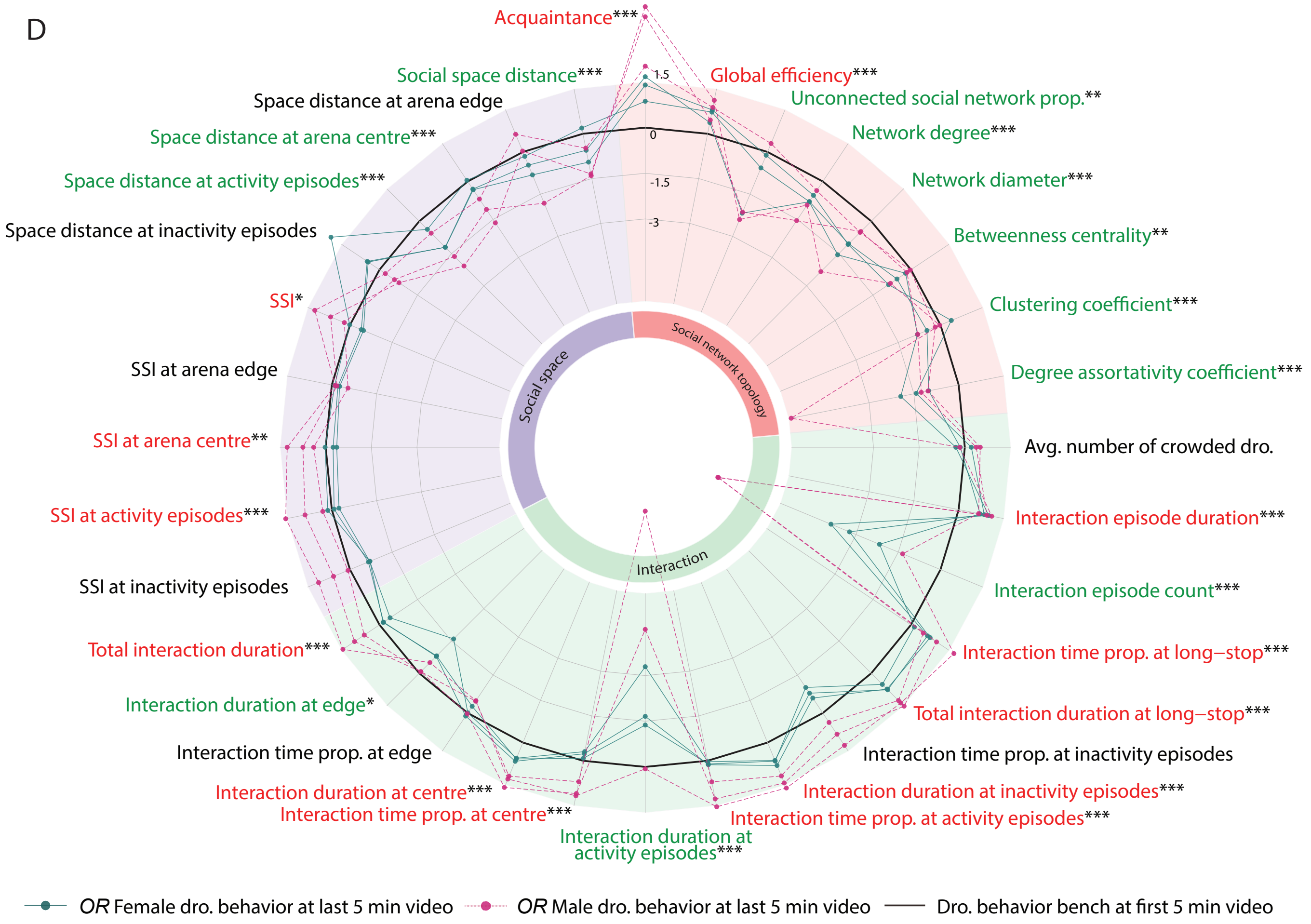
Acquaintance***
Social space distance***
Space distance at arena edge
Space distance at arena centre***
Space distance at activity episodes***
Space distance at inactivity episodes
SSI*
SSI at arena edge
SSI at arena centre**
SSI at activity episodes***
SSI at inactivity episodes
Total interaction duration***
Interaction duration at edge*
Interaction time prop. at edge
Interaction duration at centre***
Interaction time prop. at centre***
Interaction duration at
activity episodes***
Interaction time prop. at activity episodes***
Interaction duration at inactivity episodes***
Interaction time prop. at inactivity episodes
Total interaction duration at long–stop***
Interaction time prop. at long–stop***
Interaction episode count***
Interaction episode duration***
Avg. number of crowded dro.
Degree assortativity coefficient***
Clustering coefficient***
Betweenness centrality**
Network diameter***
Network degree***
Unconnected social network prop.**
Global efficiency***
1.5
0
-1.5
-3
Social space
Social network topology
Interaction
OR Female dro. behavior at last 5 min video
OR Male dro. behavior at last 5 min video
Dro. behavior bench at first 5 min video

E

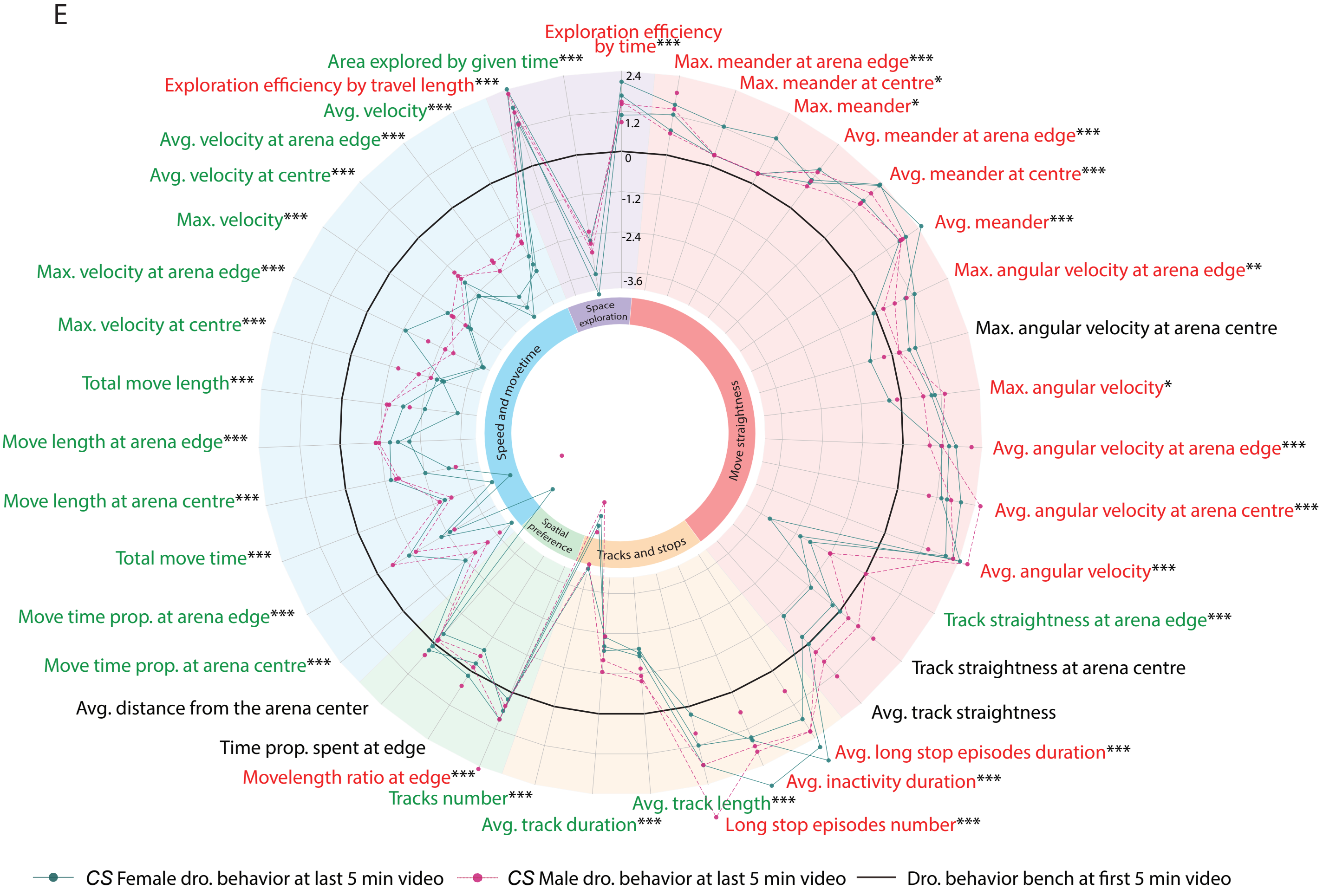
Exploration efficiency by time***
Area explored by given time***
Exploration efficiency by travel length***
Avg. velocity***
Avg. velocity at arena edge***
Avg. velocity at centre***
Max. velocity***
Max. velocity at arena edge***
Max. velocity at centre***
Total move length***
Move length at arena edge***
Move length at arena centre***
Total move time***
Move time prop. at arena edge***
Move time prop. at arena centre***
Avg. distance from the arena center
Time prop. spent at edge
Movelength ratio at edge***
Tracks number***
Avg. track duration***
Avg. track length***
Long stop episodes number***
Avg. inactivity duration***
Avg. long stop episodes duration***
Avg. track straightness
Track straightness at arena centre
Track straightness at arena edge***
Avg. angular velocity***
Avg. angular velocity at arena centre***
Avg. angular velocity at arena edge***
Max. angular velocity*
Max. angular velocity at arena centre
Max. angular velocity at arena edge**
Avg. meander***
Avg. meander at centre***
Avg. meander at arena edge***
Max. meander*
Max. meander at centre*
Max. meander at arena edge***
2.4
1.2
0
-1.2
-2.4
-3.6
Space exploration
Move straightness
Tracks and stops
Spatial preference
Speed and movetime
CS Female dro. behavior at last 5 min video
CS Male dro. behavior at last 5 min video
Dro. behavior bench at first 5 min video

F

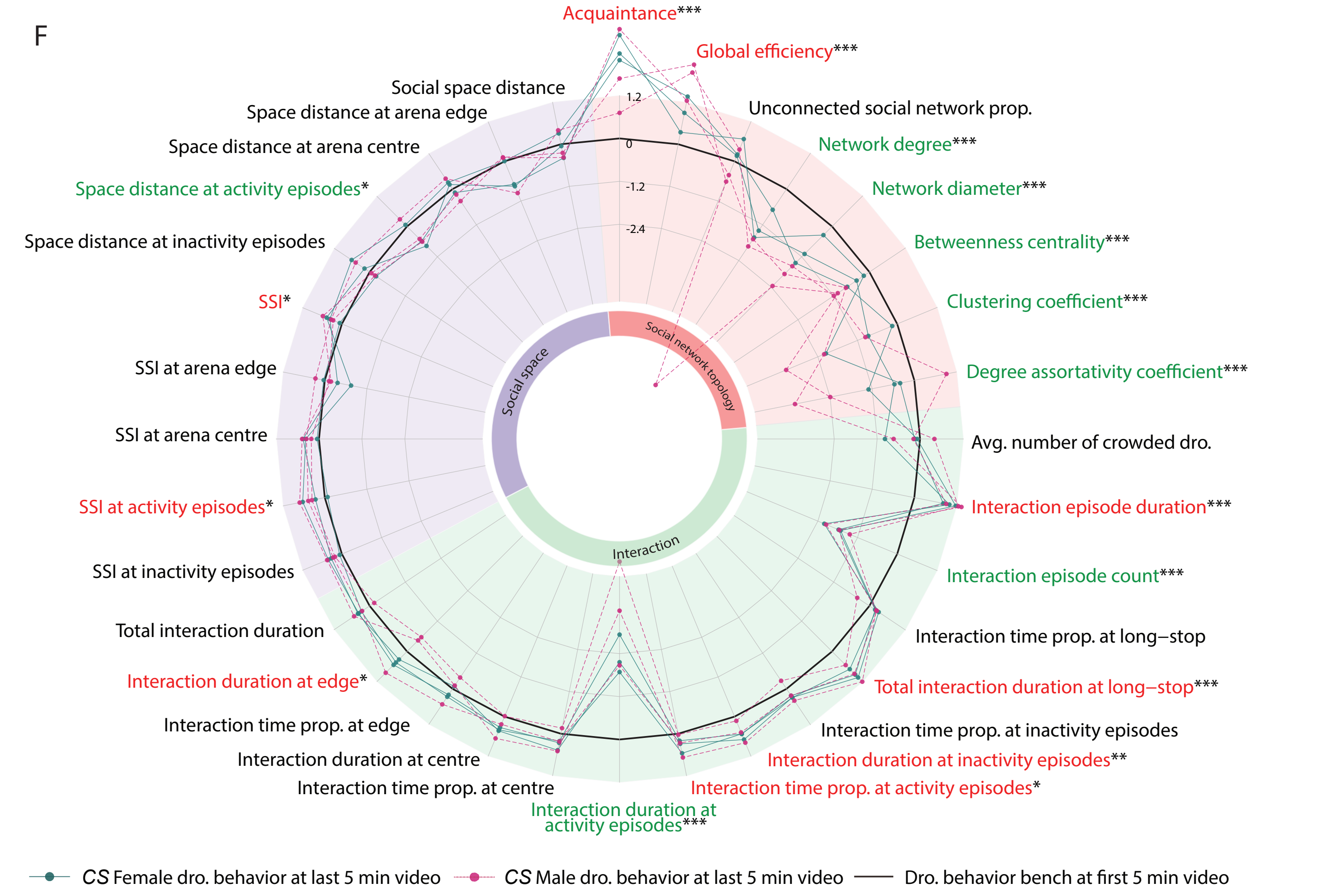
Acquaintance***
Global efficiency***
Unconnected social network prop.
Network degree***
Network diameter***
Betweenness centrality***
Clustering coefficient***
Degree assortativity coefficient***
Avg. number of crowded dro.
Interaction episode duration***
Interaction episode count***
Interaction time prop. at long–stop
Total interaction duration at long–stop***
Interaction time prop. at inactivity episodes
Interaction duration at inactivity episodes**
Interaction time prop. at activity episodes*
Interaction duration at activity episodes***
Interaction time prop. at centre
Interaction duration at centre
Interaction time prop. at edge
Interaction duration at edge*
Total interaction duration
SSI at inactivity episodes
SSI at activity episodes*
SSI at arena centre
SSI at arena edge
SSI*
Space distance at inactivity episodes
Space distance at activity episodes*
Space distance at arena centre
Space distance at arena edge
Social space distance
1.2
0
-1.2
-2.4
Social network topology
Social space
Interaction
CS Female dro. behavior at last 5 min video
CS Male dro. behavior at last 5 min video
Dro. behavior bench at first 5 min video

Figure S8

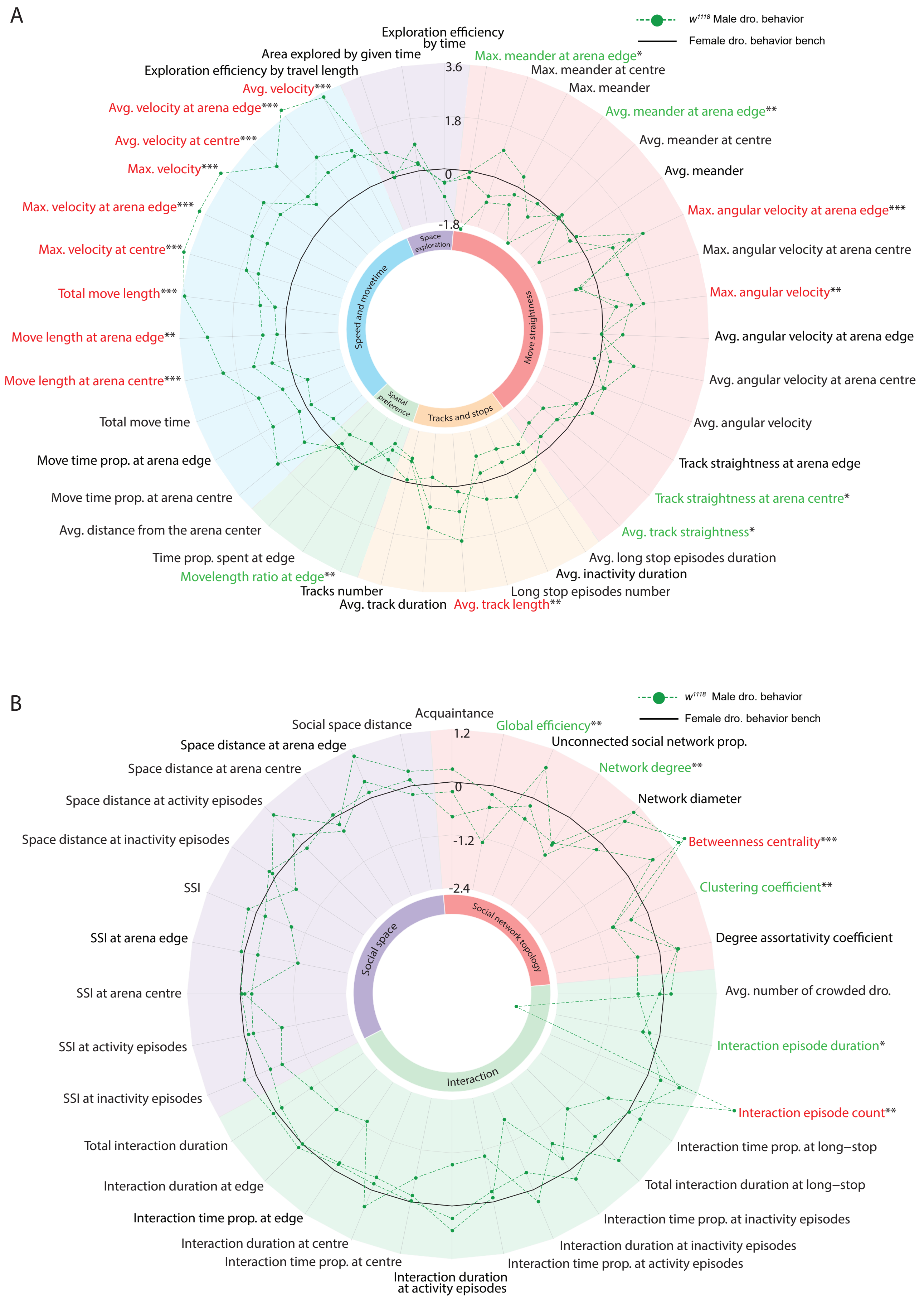
A
w1118 Male dro. behavior
Female dro. behavior bench
Exploration efficiency by time
Area explored by given time
Exploration efficiency by travel length
Avg. velocity***
Avg. velocity at arena edge***
Avg. velocity at centre***
Max. velocity***
Max. velocity at arena edge***
Max. velocity at centre***
Total move length***
Move length at arena edge**
Move length at arena centre***
Total move time
Move time prop. at arena edge
Move time prop. at arena centre
Avg. distance from the arena center
Time prop. spent at edge
Movelength ratio at edge**
Tracks number
Avg. track duration
Avg. track length**
Long stop episodes number
Avg. inactivity duration
Avg. long stop episodes duration
Avg. track straightness*
Track straightness at arena centre*
Track straightness at arena edge
Avg. angular velocity
Avg. angular velocity at arena centre
Avg. angular velocity at arena edge
Max. angular velocity**
Max. angular velocity at arena centre
Max. angular velocity at arena edge***
Avg. meander
Avg. meander at centre
Avg. meander at arena edge**
Max. meander
Max. meander at centre
Max. meander at arena edge*
3.6
1.8
0
-1.8
Space exploration
Speed and movetime
Spatial preference
Tracks and stops
Move straightness
B
w1118 Male dro. behavior
Female dro. behavior bench
Acquaintance
Social space distance
Space distance at arena edge
Space distance at arena centre
Space distance at activity episodes
Space distance at inactivity episodes
SSI
SSI at arena edge
SSI at arena centre
SSI at activity episodes
SSI at inactivity episodes
Total interaction duration
Interaction duration at edge
Interaction time prop. at edge
Interaction duration at centre
Interaction time prop. at centre
Interaction duration at activity episodes
Interaction time prop. at activity episodes
Interaction duration at inactivity episodes
Interaction time prop. at inactivity episodes
Total interaction duration at long−stop
Interaction time prop. at long−stop
Interaction episode count**
Interaction episode duration*
Avg. number of crowded dro.
Degree assortativity coefficient
Clustering coefficient**
Betweenness centrality***
Network diameter
Network degree**
Unconnected social network prop.
Global efficiency**
1.2
0
-1.2
-2.4
Social space
Social network topology
Interaction

C

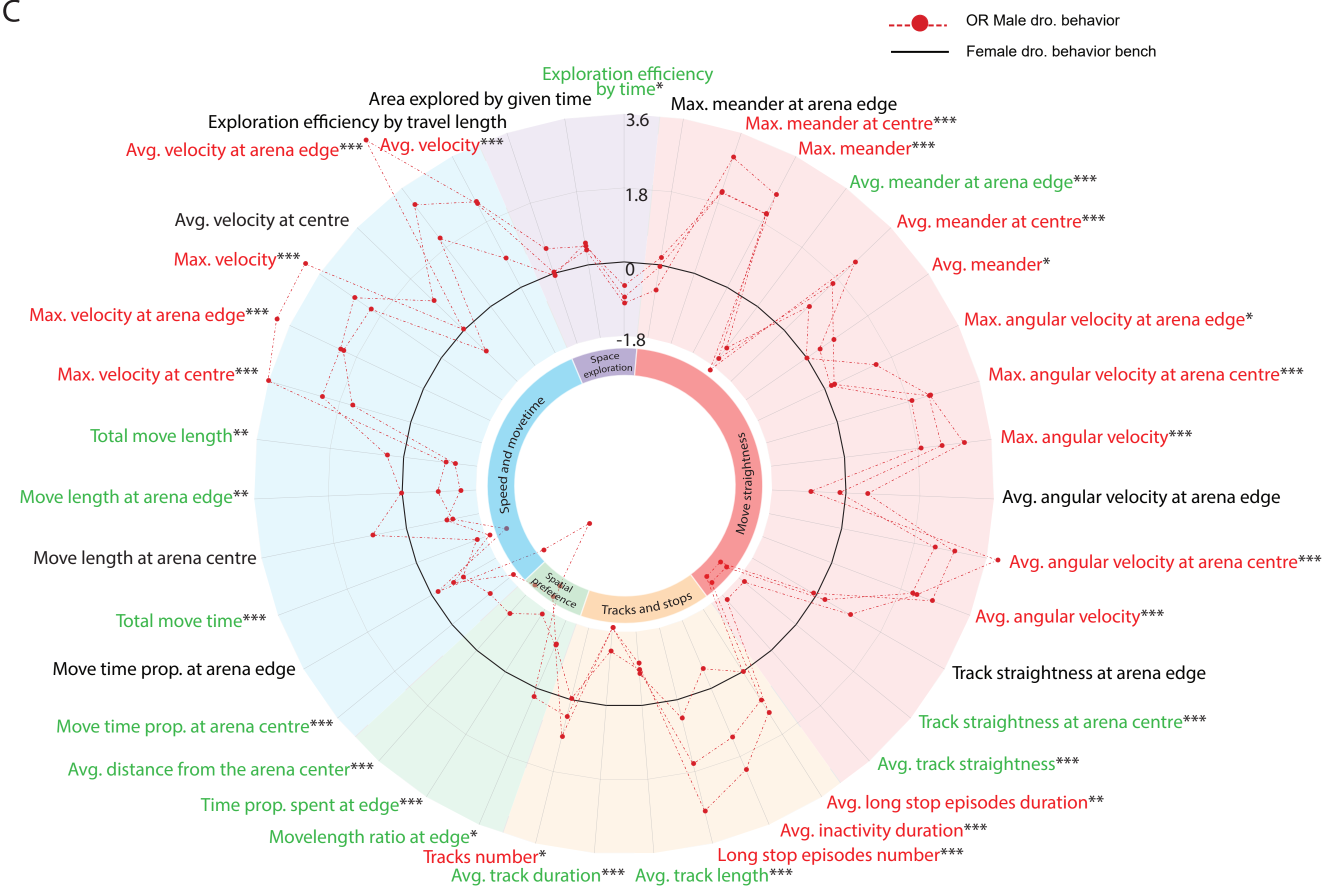

OR Male dro. behavior
Female dro. behavior bench
Exploration efficiency by time*
Area explored by given time
Max. meander at arena edge
Max. meander at centre***
Max. meander***
Avg. meander at arena edge***
Avg. meander at centre***
Avg. meander*
Max. angular velocity at arena edge*
Max. angular velocity at arena centre***
Max. angular velocity***
Avg. angular velocity at arena edge
Avg. angular velocity at arena centre***
Avg. angular velocity***
Track straightness at arena edge
Track straightness at arena centre***
Avg. track straightness***
Avg. long stop episodes duration**
Avg. inactivity duration***
Long stop episodes number***
Avg. track length***
Avg. track duration***
Tracks number*
Movelength ratio at edge*
Time prop. spent at edge***
Avg. distance from the arena center***
Move time prop. at arena centre***
Move time prop. at arena edge
Total move time***
Move length at arena centre
Move length at arena edge**
Total move length**
Max. velocity at centre***
Max. velocity at arena edge***
Max. velocity***
Avg. velocity at centre
Avg. velocity at arena edge***
Avg. velocity***
Exploration efficiency by travel length
3.6
1.8
0
-1.8
Space exploration
Move straightness
Tracks and stops
Spatial preference
Speed and movetime

D

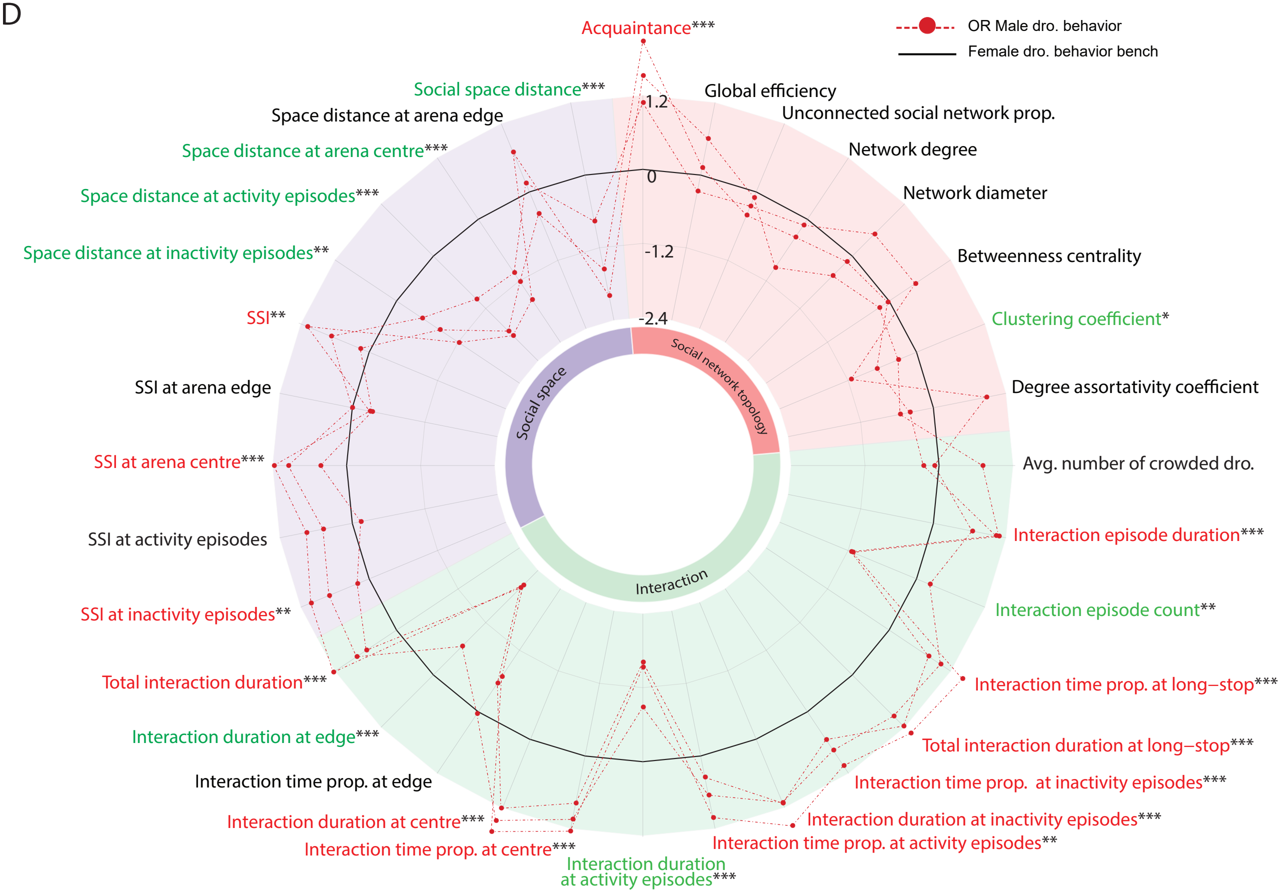

OR Male dro. behavior
Female dro. behavior bench
Acquaintance***
Global efficiency
Unconnected social network prop.
Network degree
Network diameter
Betweenness centrality
Clustering coefficient*
Degree assortativity coefficient
Avg. number of crowded dro.
Interaction episode duration***
Interaction episode count**
Interaction time prop. at long–stop***
Total interaction duration at long–stop***
Interaction time prop. at inactivity episodes***
Interaction duration at inactivity episodes***
Interaction time prop. at activity episodes**
Interaction duration at activity episodes***
Interaction time prop. at centre***
Interaction duration at centre***
Interaction time prop. at edge
Interaction duration at edge***
Total interaction duration***
SSI at inactivity episodes**
SSI at activity episodes
SSI at arena centre***
SSI at arena edge
SSI**
Space distance at inactivity episodes**
Space distance at activity episodes***
Space distance at arena centre***
Space distance at arena edge
Social space distance***
1.2
0
-1.2
-2.4
Social network topology
Social space
Interaction

E

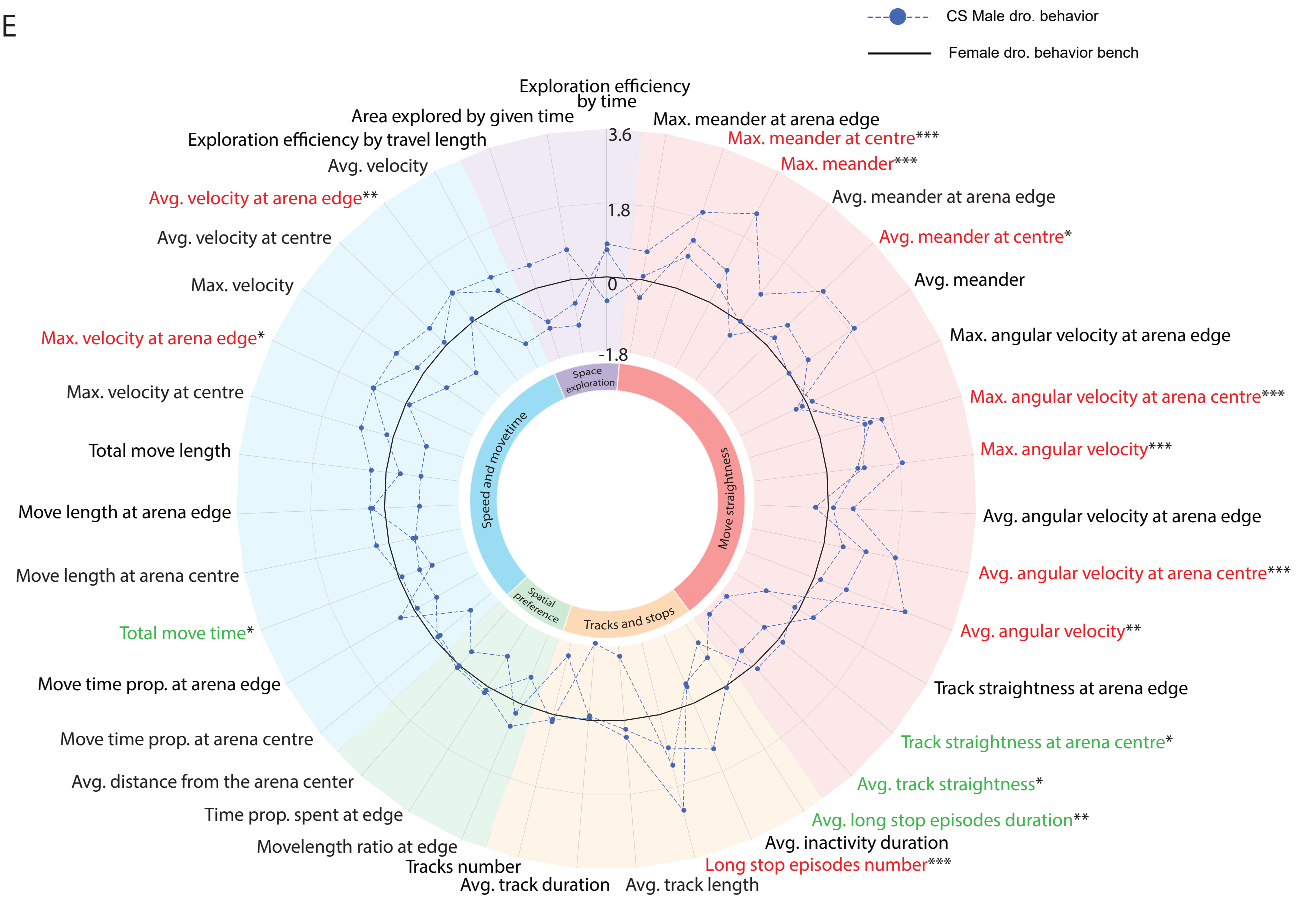
CS Male dro. behavior
Female dro. behavior bench
Exploration efficiency by time
Area explored by given time
Exploration efficiency by travel length
Avg. velocity
Avg. velocity at arena edge**
Avg. velocity at centre
Max. velocity
Max. velocity at arena edge*
Max. velocity at centre
Total move length
Move length at arena edge
Move length at arena centre
Total move time*
Move time prop. at arena edge
Move time prop. at arena centre
Avg. distance from the arena center
Time prop. spent at edge
Movelength ratio at edge
Tracks number
Avg. track duration
Avg. track length
Long stop episodes number***
Avg. inactivity duration
Avg. long stop episodes duration**
Avg. track straightness*
Track straightness at arena centre*
Track straightness at arena edge
Avg. angular velocity**
Avg. angular velocity at arena centre***
Avg. angular velocity at arena edge
Max. angular velocity***
Max. angular velocity at arena centre***
Max. angular velocity at arena edge
Avg. meander
Avg. meander at centre*
Avg. meander at arena edge
Max. meander***
Max. meander at centre***
Max. meander at arena edge
3.6
1.8
0
-1.8
Space exploration
Move straightness
Tracks and stops
Spatial preference
Speed and movetime

F

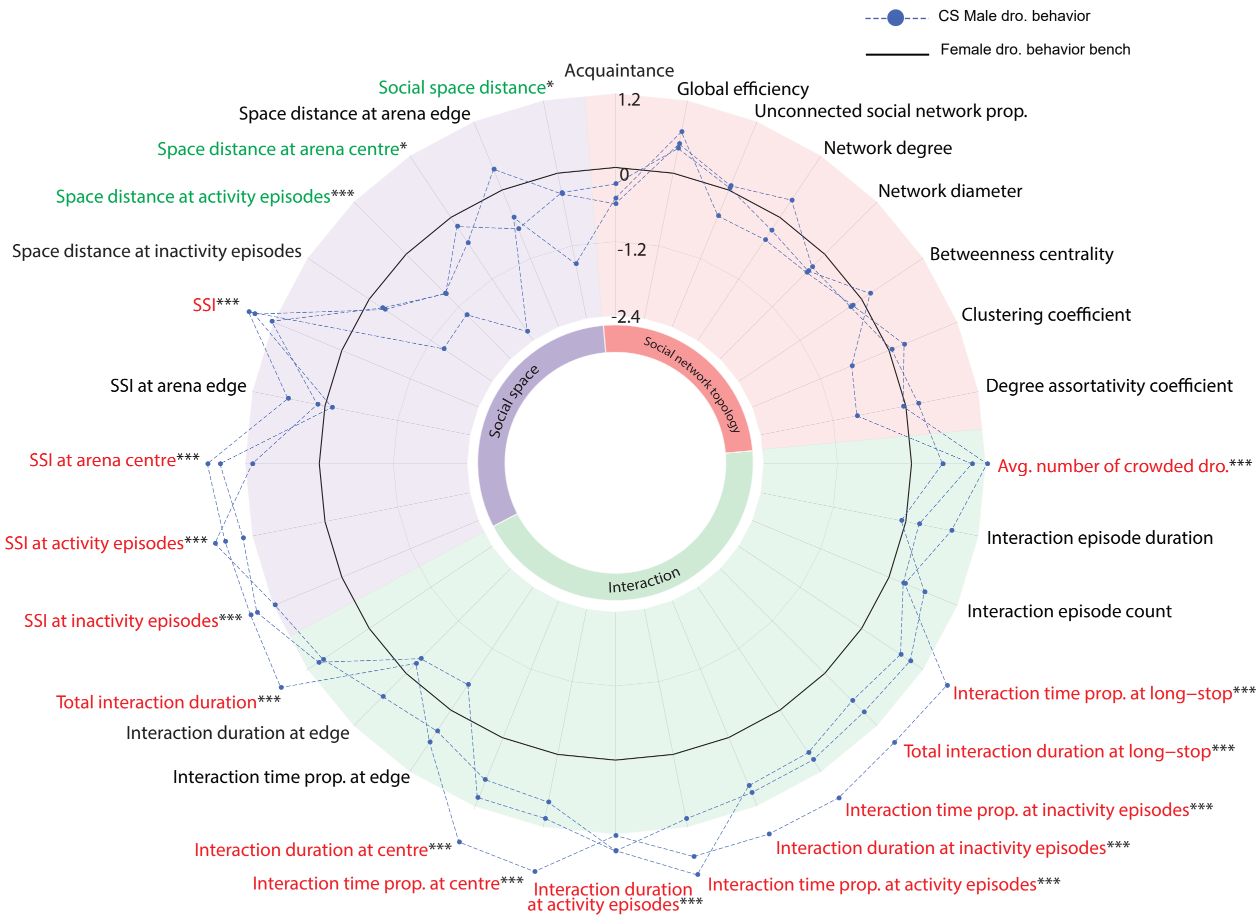
CS Male dro. behavior
Female dro. behavior bench
Acquaintance
Global efficiency
Unconnected social network prop.
Network degree
Network diameter
Betweenness centrality
Clustering coefficient
Degree assortativity coefficient
Avg. number of crowded dro.***
Interaction episode duration
Interaction episode count
Interaction time prop. at long–stop***
Total interaction duration at long–stop***
Interaction time prop. at inactivity episodes***
Interaction duration at inactivity episodes***
Interaction time prop. at activity episodes***
Interaction duration at activity episodes***
Interaction time prop. at centre***
Interaction duration at centre***
Interaction time prop. at edge
Interaction duration at edge
Total interaction duration***
SSI at inactivity episodes***
SSI at activity episodes***
SSI at arena centre***
SSI at arena edge
SSI***
Space distance at inactivity episodes
Space distance at activity episodes***
Space distance at arena centre*
Space distance at arena edge
Social space distance*
1.2
0
-1.2
-2.4
Social network topology
Interaction
Social space
